# Cyclometalated Au^III^ Complexes for Cysteine Arylation in Zinc Finger Protein Domains: towards Controlled Reductive Elimination

**DOI:** 10.1002/chem.201901535

**Published:** 2019-05-09

**Authors:** Margot N. Wenzel, Riccardo Bonsignore, Sophie R. Thomas, Didier Bourissou, Giampaolo Barone, Angela Casini

**Affiliations:** ^1^ School of Chemistry Cardiff University Main Building, Park Place CF10 3AT Cardiff UK; ^2^ CNRS/Université Paul Sabatier Laboratoire Hétérochimie Fondamentale et Appliquée (LHFA, UMR 5069) 118 Route de Narbonne 31062 Toulouse Cedex 09 France; ^3^ Dipartimento di Scienze e Tecnologie Biologiche, Chimiche e Farmaceutiche Università di Palermo Viale delle Scienze, Edificio 17 90128 Palermo Italy

**Keywords:** catalysis, cysteine arylation, gold complexes, reductive elimination, zinc finger proteins

## Abstract

With the aim of exploiting the use of organometallic species for the efficient modification of proteins through C‐atom transfer, the gold‐mediated cysteine arylation through a reductive elimination process occurring from the reaction of cyclometalated Au^III^ C^N complexes with a zinc finger peptide (Cys_2_His_2_ type) is here reported. Among the four selected Au^III^ cyclometalated compounds, the [Au(C^CO^N)Cl_2_] complex featuring the 2‐benzoylpyridine (C^CO^N) scaffold was identified as the most prone to reductive elimination and Cys arylation in buffered aqueous solution (pH 7.4) at 37 °C by high‐resolution LC electrospray ionization mass spectrometry. DFT and quantum mechanics/molecular mechanics (QM/MM) studies permitted to propose a mechanism for the title reaction that is in line with the experimental results. Overall, the results provide new insights into the reactivity of cytotoxic organogold compounds with biologically important zinc finger domains and identify initial structure–activity relationships to enable Au^III^‐catalyzed reductive elimination in aqueous media.

Selective biomolecule modification is an important chemical biology tool for manipulating the properties of biomolecules and investigating their functions in complex biological systems.^1^ Transition‐metal complexes render numerous new organic transformation reactions possible and have been extensively used in organic synthesis. Recently, promising strategies have been developed to apply transition‐metal complexes to bioconjugation. The peculiar reactivity and selectivity of such complexes significantly broaden the scope of the chemical reaction tool boxes for biomolecule modification.^2^ Thus, the past decade has witnessed new ways of tagging proteins with fluorophores or other probes based on palladium‐mediated reactions that played a major role in modern organic synthesis, such as the Suzuki–Miyaura, Mizoroki–Heck, and Sonogashira cross‐coupling reactions.^3^

Although quite attractive, the palladium‐mediated creation of C−C or C−X bonds involving proteins inside living cells as coupling partners remains challenging, being plagued by unproductive interactions of the complex with endogenous functional groups, as in the case of copper‐mediated bioconjugation processes.^4^ To address these limitations, efforts have been made to design transition‐metal complexes of reduced fragility in the biological milieu, including the use of palladium nanoparticles.^5^

In this context, gold complexes have also emerged as extremely promising, being endowed with excellent reactivity and selectivity, compatibility with aqueous reaction medium, and mild reaction conditions.^6^, ^7^ Following the strategy of introducing aryl moieties in proteins with the aid of aryl transition‐metal reagents, Wong and co‐workers have tackled the possibility of forming C−S bonds by derivatizing the sulfhydryl group in cysteines,^8^ as an alternative to the *N*‐methylmaleimide cysteine ligation, given the lack of stability in physiological environments of the maleimide adducts.^9^

In proof‐of‐concept experiments, exposure of different peptidic domains at 37 °C for 24 h to an equimolar amount of the Au^III^ C^N complex **A**, featuring a *N*,*N′*‐bis(methanesulfonyl)ethylene ancillary ligand (Figure 1), produced remarkable conversions in the corresponding aryl thioethers.^8^ Under these conditions, the reaction did not stop at the gold–peptide adducts, but produced the arylated product resulting from C−S reductive elimination. The excellent chemoselectivity of the reaction was further demonstrated in bioconjugation reactions targeting the surface exposed cysteine residue of serum albumin, with the aid of a dansyl‐linked Au^III^ C^N derivative of **A**.^8^

**Figure 1:**
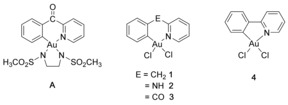
Structure of the cyclometalated Au^III^ C^N complexes **A** and **1**–**4** studied for their Cys arylation properties.

With the aim of developing selective protein binders, the selectivity of Au^III^ C^N complexes to the zinc finger domain of PARP‐1 was studied using a hyphenated mass spectrometry approach combined with quantum mechanics/molecular mechanics (QM/MM) studies.^10^ The nature of the Au^III^ peptide bound fragments was identified, and under the applied experimental conditions the most reactive and selective compound **1** [Au(C^CH2^N)Cl_2_] formed mainly peptide‐[Au^III^C^CH2^N)] adducts. Notably, the compound showed, in competition experiments, the highest selectivity for the cysteine‐rich zinc finger PARP‐1 (Cys_2_HisCys type) with respect to the Cys_2_His_2_ type of domain.^10^

The reported binding selectivity towards a specific zinc finger domain could never be observed for Au^III^ coordination complexes featuring chelating N^N ligands;^11^, ^12^ therefore, highlighting the great potential of organometallic Au^III^ C^N complexes as targeted protein binders.

More recently, Farrell and co‐workers have further described the reaction of the Au^III^ C^N complex **1** with the full‐length zinc finger (Cys_3_His) of HIV nucleocapsid protein NCp7 resulting in C−S aryl transfer from the Au^III^ organometallic species to a cysteine of the zinc finger.^13^ The Cys arylation was observed by mass spectrometry after 48 h incubation with the peptide.

Intrigued by these observations, we decided to further explore the reactivity of Au^III^ C^N complexes with a model of Cys_2_His_2_ zinc finger domain (ZF). The library of compounds was expanded to include **1**, **2** [Au(C^NH^N)Cl_2_] (C^NH^N=*N*‐phenylpyridin‐2‐amine,) and **3** [Au(C^CO^N)Cl_2_] (C^CO^N=2‐benzoylpyridine) (Figure 1) with the aim to gain more insight into the factors controlling the key C−S coupling reaction. Each gold complex was incubated separately with the ZF peptide in a 3:1 ratio in (NH_4_)_2_CO_3_ buffer (25 mm, pH 7.4) and the samples analyzed at different incubation times (10 min and 24 h at 37 °C) by high‐resolution electrospray ionization mass spectrometry (HR‐LC‐ESI‐MS) following previously reported procedures.^10^ The MS studies were further supported by circular dichroism (CD) spectroscopy, which confirmed the proper folding of the holo‐ZF domain^14^ as well as the loss of the typical secondary structure upon addition of complex **3** in buffered aqueous solution (Figure S1, Supporting Information).

The HR‐LC‐ESI‐MS results showed that all compounds react quickly, but distinctively, with the ZF domain by displacing zinc from the coordination site. After 10 min, all three complexes form classical apo‐ZF‐[Au^III^(C^N)] adducts, and two or even three of these adducts can be detected, demonstrating the presence of multiple Au binding sites (Table S1 and Figures S2–S4, Supporting Information). Only complex **3** induced cysteine arylation forming also apo‐ZF‐[C^CO^N] species as a result of C−S reductive elimination (Table S1, Figure S4, Supporting Information).

Figure 2 reports the HR‐LC‐ESI‐MS spectra obtained for complexes **1**–**3** after 24 h incubation with the ZF domain. At longer incubation times, the results show the coexistence of apo‐ZF‐[Au^III^(C^N)] adducts and arylation products apo‐ZF‐[C^N]. In the case of **3**, the MS spectrum shows the presence of an adduct of the type [apo‐ZF+3(C^CO^N)]^*n*+^ at *m*/*z* 584.7598 (*n*=6), indicating the existence of up to three arylation sites on the peptide. It is likely that in excess of metal complex, amino acid side chains other than those of the two Cys, may be arylated. In previous tandem MS/MS experiments, we assessed the likely binding sites for compound **1** on the ZF model.^10^ Specifically, the fragmentation pattern of the apo‐ZF‐[Au^III^C^CH2^N] adduct suggested Au^III^ coordination to Cys4 and Cys7 as well as to His24. Thus, the nitrogen of His24 may be another arylation site. In fact, Au^III^–aryl species have been unequivocally identified as reactive intermediates in oxidant‐free C−N cross coupling reactions.^15^, ^16^ Overall, in the present study, the analysis of the reaction products indicates that **3** is the most prone to reductive elimination, followed by **1** and **2**. The latter is also the one forming mainly mono‐adducts apo‐ZF‐[Au^III^(C^NH^N)].

**Figure 2:**
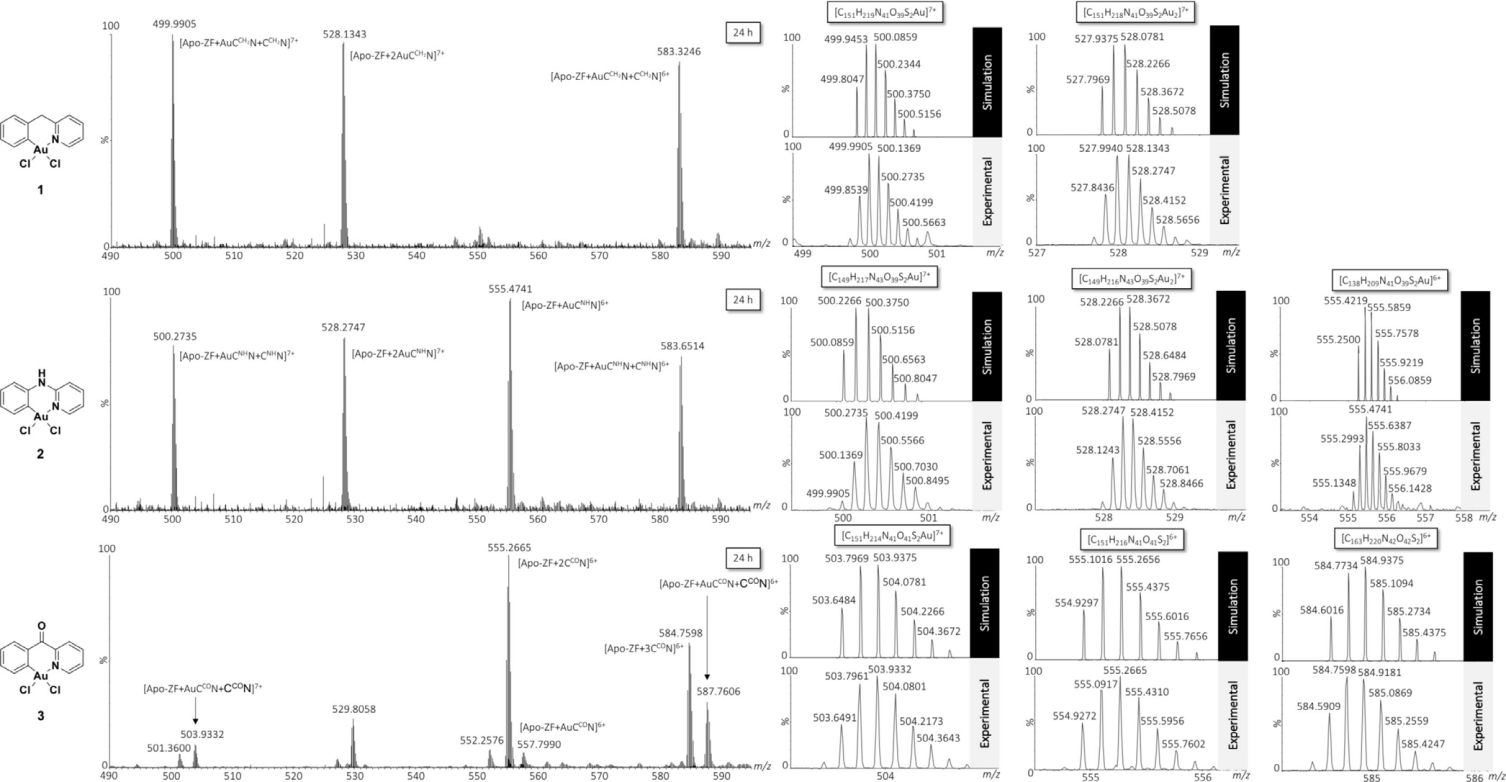
HR‐LC‐ESI‐MS spectra of the reaction of Au^III^ C^N complexes **1**–**3** with the Zn–ZF domain (3:1 ratio) after 24 h incubation at 37 °C in (NH_4_)_2_CO_3_ buffer (25 mm, pH 7.4). Comparisons between experimental and theoretical isotopic pattern distribution for selected adducts. In each simulated isotopic distribution Au ions were considered in the oxidation state 3+. The ZF sequence is ^1^PYKCPECGKSFSQKSDLVKHQRTHTG^26^.

Afterwards, the five‐membered cyclometalated Au^III^ complex **4** [Au(phepy)Cl_2_] (phepy=2‐phenyl‐pyridinato) was also tested to investigate the influence of a different C^N scaffold on the reactivity with the ZF. In this case, the complex formed mainly mono‐adducts of the type ZF‐[Au^III^(C^N)] already after 10 min incubation, which remained stable after 24 h (Table S1 and Figure S5, Supporting Information). No indication of any reductive elimination products could be detected.

It is worth mentioning that when we repeated the experiments under the same conditions, but reacting the compounds with Cys instead of the ZF domain, we were not able to detect any Cys arylation product (data not shown), in line with previously reported studies.^17^ This result indicates that for the reductive elimination to take place following Au^III^‐thiol adduct formation, the entire metal complex‐peptide adduct is crucial. We surmised that the peptide provides multiple gold binding sites which template the arylation reaction.

Reductive elimination plays a major role in transition‐metal‐mediated reactions (cross‐couplings in particular). It is the key product‐releasing step of many transformations. In contrast to oxidative addition, the feasibility of reductive elimination at gold has never been questioned and it was demonstrated experimentally early on.^6^ Nevertheless, our knowledge on reductive elimination at gold remains rather limited, especially for reactions arising in aqueous environment, and involving peptides as substrates.^18^

Thus, to rationalize the results of the experimental investigations, DFT calculations were performed with complexes **1**–**4**. Due to the strong preference for reductive elimination to occur between groups located in *cis* position, it is very likely that the cysteine arylation involves Au^III^ C^N complexes as key intermediates with the aryl group and a cysteinate in *cis* arrangement. It must be noted that previously reported results by tandem MS identified Cys4 as the most stable Au binding site for complexes **1** and **2** in the selected ZF domain.^10^ Thus, our mechanistic hypothesis, according to which at least one of the chlorido ligands is replaced by a cysteinate prior to reductive elimination, is in line with the experimental observation.

Due to the high electronic dissymmetry of the C^N chelate (C exerts a much stronger *trans* influence than N),^6^, ^15^ the cysteinate adduct with S in *trans* position to N (and thus, *cis* to the aryl group) is more favored thermodynamically. This stereochemical preference has been supported experimentally in related thiolate complexes,^19^ and the difference in energy with the other diastereomer is large according to DFT calculations (see Figure S6 and Table S2 in the Supporting Information, and Ref. 10 and 20).

The hypothesized mechanism for C−S coupling is depicted in Scheme 1. In the cysteinate Au^III^ complex with the chelate C^N ligand (**R**), the aryl group is almost coplanar with the gold coordination plane, thus, coupling with the adjacent S atom is disfavored.^21^ For the aryl group to participate in reductive elimination, it must rotate. This requires the N atom to decoordinate and the C^N chelate to open. To promote this process, the participation of a second cysteinate residue is envisioned. Apical approach of the S atom induces the displacement of the N atom from gold and leads to the bis‐cysteinate adduct **I** via transition state **TS1**. Reductive elimination then proceeds via transition state **TS2** to give the Cys‐arylated product **P** and the linear Au^I^ complex [CysAuCl]^−^. Complexes **1**–**3** present phenyl and 2‐pyridyl scaffolds linked by CH_2_, NH and CO groups, respectively, whereas the C^N ligand of compound **4** is a 2‐phenyl‐pyridinate, that is, with no linker group between the two aromatic groups.

**Scheme 1:**
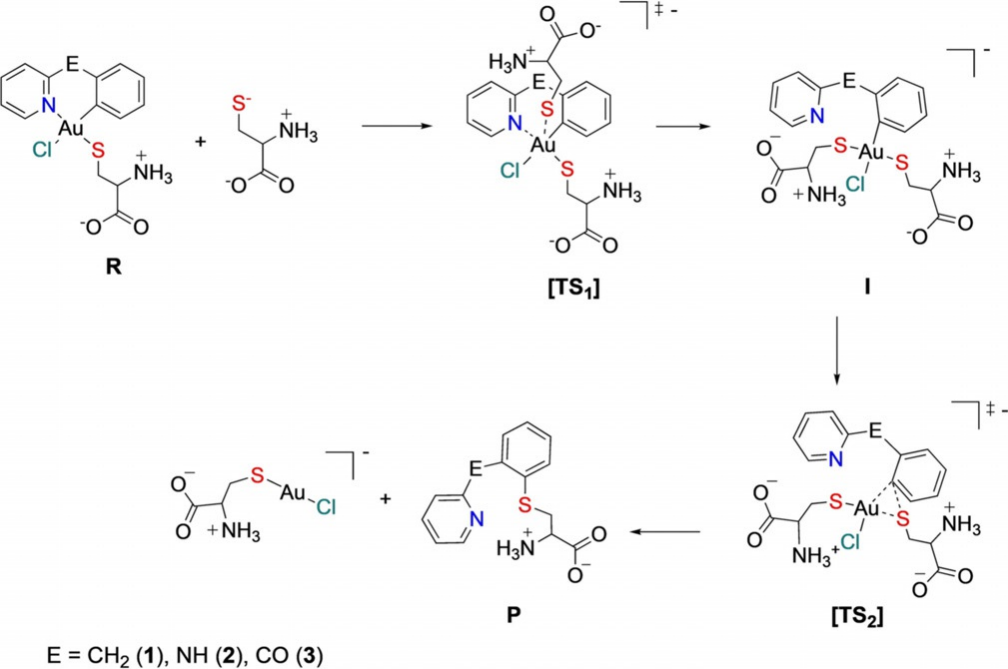
Proposed reaction mechanism for the cysteine arylation reaction (C−S coupling) catalyzed by the Au^III^ C^N complexes.

The species involved and their relative energy values, for the more reactive Au^III^ compound **3**, with carbonyl as bridging group in the chelating ligand, are shown in Figure 3 A. Analogous figures are reported for compounds **1**, **2** and **4** in the Supplementary Information (Figures S7–S9, Supporting Information). The relative energy values and activation barriers are reported in Table 1. The relative energies along the reaction pathways for the four Au^III^ complexes are graphically compared in Figure 3 B. Rather similar reaction profiles were obtained for the three complexes **1**–**3**. The displacement of the pyridine moiety by cysteinate at gold (first step leading to **I**) is exergonic (by 32.4–63.5 kJ mol^−1^) and proceeds with a low energy barrier (7.9–29.4 kJ mol^−1^). The subsequent reductive elimination is favored thermodynamically and appears to be the rate‐determining step with activation barriers of 47.3–75.8 kJ mol^−1^. Closer inspection reveals small but significant differences between the three related C^N ligands. For example, the **TS1** energy values for compounds **2** and **3** are 29.4 vs. 7.9 kJ mol^−1^, respectively. This result suggests that the first reaction step is consistently faster for **3** compared to **2**, in agreement with the higher propensity to give reductive elimination of the former complex. Moreover, the activation barrier for the C−S coupling (*E*_2_^≠^) increases in the order CO (**3**)<CH_2_ (**1**)<NH (**2**). This trend well explains why, in the HR‐LC‐ESI‐MS experiments, cysteine arylation easily occurs with **3**, much less with **1**, and only after 24 h for **2**.

**Figure 3:**
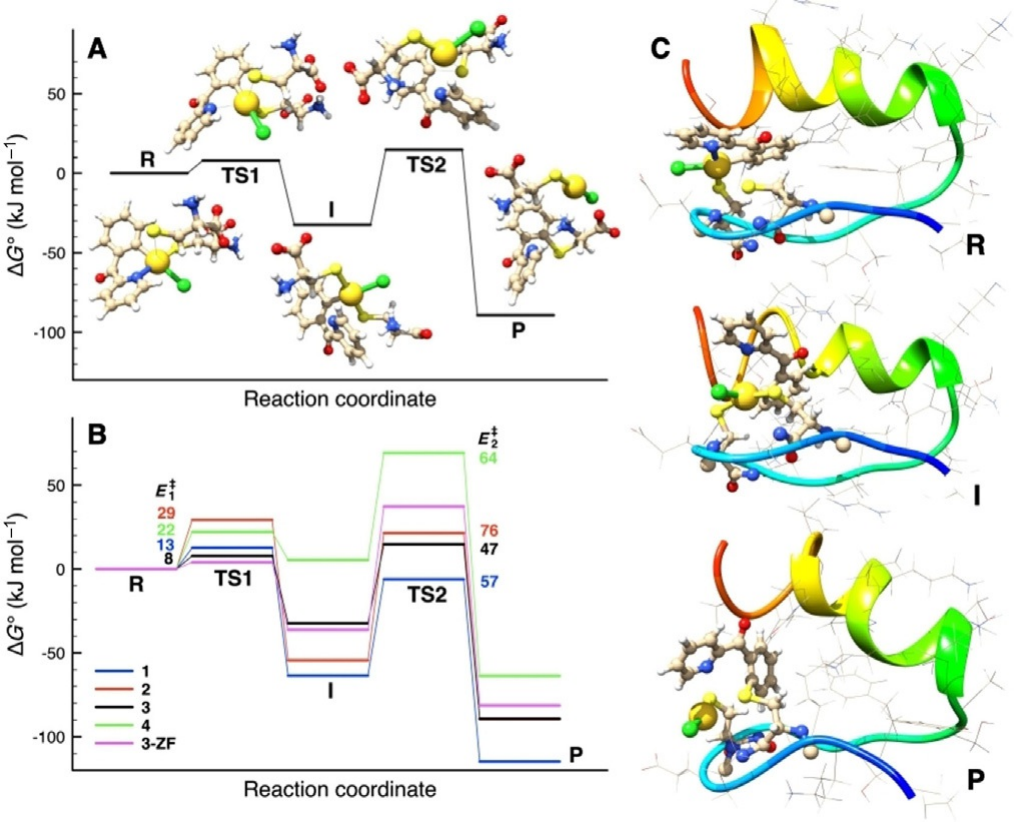
A) species involved along the reaction pathway of the most reactive compound [Au(C^CO^N)Cl_2_] **3**, containing the C=O bridging group in the chelating ligand; structure and energies were obtained by DFT calculations. B) Comparison of the relative energy profiles calculated for the four Au^III^ C^N compounds **1**–**4**; activation energies (*E*_1_^≠^ and *E*_2_^≠^, in kJ mol^−1^) of the first and second reaction steps are indicated for each bridging group in the chelating ligand. C) Structures of the adduct of **3** with the ZF (**3‐ZF**), **R**, **I** and **P**, obtained by QM/MM calculations, with relative energy values also shown in magenta in panel B. The atoms in the lower MM layer are represented in wires. The protein backbone is highlighted in unstructured tube and alpha helix styles.

**Table 1:** Calculated relative standard Gibbs free energy values and the first and second activation barriers (kJ mol^−1^) of the species involved in the proposed reaction pathway of the considered Au^III^ C^N compounds **1**–**4**.

	Compounds				
	**1**	**2**	**3**	**4**	**3/ZF**
**R**	0.0	0.0	0.0	0.0	0.0
**TS1=*E***_**1**_^**≠**^	12.7	29.4	7.9	22.2	4.1
**I**	−63.5	−54.4	−32.4	5.4	−36.2
**TS2**	−6.1	21.4	14.8	69.2	37.2
***E***_**2**_^**≠**^	57.4	75.8	47.3	63.8	73.4
**P**	−114.8	−89.5	−89.2	−63.7	−81.3

As apparent from Figure 3 B, the difference between compounds **1**–**3** results from the stability of the bis‐cysteinate intermediate **I** as compared to the starting complex **R**. This intermediate is less stable with the CO bridging unit and in turn, the following transition state **TS2** is very accessible in energy, which results in a small energy span.

The phenyl‐pyridine complex **4** shows a slightly different reaction energy profile (Figure S9, Supporting Information). The bis‐cysteinate intermediate **I** is comparable in energy to the starting complex **R**. The stronger chelating effect compared to the C^N ligands disfavors N decoordination, but the corresponding activation barrier remains fairly accessible (22.2 kJ mol^−1^). The low stability of intermediate **I** could have resulted in a low energy span and overall easy cysteine arylation process, by extension of that observed for the C^N ligands. However, the transition state **TS2** for reductive elimination lies quite high in energy and the activation barrier for C−S coupling is significantly higher than for complex **3** (63.2 kJ mol^−1^). One possible explanation may be the higher steric demand nearby the C atom of the aryl group to be coupled with the cysteinate residue. In fact, the pyridyl ring is directly linked to the phenyl moiety and may induce some steric shielding. The reaction profile is consistent with the experimental results of complex **4** in which no sign of cysteine arylation has been detected. Enlarged structures of the transition states and of the intermediate of all compounds are reported in Figures S10 and S11 (Supporting Information).

The observed differences in the relative stability of **R** and **I** cannot be simply attributed to the electronic effects the bridging unit exerts on the N atom of the pyridine. Indeed, the withdrawing character of the C=O bridge is expected to decrease the donor strength of N. Therefore, decoordination from the Au^III^ center should be more favored thermodynamically for the N^CO^C ligand, which is not the case. Other factors probably come into play to explain the influence of the C^N ligand. This could involve the π‐conjugation within the different C^N frameworks and how it is affected for the different ligands upon N decoordination (from **R** to **I**). To evaluate the different donicity of the pyridine nitrogen atom and the π‐conjugative strengths, natural bond orbital (NBO) analyses were performed on the starting complexes deriving from **2** and **3** (Figure S12, Supporting Information). Furthermore, we also evaluated possible differences due to steric effects by using non‐covalent interaction (NCI) plots (Figure S13, Supporting Information). Unfortunately, the obtained results do not allow for an unambiguous identification of the differences in steric and/or electronic effects and/or in weak non‐covalent interactions that could explain the different relative stability of the intermediates.

Afterwards, QM/MM calculations were performed to mimic the binding of compound **3** with the ZF domain, and the structure assumed by the system along the reaction pathway. The higher QM layer is composed by **3** and by the Cys35 and Cys38 residues (atoms in balls and sticks in Figure 3 C).

The structures of the transition states involved are reported in Figure S14 (Supporting Information) and their energy values shown in the plot of Figure 3 B. It is interesting to notice that the relative Gibbs free energy values of reactant, intermediate, and product are comparable with those obtained for the model systems reported in Scheme 1 and in Figure 3 A,B.

Cysteine bioconjugation is a powerful tool that allows for the introduction of a diverse array of substrates to biomolecules through formation of covalent linkages. With the aim of expanding the scope, generality and utility of transition‐metal‐mediated thiol arylation through C−S bond formation, reductive elimination processes occurring from robust organometallic Au^III^ complexes have been recently described by some authors,^21^ although only few have explored this reactivity in physiologically relevant conditions.^8^, ^22^

In summary, we have further explored the potential of C^N‐cyclometalated Au^III^ complexes for Cys arylation in protein domains, considering that this reactivity is noteworthy for the ZnCys_2_His_2_ transcription factors. Thus, we have identified initial structure–activity relationships to direct the reactivity of this family of gold complexes towards a certain zinc finger domain, which may lead to controlled reductive elimination in aqueous environment. Moreover, specificity and efficiency in gold–ligand binding and subsequent C−S transfer may be modulated both by the nature of the gold compound and the nucleophilicity and accessibility of the cysteines in the zinc finger core.^23^ The latter contribution may also explain why we could not find any evidence of reductive elimination in the case of the PARP‐1 zinc finger peptide.^10^ Further studies are necessary to fully explore the electronic and steric effects of different ligands on the arylation process, as well as to consider the effects of the protein microenvironment on the stability of the intermediates, possibly influenced by other non‐covalent interactions between the metal complex and the peptide.

## Experimental Section

### General

Solvents and reagents (reagent grade) were all commercially available and used without further purification. The zinc finger precursor peptides were obtained from Peptide Specialty Laboratories GmbH and had the sequence ^1^PYKCPECGKSFSQKSDLVKHQRTHTG^26^ (ZF). Ammonium carbonate, dimethyl sulfoxide (DMSO), zinc acetate dihydrate, water (molecular biology grade) were purchased from Fisher. Dithiothreitol (DTT) was purchased from Alfa Aesar. ^1^H and ^13^C NMR spectra were recorded in [*d_6_*]DMSO solution, with TMS as the internal reference, on Bruker Avance 400 or 500 MHz NMR spectrometers. HR‐ESI‐MS spectra were recorded on Synapt G2‐Si time‐of‐flight (TOF) mass spectrometer (Waters) by high‐pressure liquid chromatography (HPLC). HPLC was performed with an Acquity UPLC system (Waters) and by using an Acquity UPLC protein BEH C4 column (300 Å, 1.7 μm, 2.1 mm×100 mm). Mass spectra were acquired and processed using MassLynx V4.1 (Waters). Compounds **1**–**4** have been synthesized by following procedures already reported in the literature.^19^, ^24^ The purity of the compounds was confirmed by elemental analysis, which showed purity >98 %.

### Mass spectrometry studies

The zinc finger was reconstituted according to a previously published procedure.^12^ In brief, the precursor peptide was incubated with DTT (3 equiv, 3 h) in (NH_4_)_2_CO_3_ (25 mm, pH 7.4) and then with zinc acetate (3 equiv, 30 min) at 37 °C. The formation of the zinc finger was assessed by a mass shift in the resulting mass spectra. Stock solutions of the gold compounds were freshly prepared in DMSO at a concentration of 10 mm. The individual experiments between the gold compounds and the ZF were performed at a molar ratio of 3:1 (gold complex:ZF) with the peptide at a final concentration of 10 μm. The compounds were typically incubated at 37 °C for 10 min and 24 h. Samples were analyzed with a Synapt G2‐Si time‐of‐flight (TOF) mass spectrometer (Waters). The instrumental parameters for high‐pressure liquid chromatography mass spectrometry (HPLC‐MS) were as follows: 2.85 kV capillary voltage, 120 °C source temperature, 350 °C desolvation temperature, 90 L h^−1^ cone gas, 900 L h^−1^ desolvation gas and 6 bar nebulizer. A linear gradient from 95 to 5 % water [0.1 % formic acid (FA)], whereas proportionally increasing acetonitrile (0.1 % FA), in 8 min was used. The flow rate was 300 μL min‐1, the column was held at 40 °C and the autosampler at 20 °C.

### Circular dichroism

Stock solutions of the zinc finger peptide in (NH_4_)CH_3_COO (5 mm, pH 7.4) have been prepared as described for the MS samples; compound **3** was dissolved in propionitrile to afford a 3 mm solution freshly prepared prior to analysis. The evolution of the CD spectra of the peptide [25 μm in (NH_4_)CH_3_COO, 5 mm, pH 7.4] in the presence of 3 equiv of **3** was followed over time (0, 10, 30, and 60 min). CD spectra were recorded with an Applied Photophysic Chirascan spectrometer, from 205 to 300 nm by using the following parameters: temperature: 25 °C; step size: 1 nm; bandwidth: 1 nm; time per point: 0.5 s; repeat: 4.

### Computational studies

DFT calculations were performed on the structures of compounds **1**–**4** and on the species involved in the reaction pathway of the title reaction (see Scheme 1) by following recently reported procedures.^10^, ^20^ The M06‐L DFT functional,^25^ the Lanl2tz(f)^26^ basis set for Au and the 6‐31G(d,p)^27^ basis set for Cl, S, O, N, C, and H atoms were used. Solvent effects were implicitly evaluated by full geometry optimization in the water solvent, reproduced by the polarizable continuum model (PCM).^28^ Transition‐state structures were found by the synchronous transit guided quasi‐Newton method.^29^ Vibration frequency calculations, within the harmonic approximation, were performed to confirm that each optimized geometry corresponded to a minimum or to a first‐order saddle point (for transition‐state structures) in the potential energy surface, and to evaluate their standard Gibbs free energy values, at 298.15 K. The energy values reported in Figure 3 B were obtained by single point calculations on the optimized structures by using the Lanl2tz(f) for Au and expanding the all electron basis set to 6‐311G(d,p)^30^ for all the other atoms.

Quantum mechanics/molecular mechanics (QM/MM) calculations were performed to mimic the binding and reactivity of compound **3** with the ZF domain. The ZF model was obtained by the Protein Data Bank ID: 1MEY, consisting of a crystal structure of a zinc‐finger‐DNA complex. In detail, the aminoacidic sequence 32–57 was extracted and residues 45 and 49 changed to K and V, respectively, with the Maestro software,^31^ to make the sequence exactly matching the ZF used in our study. The Zn^2+^ ion was removed and the M06‐L DFT functional was used in the QM layer (atoms in balls and sticks in Figure 3 C), composed by the Cys35 and Cys38 residues of the ZF and by the compound **3**. The UFF force field^32^ was used in the MM layer (atoms in wires). Full geometry optimization was followed by a frequency analysis, to confirm that the obtained structure corresponded to an energy minimum in the potential energy surface. The transition state structures of the **3**‐ZF complex were found by a partial geometry optimization procedure within the QM/MM method. In detail, the N−Au and S−Au distances in **TS1**, and S−C distance in **TS2**, were kept constant at the same values obtained in TS1 and TS2 for compound **3**, All calculations were performed by the Gaussian 09 program package.^33^

Non covalent interactions on the intermediates **I** of **2** and **3** were evaluated by using the NCIPLOT program package.^34^ Natural Bond Orbital (NBO) population analysis was performed on the reagents **R** of **2** and **3** by using the NBO subroutine^35^ implemented in Gaussian 09. NBO and NCI pictures (Figure S11 and S12, respectively) were reproduced with the Avogadro^36^ and VMD^37^ software, respectively.

## Conflict of interest

The authors declare no conflict of interest.

## Supporting information

As a service to our authors and readers, this journal provides supporting information supplied by the authors. Such materials are peer reviewed and may be re‐organized for online delivery, but are not copy‐edited or typeset. Technical support issues arising from supporting information (other than missing files) should be addressed to the authors.

Supplementary
