# Supplementary material for: Cyclometalated AuIII Complexes for Cysteine Arylation in Zinc Finger Protein Domains: towards Controlled Reductive Elimination
Source: Chemistry. 2019 May 9;25(32):7628–34. doi: 10.1002/chem.201901535 (PMC6594228; doi:10.1002/chem.201901535)
Supplement: Supplementary file 1 — Supplementary [file CHEM-25-7628-s001.pdf]

CHEMISTRY
A European Journal

Supporting Information

Cyclometalated Au^{III} Complexes for Cysteine Arylation in Zinc Finger Protein Domains: towards Controlled Reductive Elimination

Margot N. Wenzel+,[a] Riccardo Bonsignore+,[a] Sophie R. Thomas,[a] Didier Bourissou,[b] Giampaolo Barone,*[c] and Angela Casini*[a]

chem_201901535_sm_miscellaneous_information.pdf

Figures

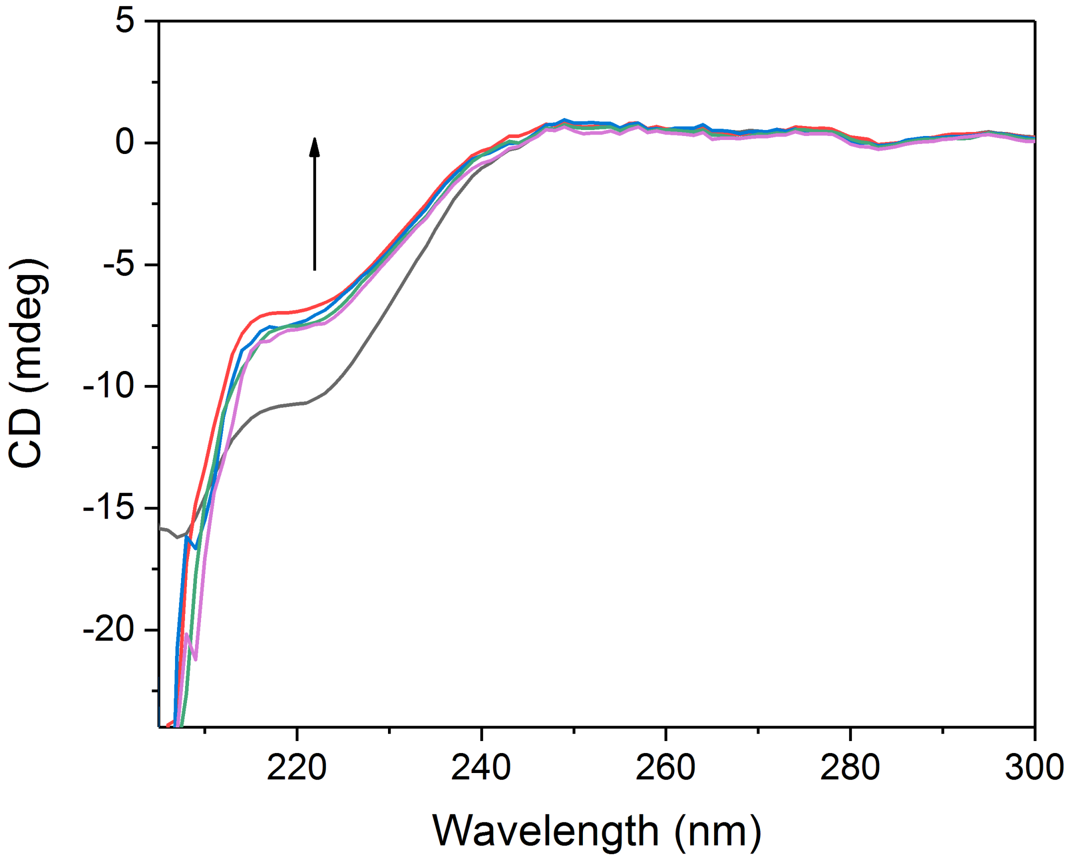

Figure S1 – CD spectra of the Zn-ZF domain (25 µM in NH_4CH_3COO, 5 mM, pH = 7.4) without (grey line) and in presence of 3 equiv. of Au(III)$C^{CO}N$ complex **3** after 0 (red line), 10 (blue line), 30 (green line) and 60 minutes (magenta line).

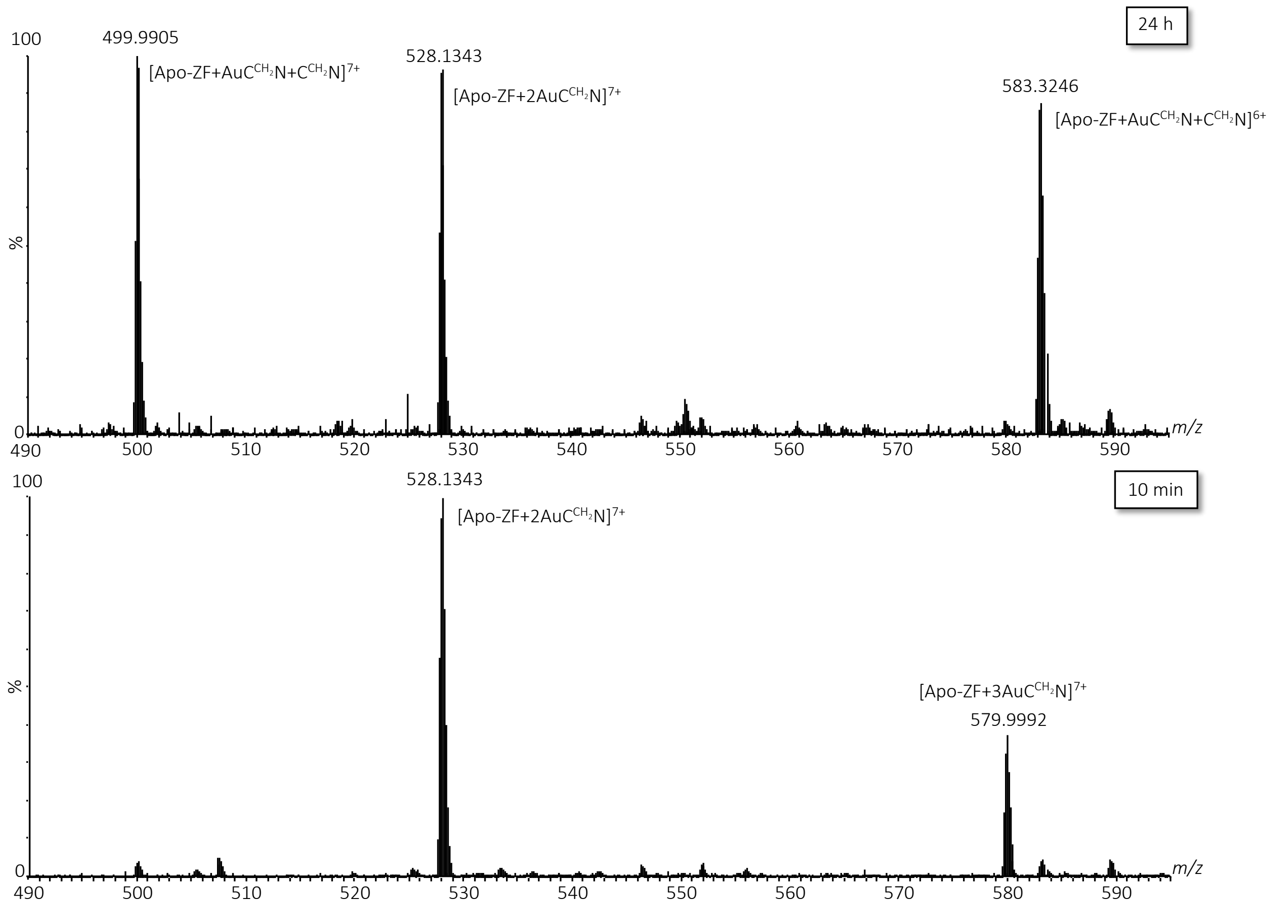

Figure S2 – HR-LC-ESI-MS spectra of the reaction of Au(III)$C^{CH2}N$ complex **1** with the Zn-ZF domain (3:1 ratio) after 10 min or 24 h incubation at 37°C in $(NH_4)_2CO_3$ buffer (25 mM, pH 7.4).

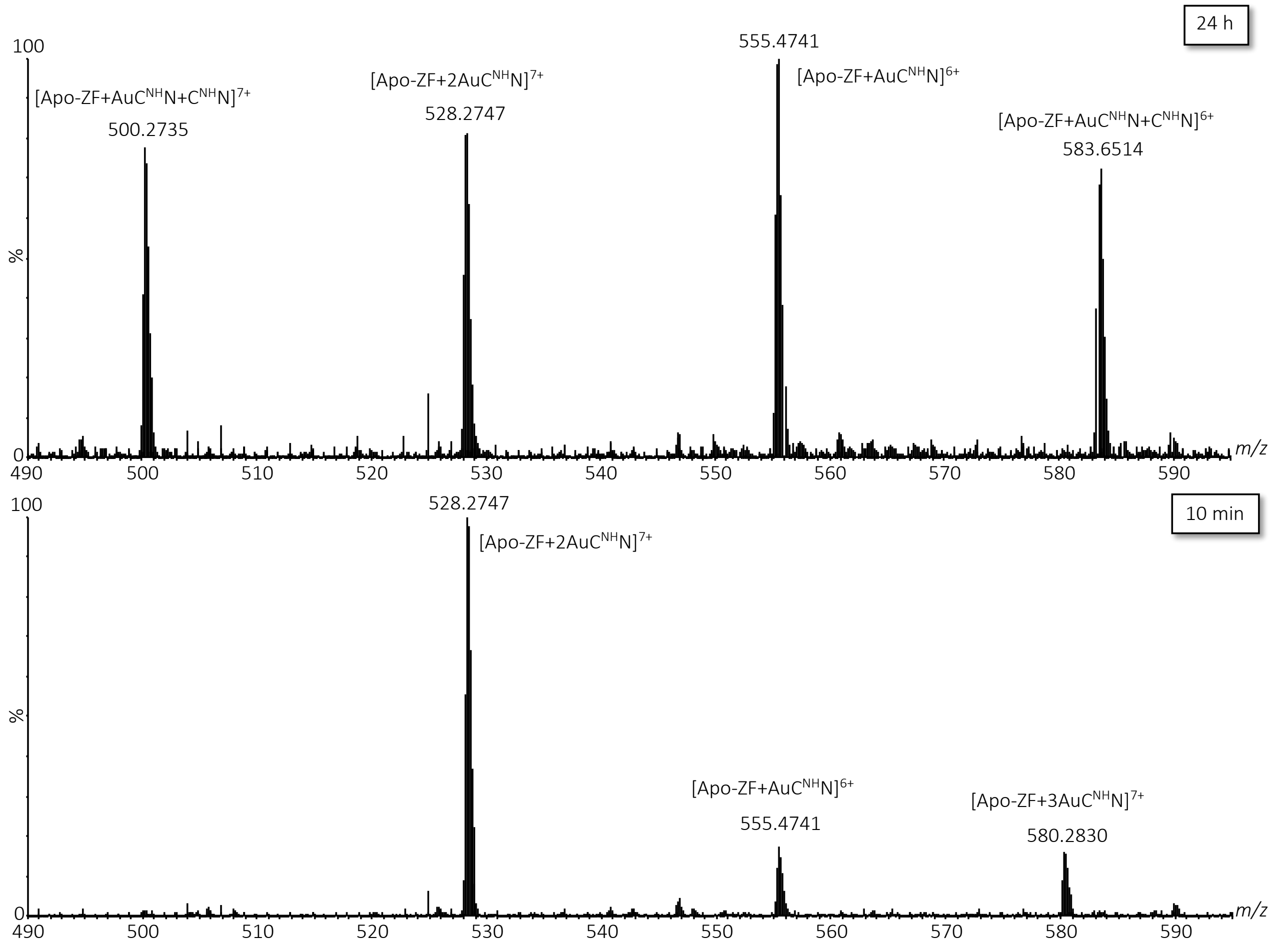

Figure S3 – HR-LC-ESI-MS spectra of the reaction of Au(III)C^{NH}N complex **2** with the Zn-ZF domain (3:1 ratio) after 10 min or 24 h incubation at 37°C in $(NH_4)_2CO_3$ buffer (25 mM, pH 7.4).

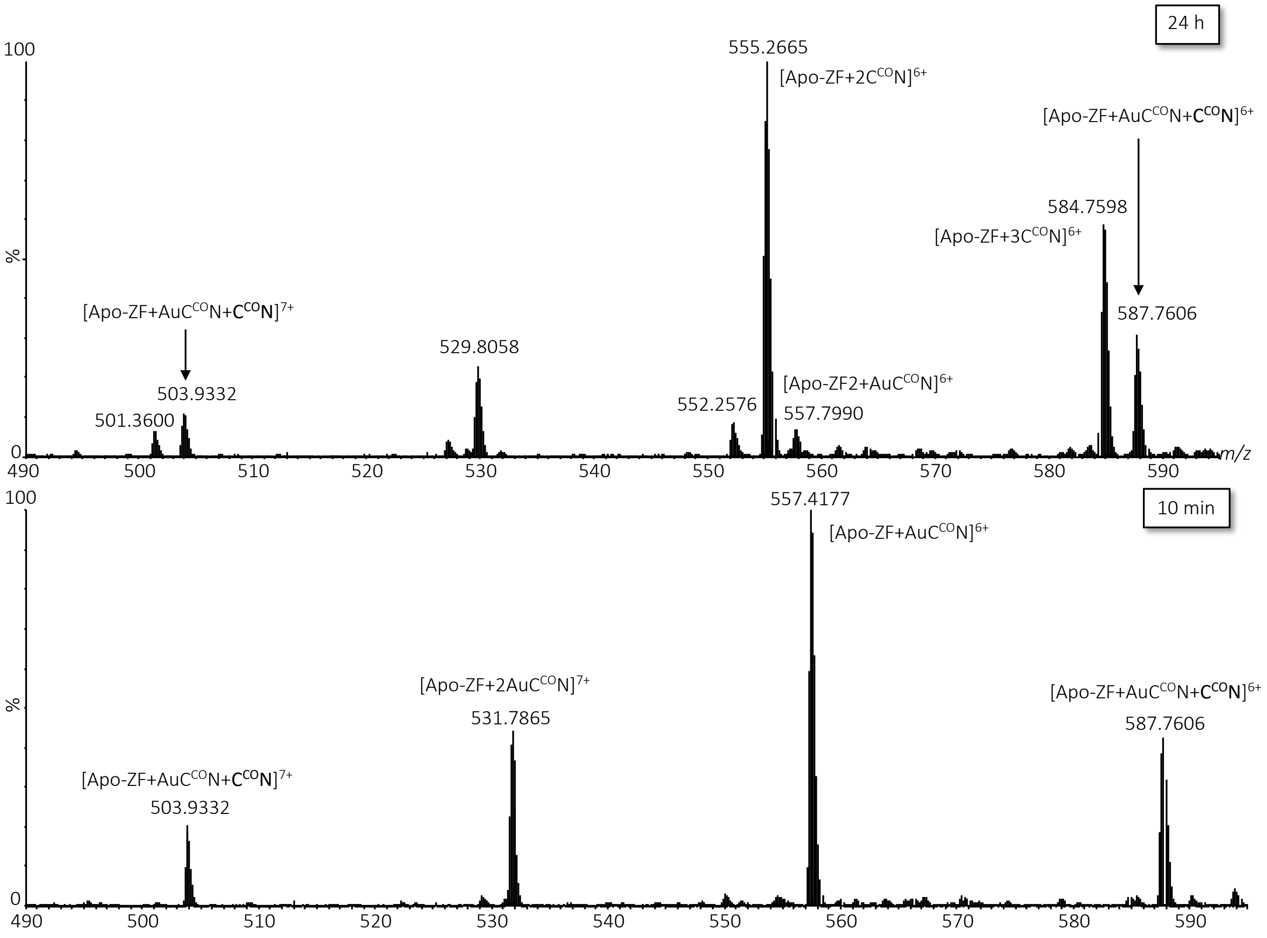

Figure S4 - HR-LC-ESI-MS spectra of the reaction of Au(III)$C^{CO}N$ complex **3** with the Zn-ZF domain (3:1 ratio) after 10 min or 24 h incubation at 37°C in $(NH_4)_2CO_3$ buffer (25 mM, pH 7.4).

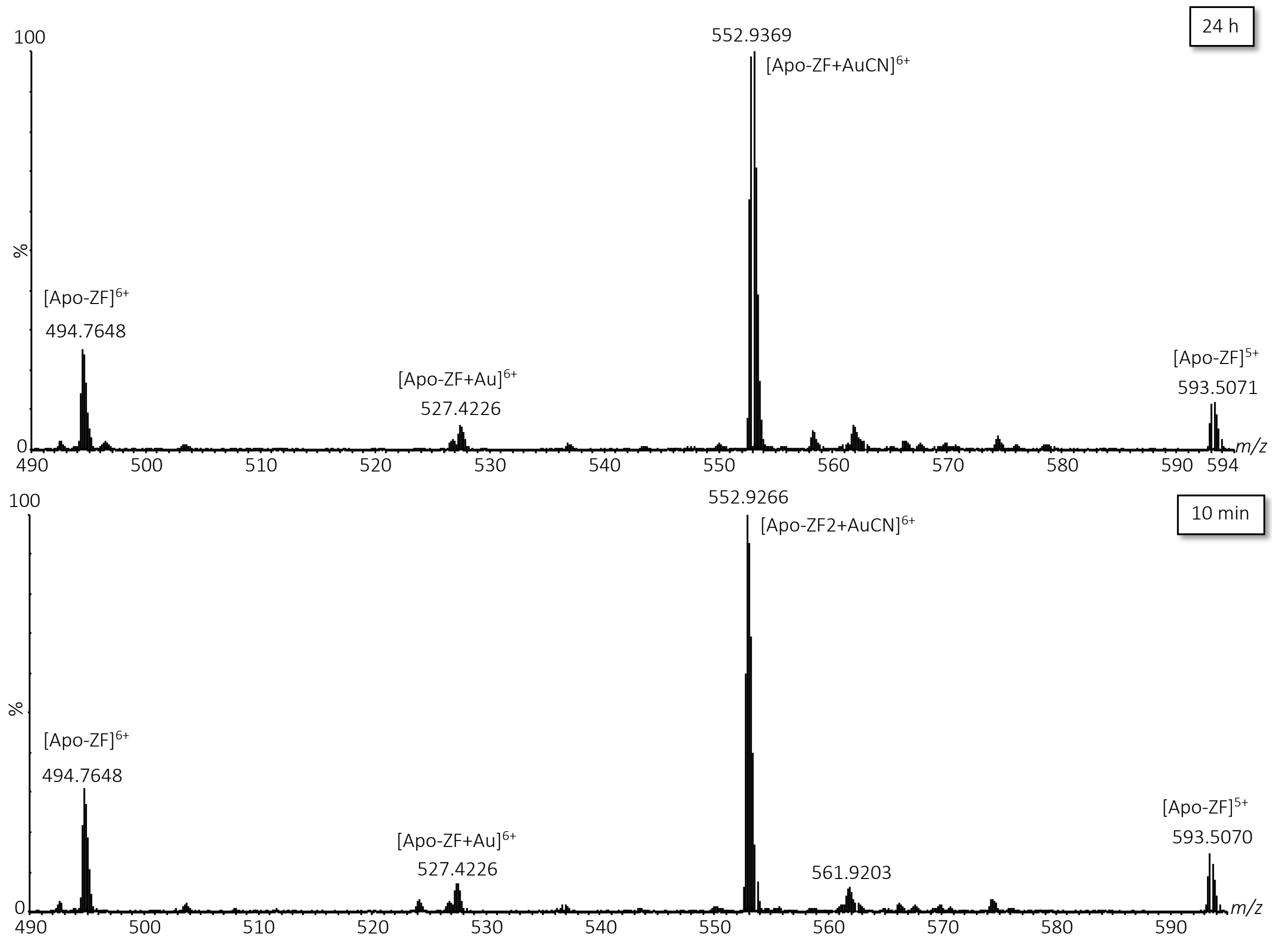

Figure S5 - HR-ESI-MS spectra of the reaction of Au(III)CN complex **4** with the Zn-ZF domain (3:1 ratio) after 10 min or 24 h incubation at 37°C in $(NH_4)_2CO_3$ buffer (25 mM, pH 7.4).

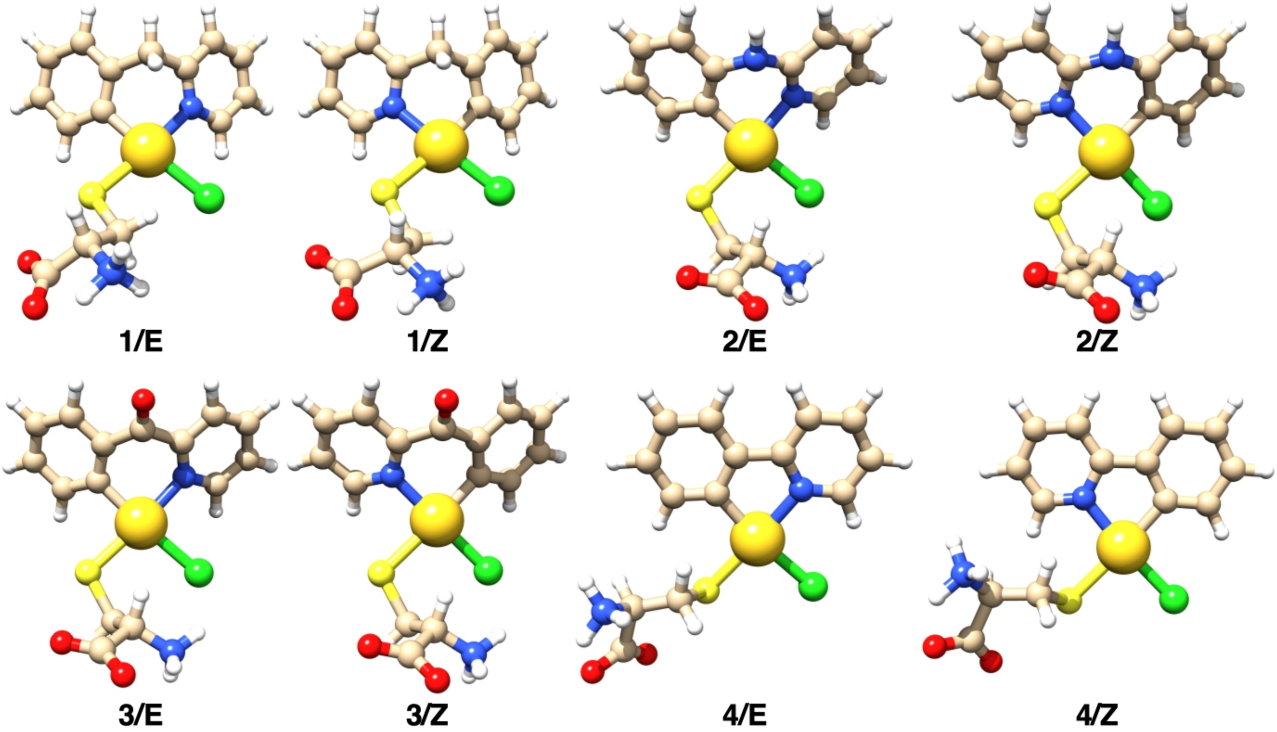

Figure S6. Structures of the Au(III) complexes **1-4** with one cysteinate ligand, obtained by DFT calculations.

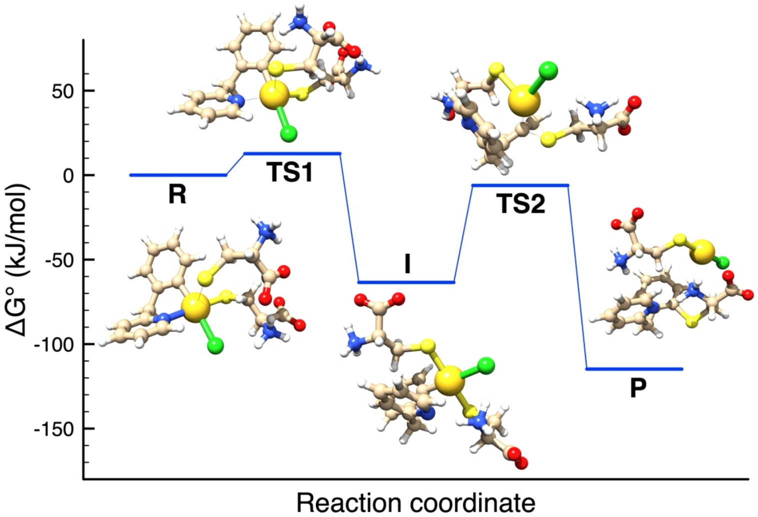

Figure S7 - Species involved along the reaction pathway of the Au(III) complex **1** with two cysteinate ligands. Structure and energies have been obtained by DFT calculations.

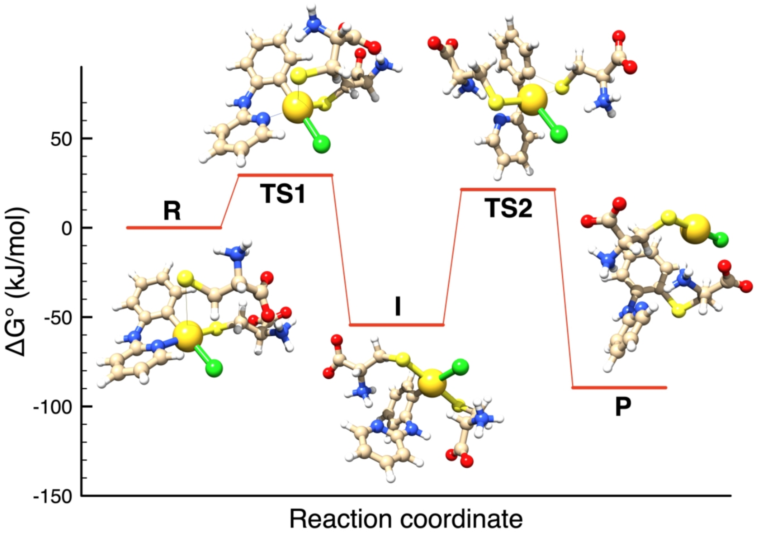

Figure S8 - Species involved along the reaction pathway of the Au(III) complex **2** with two cysteinate ligands. Structure and energies have been obtained by DFT calculations.

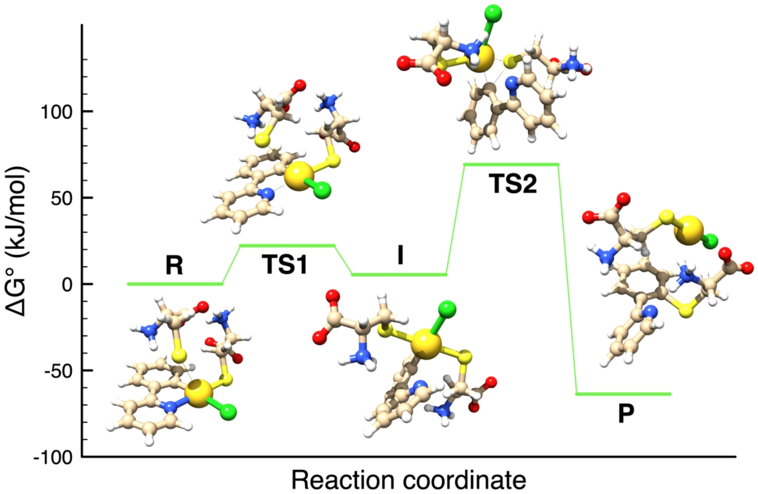

Figure S9. Species involved along the reaction pathway of the Au(III) complex **4** with two cysteinate ligands. Structure and energies have been obtained by DFT calculations.

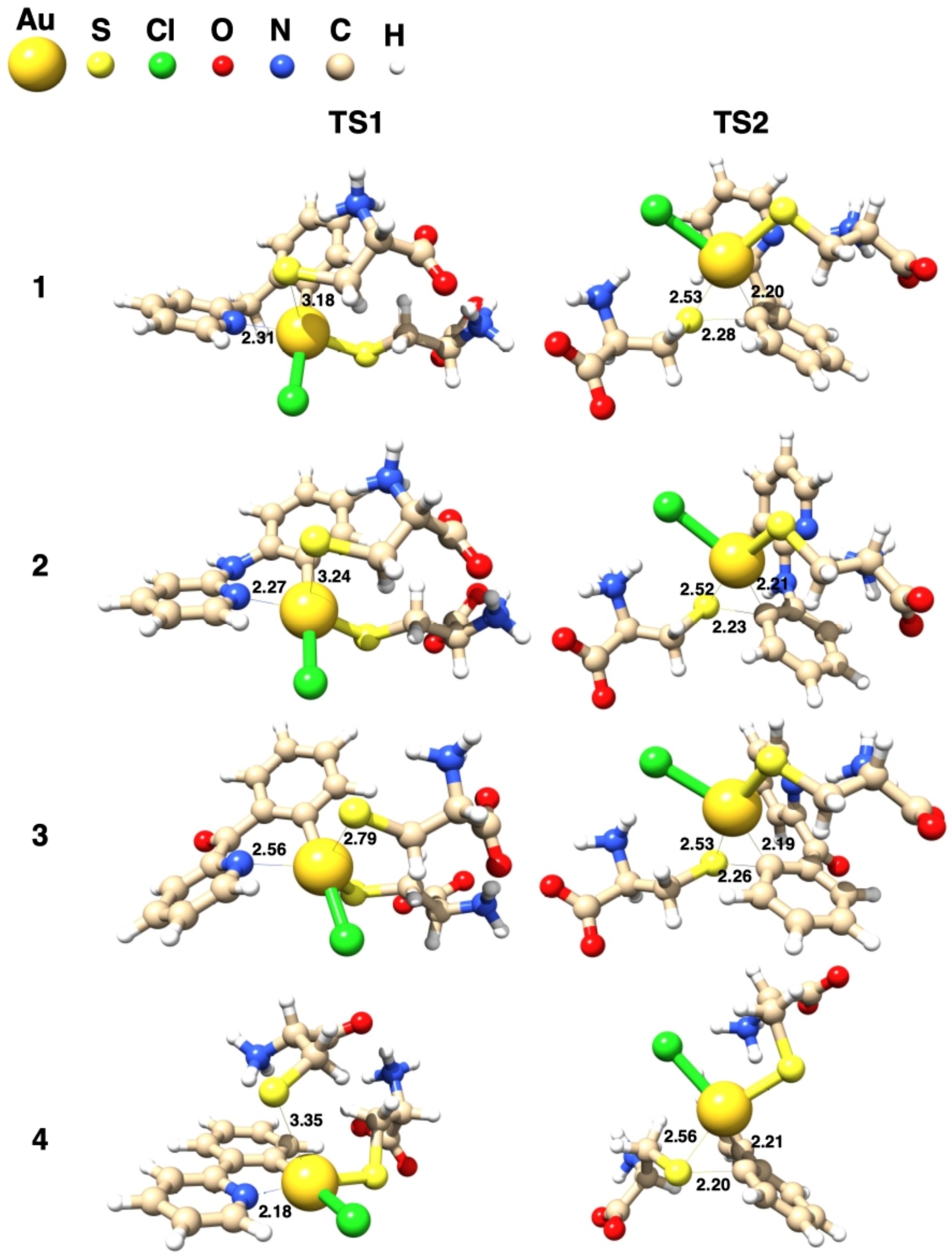

Figure S10. First and second transition states of the Au(III) complexes **1-4**.

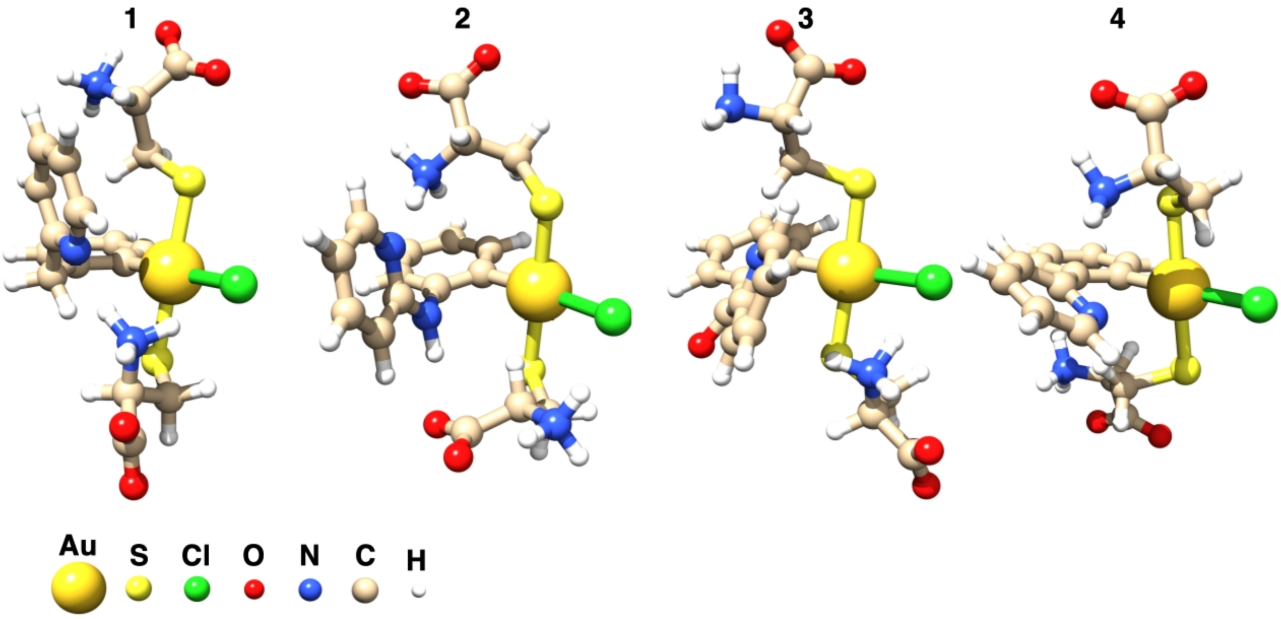

Figure S11. Intermediates of the Au(III) complexes **1**-**4**.

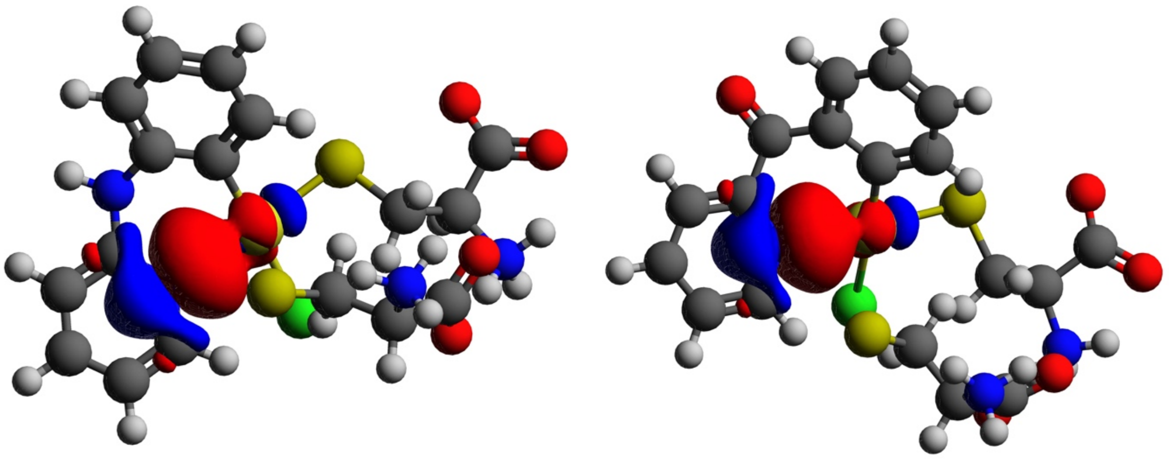

Figure S12. Pyridine N lone pair directed toward the Au atom obtained by the Natural Bond Orbital analysis of the reagent **R** of compounds **2** (left) and **3** (right). The occupancy of both such orbitals is 1.69 electrons. The N-Au distance is 2.165 Å (left) and 2.164 Å (right). Donor-acceptor interactions between the N lone pair and vacant orbitals at gold are found at the second order perturbation theory. The strength of the N-Au interaction, as deduced from the sum of the associated interaction energies ΔE_{int}, is similar in both complexes: ~42 kcal/mol for **2** and ~41 kcal/mol for **3**.

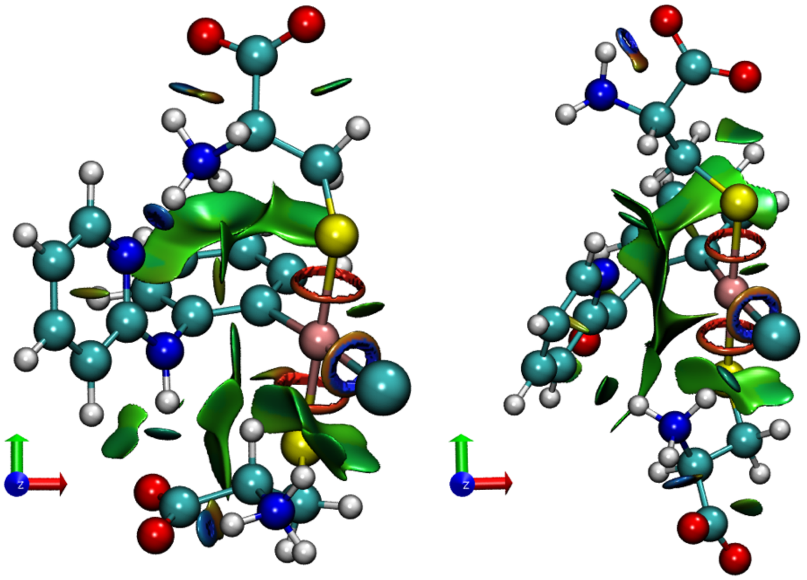

Figure S13. Non-Covalent Interaction plots of the intermediates of compounds **2** (left) and **3** (right).

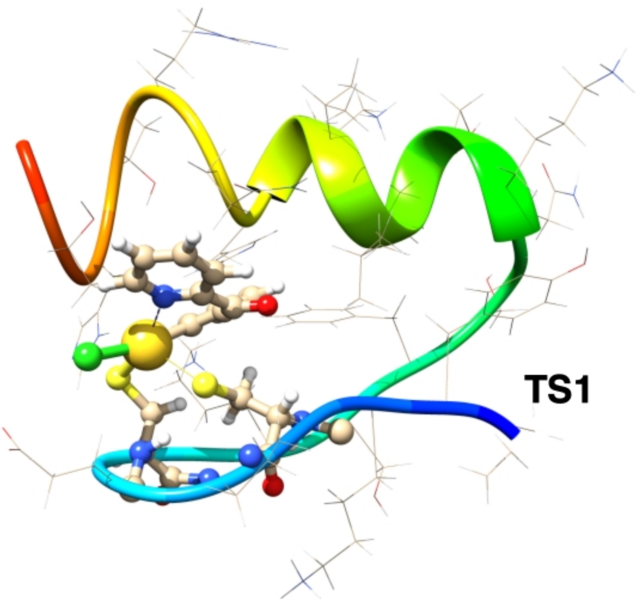

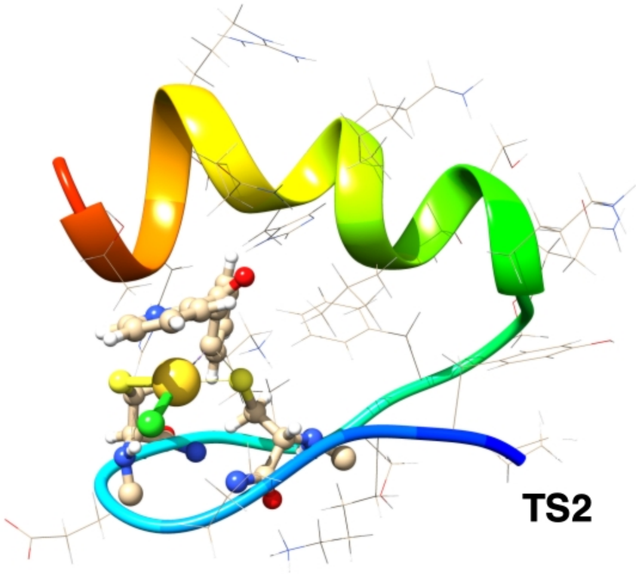

Figure S14. Structures of transitions states, **TS1** and **TS2**, of the adduct of **3** with the ZF (**3-ZF**), obtained by QM/MM calculations. The atoms in the lower MM layer are represented in wires. The protein backbone is highlighted in unstructured tube and alpha helix styles.

Table S1. List of the different adducts detected by LC-MS after 10 min and 24 h incubation of the Zn-ZF and the organometallic Au(III) complexes **1-4**. The focus of the MS spectra has been made between *m/z* = 490 and 595 to allow the visualisation of all adducts at one (or maximum two) charge states. The oxidation state of Au is 3+ unless otherwise specified.

Complex	Time	Adduct	Molecular Formula	*m/z* experimental	*m/z* theoretical	Charge State
**1**	10 min	Apo-ZF+2AuC^{CH2}N	$C_{151}H_{218}N_{41}O_{39}S_2Au_2$	528.1343	528.0781	7+
		Apo-ZF+3AuC^{CH2}N	$C_{163}H_{226}N_{42}O_{39}S_2Au_3$	579.9992	579.9453	7+
	24 h	Apo-ZF+AuC^{CH2}N+C^{CH2}N	$C_{151}H_{219}N_{41}O_{39}S_2Au$	499.9905<br>583.3246	499.9453<br>583.2656	7+<br>6+
		Apo-ZF+2AuC ^{CH2}N	$C_{151}H_{218}N_{41}O_{39}S_2Au_2$	528.1343	528.0781	7+
**2**	10 min	Apo-ZF+2AuC^{NH}N	$C_{149}H_{216}N_{43}O_{39}S_2Au_2$	528.2747	528.2266	7+
		Apo-ZF+AuC^{NH}N	$C_{138}H_{209}N_{41}O_{39}S_2Au$	555.4741	555.4219	6+
		Apo-ZF+3AuC^{NH}N	$C_{160}H_{226}N_{45}O_{39}S_2Au_3$	580.2830	580.2266	7+
	24 h	Apo-ZF+AuC^{NH}N+C^{NH}N	$C_{149}H_{217}N_{43}O_{39}S_2Au$	500.2735<br>583.6514	500.2266<br>583.6016	7+<br>6+
		Apo-ZF+2AuC^{NH}N	$C_{149}H_{216}N_{43}O_{39}S_2Au_2$	528.2747	528.2266	7+
		Apo-ZF+AuC^{NH}N	$C_{138}H_{209}N_{41}O_{39}S_2Au$	555.4741	555.4219	6+
**3**	10 min	Apo-ZF+AuC^{CO}N+C^{CO}N	$C_{151}H_{214}N_{41}O_{41}S_2Au$	503.9332<br>587.7606	503.9375<br>587.7578	7+<br>6+
		Apo-ZF+2AuC^{CO}N	$C_{151}H_{212}N_{41}O_{41}S_2Au_2$	531.7865	531.7891	7+
		Apo-ZF+AuC^{CO}N	$C_{139}H_{207}N_{40}O_{40}S_2Au$	557.4177	557.4141	6+
	24 h	Apo-ZF+AuC^{CO}N+C^{CO}N	$C_{151}H_{215}N_{41}O_{41}S_2Au$	503.9332<br>587.7606	503.9375<br>587.7578	7+<br>6+
		Apo-ZF+2C^{CO}N	$C_{151}H_{216}N_{41}O_{41}S_2$	555.2665	555.2656	6+
		Apo-ZF+3C^{CO}N	$C_{163}H_{220}N_{42}O_{42}S_2$	584.9181	584.9375	6+
**4**	10 min	Apo-ZF	$C_{127}H_{202}N_{39}O_{39}S_2$	494.7648<br>593.5070	494.7500<br>593.5000	6+<br>5+
		Apo-ZF+Au	$C_{127}H_{201}N_{39}O_{39}S_2Au$	527.4226	527.4063	6+
		Apo-ZF+AuC^N	$C_{138}H_{211}N_{40}O_{39}S_2Au$	552.9266	552.9219	6+
	24 h	Apo-ZF	$C_{127}H_{202}N_{39}O_{39}S_2$	494.7648<br>593.5071	494.7500<br>593.5000	6+<br>5+
		Apo-ZF+Au	$C_{127}H_{201}N_{39}O_{39}S_2Au$	527.4226	527.4063	6+
		Apo-ZF+AuC^N	$C_{138}H_{211}N_{40}O_{39}S_2Au$	552.9369	552.9219	6+

Table S2. Standard Gibbs formation free energy in water solution, ΔG° in kJ/mol, of the adducts of compounds **1-4** with the Cys ligand, obtained by DFT calculations and using Eq. 1.

**1 / E**	-82.5	**1 / Z**	-32.6
**2 / E**	-90.1	**2 / Z**	-37.2
**3 / E**	-90.1	**3 / Z**	-37.3
**4 / E**	-75.7	**4 / Z**	-23.8

$$\Delta G° = G°[Au(Ligand)Cys] + G°[Cl^-] - G°[Au(Ligand)Cl] - G°[Cys^-] \qquad \text{Eq. 1}$$

Cartesian coordinates (Å) of the compounds involved in the Reductive Elimination pathway, obtained by DFT and QM/MM calculations

1 R

N	2.504549	1.087774	-0.684650
C	3.762805	0.622451	-0.802700
C	4.823412	1.512924	-0.949066
C	4.574017	2.880073	-0.966048
C	3.265910	3.336828	-0.833170
C	2.249133	2.405910	-0.694925
C	3.940477	-0.866827	-0.748022
C	3.393868	-1.445039	0.534696
C	4.249049	-2.087803	1.436224
C	3.771492	-2.592179	2.642125
C	2.426282	-2.448095	2.967600
C	1.555429	-1.813481	2.080594
C	2.039993	-1.318621	0.872030
H	5.393716	3.582572	-1.077368
H	4.451112	-3.090619	3.326988
H	3.030074	4.394362	-0.834051
H	5.831397	1.124475	-1.046642
H	1.202521	2.680374	-0.587982
H	0.504079	-1.706694	2.328693
H	2.044768	-2.828729	3.911058
H	5.300963	-2.190340	1.178173
H	3.419769	-1.307690	-1.610218
H	4.999541	-1.111165	-0.856021
Au	0.868621	-0.313174	-0.467511
Cl	-0.297176	0.908979	-2.364177
S	-0.811274	-1.940844	-0.248720
C	-2.261263	-0.838569	-0.295819
C	-3.022193	-0.889725	-1.611212
N	-3.578486	0.443087	-2.002858
C	-4.203790	-1.895980	-1.601652
O	-5.256044	-1.492120	-2.170469
O	-3.967844	-2.994330	-1.056999
H	-3.707554	1.102152	-1.184595
H	-2.915149	-1.135759	0.527297
H	-1.901136	0.174486	-0.080269
H	-2.335483	-1.178952	-2.412939
H	-2.969455	0.913343	-2.670244
H	-4.496316	0.191346	-2.416856
S	-0.381480	1.944650	1.604463
C	-1.735984	1.208244	2.571325
C	-3.096965	1.901958	2.425305
H	-1.870135	0.144518	2.319834
H	-1.473312	1.237707	3.638144
N	-4.009564	1.386291	3.516436
H	-2.993629	2.982170	2.543632
C	-3.832368	1.523670	1.118273
H	-4.533822	0.629556	3.023082
H	-3.511647	1.029362	4.330552
H	-4.678443	2.084747	3.834078

O	-4.625288	0.549350	1.212183
O	-3.536851	2.176034	0.086818

1 I

N	-0.685449	-1.644915	1.837107
C	0.115818	-0.678798	2.315698
C	1.413651	-0.956569	2.755975
C	1.889416	-2.259854	2.695262
C	1.062331	-3.257279	2.183350
C	-0.212672	-2.897885	1.769434
C	-0.449268	0.716394	2.386183
C	0.398771	1.760033	1.706680
C	1.116272	2.689011	2.471611
C	1.896420	3.675762	1.875571
C	1.961631	3.758558	0.488212
C	1.262530	2.839688	-0.295649
C	0.504001	1.839481	0.309947
H	2.890335	-2.497640	3.045164
H	2.442158	4.382375	2.493848
H	1.394327	-4.286974	2.110651
H	2.033189	-0.150990	3.144144
H	-0.895834	-3.640835	1.360481
H	1.330905	2.894336	-1.379182
H	2.557138	4.530282	0.008378
H	1.046585	2.632368	3.556534
H	-1.448509	0.707801	1.933064
H	-0.575664	0.992121	3.440205
Au	-0.404720	0.468127	-0.893426
Cl	-1.486425	-1.223909	-2.420784
S	-2.388513	1.806937	-0.568640
C	-3.833405	0.698860	-0.704742
C	-4.050182	-0.271154	0.434975
N	-3.146790	-1.457202	0.364760
C	-5.513134	-0.828795	0.480978
O	-5.592584	-2.074913	0.669161
O	-6.418872	0.016354	0.356284
H	-2.782329	-1.596759	-0.587594
H	-4.704364	1.360566	-0.713045
H	-3.815376	0.167223	-1.662546
H	-3.854400	0.229219	1.390925
H	-2.318644	-1.434806	1.004407
H	-3.806467	-2.233252	0.596131
S	1.638261	-0.732875	-1.322021
C	2.948167	0.018169	-0.296166
C	4.085733	-0.959605	-0.040337
H	3.337092	0.929003	-0.767719
H	2.510205	0.308845	0.664413
N	5.029836	-0.363405	0.974697
H	3.692245	-1.895977	0.365089
C	4.972625	-1.242197	-1.291325
H	5.899719	-0.267027	0.372229
H	4.730007	0.537766	1.343055
H	5.219882	-0.978081	1.762450
O	6.156330	-0.800323	-1.199449

O	4.426191	-1.847919	-2.226803

1 P

N	2.678427	-1.582443	-1.048798
C	3.451707	-2.083240	-0.065493
C	4.838011	-1.925479	-0.072945
C	5.442639	-1.236538	-1.119089
C	4.644246	-0.721248	-2.135256
C	3.271092	-0.923863	-2.053427
C	2.745019	-2.799867	1.054716
C	1.756066	-1.897755	1.751188
C	2.253028	-0.827150	2.510282
C	1.409388	0.091897	3.124535
C	0.028508	-0.045946	2.981945
C	-0.488145	-1.122561	2.271989
C	0.360974	-2.055497	1.654358
H	6.519742	-1.101757	-1.137976
H	1.824626	0.909332	3.707551
H	5.068749	-0.175209	-2.970201
H	5.428847	-2.337681	0.739453
H	2.607861	-0.536035	-2.826006
H	-1.566910	-1.246026	2.207682
H	-0.650299	0.667795	3.440755
H	3.333289	-0.731606	2.621025
H	2.235064	-3.677707	0.647342
H	3.487571	-3.164857	1.770859
Au	-2.204609	1.056536	0.116173
Cl	-4.018441	-0.085298	1.166122
S	-0.329368	-3.510509	0.876427
C	-1.629147	-2.818770	-0.203190
C	-1.142938	-2.357999	-1.554528
N	-0.145460	-1.255265	-1.452089
C	-2.300587	-1.832755	-2.466819
O	-1.979683	-0.847070	-3.185766
O	-3.374316	-2.457927	-2.392846
H	-0.433972	-0.582237	-0.730649
H	-2.352321	-3.619884	-0.361274
H	-2.161807	-2.017129	0.322938
H	-0.637151	-3.179762	-2.073272
H	0.850352	-1.533211	-1.273098
H	-0.279028	-0.742352	-2.344133
S	-0.482423	2.192256	-0.931516
C	0.927324	1.829917	0.182399
C	2.250001	2.355922	-0.361087
H	0.745489	2.264446	1.173155
H	1.014088	0.742696	0.327210
N	3.327439	1.921347	0.597470
H	2.461641	1.941042	-1.349688
C	2.343047	3.915884	-0.364004
H	3.469933	2.827231	1.127495
H	3.052481	1.149316	1.209165
H	4.197487	1.670880	0.132320
O	2.933128	4.382861	0.656221
O	1.829160	4.508796	-1.323918

1 TS1

N	3.053386	-0.092226	-0.410639
C	3.701343	-0.866178	0.474720
C	5.057319	-0.663904	0.729118
C	5.736124	0.339446	0.047410
C	5.047218	1.123615	-0.873526
C	3.695915	0.877791	-1.071429
C	2.896545	-1.935061	1.160752
C	1.742068	-1.375695	1.954774
C	1.716824	-1.496970	3.350783
C	0.666829	-0.977271	4.100301
C	-0.381178	-0.319464	3.460491
C	-0.374254	-0.189388	2.072000
C	0.685559	-0.704696	1.336182
H	6.792210	0.507865	0.233956
H	0.665950	-1.088305	5.180512
H	5.540777	1.913990	-1.427390
H	5.564424	-1.293492	1.452982
H	3.085283	1.455004	-1.760052
H	-1.195323	0.325468	1.577315
H	-1.209984	0.090132	4.032943
H	2.536511	-2.014858	3.844572
H	2.513783	-2.618522	0.389458
H	3.551220	-2.521655	1.809800
Au	0.784328	-0.427702	-0.680718
Cl	0.995040	0.036679	-3.213853
S	-1.265810	-1.815285	-0.918028
C	-2.602905	-0.489658	-0.817094
C	-4.000536	-1.032599	-1.079829
N	-4.971938	0.074089	-0.782558
C	-4.384933	-2.212226	-0.140936
O	-5.134428	-1.894795	0.824262
O	-3.899994	-3.321231	-0.437044
H	-4.540548	1.039170	-0.680242
H	-2.582307	-0.056606	0.187137
H	-2.370092	0.290277	-1.546139
H	-4.108503	-1.331831	-2.124777
H	-5.721190	0.114220	-1.467871
H	-5.371466	-0.252404	0.126101
S	0.962889	2.676499	-0.038932
C	-0.853447	2.798814	-0.518165
C	-1.810230	3.310547	0.586691
H	-0.970587	3.444966	-1.389847
H	-1.169464	1.799296	-0.824956
N	-1.207472	3.170682	1.943602
H	-2.044681	4.363726	0.428955
C	-3.088179	2.446800	0.568954
H	-1.791671	2.452628	2.412644
H	-0.211709	2.864928	1.816031
H	-1.225315	4.029602	2.485912
O	-3.301244	1.731235	1.580392
O	-3.737646	2.484873	-0.509292

1 TS2

N	-1.948364	2.056935	1.087676
C	-0.958410	1.837675	1.969578
C	0.073896	2.759792	2.159478
C	0.084570	3.934820	1.416401
C	-0.939362	4.159189	0.501776
C	-1.926677	3.189248	0.375065
C	-1.017548	0.560354	2.763263
C	-1.101062	-0.735787	1.999637
C	-2.055028	-1.688640	2.366571
C	-2.063676	-2.977668	1.836147
C	-1.084590	-3.340829	0.916441
C	-0.132933	-2.411219	0.502723
C	-0.166131	-1.103098	1.006867
H	0.881615	4.660733	1.546612
H	-2.821819	-3.688939	2.147541
H	-0.973043	5.058858	-0.102822
H	0.860742	2.543608	2.876814
H	-2.738935	3.318469	-0.339132
H	0.612043	-2.695232	-0.234931
H	-1.059604	-4.343710	0.499708
H	-2.796248	-1.405527	3.112141
H	-0.129394	0.519413	3.409926
H	-1.879405	0.614289	3.439017
Au	0.370387	0.356696	-0.501348
Cl	2.039409	1.929425	-1.975208
S	1.821729	-0.135785	1.341501
C	2.944784	-1.353293	0.557490
C	4.355730	-0.817165	0.463145
N	4.459368	0.402199	-0.403556
C	5.313364	-1.889641	-0.165553
O	5.943926	-1.485959	-1.181456
O	5.326166	-2.990024	0.409037
H	3.584035	0.714616	-0.865636
H	2.972684	-2.269360	1.151616
H	2.566161	-1.603784	-0.438953
H	4.725588	-0.550875	1.456404
H	4.834003	1.206897	0.091716
H	5.159014	0.071634	-1.116754
S	-1.405883	0.459432	-2.104536
C	-2.548811	-0.878695	-1.587010
C	-3.963501	-0.437366	-1.270505
H	-2.631653	-1.612422	-2.392712
H	-2.124717	-1.401113	-0.721914
N	-4.029398	0.398272	-0.030444
H	-4.377556	0.142478	-2.099003
C	-4.852772	-1.693603	-0.995183
H	-4.442818	-0.285505	0.647924
H	-3.137620	0.821210	0.319319
H	-4.696250	1.159718	-0.127923
O	-5.243550	-1.809908	0.201446
O	-5.040690	-2.441520	-1.971412

2 R

N	2.581309	0.654680	-0.818133
C	3.770739	0.058114	-0.603624
C	4.961881	0.662127	-1.047105
C	4.909895	1.905103	-1.638797
C	3.676276	2.551361	-1.783336
C	2.542103	1.889035	-1.371334
N	3.818199	-1.148698	0.047509
H	5.904702	0.150615	-0.886800
H	5.826132	2.384140	-1.968325
H	3.596201	3.539325	-2.219127
H	1.549532	2.307912	-1.494584
H	4.759793	-1.498681	0.154168
C	2.948131	-1.557935	1.078725
C	3.522459	-2.224559	2.171663
C	2.743913	-2.610751	3.252513
C	1.382288	-2.318689	3.268303
C	0.800235	-1.686610	2.171830
C	1.566305	-1.328492	1.060653
H	4.592842	-2.415965	2.169839
H	3.208779	-3.121395	4.090328
H	0.767870	-2.592688	4.120549
H	-0.266918	-1.489888	2.165935
Au	0.748756	-0.477337	-0.595560
Cl	-0.180294	0.630709	-2.623454
S	-1.133461	-1.891621	-0.450616
C	-2.459543	-0.639321	-0.411468
C	-3.659554	-1.054750	-1.249347
N	-4.604334	0.101148	-1.396734
C	-4.446861	-2.204941	-0.568112
O	-5.632105	-1.932124	-0.238130
O	-3.800982	-3.260432	-0.394942
H	-4.215042	1.027707	-1.044010
H	-2.772028	-0.457265	0.622976
H	-2.049922	0.291944	-0.805955
H	-3.325938	-1.360058	-2.245184
H	-4.900932	0.221434	-2.361049
H	-5.427025	-0.220864	-0.845041
S	0.535695	2.682335	2.212747
C	-0.831923	2.184418	1.113092
C	-2.125527	2.974177	1.364882
H	-0.587042	2.309556	0.051194
H	-1.054956	1.114626	1.252410
N	-2.361753	3.001402	2.836934
H	-2.017828	4.002292	1.014480
C	-3.315194	2.252407	0.714967
H	-2.987307	2.200391	3.020836
H	-1.421311	2.915177	3.289337
H	-2.824187	3.847561	3.157860
O	-4.029124	1.553468	1.472626
O	-3.390986	2.366131	-0.541309

2 I

N	-1.165970	2.628126	-0.262818
C	0.121745	2.563240	0.133826

C	1.093155	3.469435	-0.348316
C	0.706431	4.435029	-1.254444
C	-0.628788	4.507890	-1.671336
C	-1.511632	3.586346	-1.139266
N	0.479193	1.569860	1.002896
H	2.112871	3.400043	0.016984
H	1.439896	5.141982	-1.632461
H	-0.967368	5.255693	-2.378785
H	-2.563775	3.597646	-1.422950
H	1.473605	1.491428	1.251427
C	-0.464646	0.789255	1.717580
C	-1.231449	1.376153	2.731574
C	-2.139579	0.620115	3.467591
C	-2.282999	-0.741636	3.205158
C	-1.532396	-1.338070	2.187910
C	-0.646290	-0.571541	1.430971
H	-1.101092	2.436294	2.932427
H	-2.726453	1.090975	4.249987
H	-2.978785	-1.343192	3.782365
H	-1.653482	-2.397845	1.978124
Au	0.450316	-1.382892	-0.069001
Cl	1.908288	-2.200577	-1.951407
S	2.210359	-1.383048	1.586886
C	3.685943	-1.041102	0.584343
C	3.566006	0.178970	-0.322632
N	4.441180	0.015761	-1.533871
C	3.926684	1.552853	0.293251
O	4.719294	2.254036	-0.386118
O	3.348502	1.838145	1.368316
H	5.243760	-0.586865	-1.357489
H	4.508161	-0.909277	1.296568
H	3.906160	-1.920645	-0.027167
H	2.543091	0.248959	-0.711321
H	3.927997	-0.369734	-2.327664
H	4.795559	0.985262	-1.690773
S	-1.324006	-1.409357	-1.687251
C	-2.995278	-1.328550	-0.946692
C	-3.676150	0.021469	-0.926383
H	-3.617805	-1.963908	-1.582860
H	-2.999447	-1.785916	0.049209
N	-3.318908	0.854997	0.257457
H	-3.377612	0.598471	-1.809781
C	-5.242263	-0.065298	-0.945235
H	-4.211989	1.363617	0.431082
H	-3.142038	0.272566	1.078057
H	-2.495146	1.498026	0.117179
O	-5.813576	0.800136	-0.225275
O	-5.735853	-0.934242	-1.687464

2 P

N	3.059848	-0.729268	-0.607313
C	3.882462	-1.104466	0.388483
C	5.229173	-1.449359	0.160892
C	5.723363	-1.394303	-1.125641

C	4.878452	-1.006892	-2.171816
C	3.570150	-0.696133	-1.855414
N	3.393778	-1.147889	1.677603
H	5.856583	-1.748360	0.994559
H	6.760475	-1.651753	-1.317939
H	5.226587	-0.951423	-3.196222
H	2.872828	-0.392496	-2.635565
H	4.103409	-1.124970	2.395274
C	2.122241	-0.667897	2.072013
C	2.042273	0.503109	2.835094
C	0.815450	0.984952	3.284127
C	-0.348439	0.292358	2.963860
C	-0.278888	-0.894691	2.238166
C	0.948061	-1.396399	1.787355
H	2.963320	1.029629	3.075745
H	0.773437	1.898116	3.868976
H	-1.318428	0.654709	3.293200
H	-1.196146	-1.447275	2.052363
Au	-2.559471	0.432280	0.055614
Cl	-3.816916	-1.392205	0.944261
S	1.094754	-2.995830	1.020571
C	-0.362143	-3.047605	-0.080762
C	-0.127988	-2.388948	-1.418617
N	0.255906	-0.957722	-1.258444
C	-1.374787	-2.424324	-2.358177
O	-1.508777	-1.390131	-3.066767
O	-2.057547	-3.464216	-2.309269
H	-0.402562	-0.493219	-0.616320
H	-0.578746	-4.102648	-0.250760
H	-1.241041	-2.626300	0.421212
H	0.714514	-2.868797	-1.930604
H	1.237738	-0.814989	-0.924651
H	0.048265	-0.533793	-2.176678
S	-1.360572	2.173852	-0.884631
C	0.196900	2.193839	0.088725
C	1.358437	2.770331	-0.713633
H	0.061010	2.767560	1.013692
H	0.452345	1.171972	0.400089
N	2.634364	2.496585	0.037899
H	1.421098	2.295937	-1.696507
C	1.295636	4.326499	-0.833563
H	2.823116	3.457942	0.437973
H	2.543995	1.777686	0.759623
H	3.402920	2.234858	-0.575730
O	0.482743	4.790142	-1.647609
O	2.081680	4.927240	-0.042295

2 TS1

N	-3.016942	-0.215782	-0.226209
C	-3.733600	0.874479	0.085154
C	-5.139696	0.851752	0.017082
C	-5.773171	-0.324349	-0.327240
C	-5.015534	-1.472215	-0.588339
C	-3.640430	-1.368300	-0.532064

N	-3.086135	2.024045	0.476044
H	-5.702175	1.747672	0.258305
H	-6.857215	-0.357160	-0.373818
H	-5.481354	-2.417395	-0.839402
H	-2.974208	-2.200515	-0.739533
H	-3.722893	2.767261	0.724440
C	-1.847294	2.107481	1.142380
C	-1.743476	3.048939	2.181124
C	-0.569302	3.187860	2.905430
C	0.527153	2.378188	2.615456
C	0.441812	1.462439	1.569602
C	-0.729555	1.328389	0.829345
H	-2.610159	3.660396	2.422113
H	-0.518233	3.920258	3.705221
H	1.450245	2.465422	3.181571
H	1.307378	0.848844	1.328633
Au	-0.813384	0.001083	-0.703413
Cl	-0.945728	-1.744062	-2.589279
S	1.179967	1.072968	-1.725280
C	2.534562	-0.077322	-1.095658
C	3.916440	0.279515	-1.626570
N	4.923850	-0.526530	-0.856468
C	4.298551	1.772426	-1.403459
O	5.092354	1.977352	-0.443347
O	3.774653	2.588720	-2.185795
H	4.523689	-1.286366	-0.232355
H	2.554866	-0.000239	-0.005895
H	2.275002	-1.100692	-1.379594
H	3.997772	0.031576	-2.687168
H	5.634178	-0.928023	-1.461774
H	5.362521	0.219403	-0.269298
S	-0.864716	-2.358348	1.518609
C	0.943956	-2.668908	1.103757
C	1.944856	-2.395716	2.252862
H	1.087652	-3.700450	0.777100
H	1.195792	-2.033114	0.251033
N	1.370210	-1.463018	3.264889
H	2.202789	-3.326539	2.759575
C	3.197417	-1.712922	1.668946
H	1.925114	-0.592700	3.162060
H	0.354192	-1.336558	3.028957
H	1.451049	-1.800100	4.219808
O	3.429143	-0.535215	2.044694
O	3.806560	-2.382460	0.793128

2 TS2

N	-1.915915	2.052957	0.680443
C	-1.054018	2.265757	1.688639
C	-0.424071	3.509200	1.889947
C	-0.706451	4.540590	1.015200
C	-1.597017	4.327314	-0.042814
C	-2.162953	3.068967	-0.160170
N	-0.817927	1.215807	2.546291
H	0.275486	3.637248	2.710695

H	-0.228223	5.506779	1.147561
H	-1.838292	5.111705	-0.750634
H	-2.854746	2.841343	-0.970832
H	-0.156910	1.392108	3.290217
C	-1.014023	-0.148609	2.202908
C	-1.955434	-0.904914	2.895076
C	-2.085958	-2.275678	2.672142
C	-1.246521	-2.898809	1.751942
C	-0.292274	-2.167250	1.048512
C	-0.185909	-0.780690	1.243041
H	-2.580366	-0.394653	3.623189
H	-2.831524	-2.848111	3.213470
H	-1.332624	-3.965660	1.565959
H	0.359897	-2.670039	0.339157
Au	0.369193	0.319538	-0.541095
Cl	1.995354	1.820383	-2.127217
S	1.803761	0.132226	1.364822
C	2.865019	-1.235909	0.761366
C	4.288668	-0.777539	0.535652
N	4.400188	0.273223	-0.528113
C	5.172213	-1.984184	0.059112
O	5.720667	-1.809706	-1.063771
O	5.208005	-2.952375	0.835040
H	3.512933	0.603222	-0.951704
H	2.885473	-2.037774	1.502739
H	2.440293	-1.636627	-0.165029
H	4.705461	-0.368217	1.458897
H	4.897706	1.100326	-0.208259
H	4.995073	-0.231073	-1.235042
S	-1.218087	0.034917	-2.302844
C	-2.265235	-1.373836	-1.788453
C	-3.650721	-1.010224	-1.291670
H	-2.406402	-2.031582	-2.649825
H	-1.741615	-1.959226	-1.021337
N	-3.578925	-0.236586	-0.014473
H	-4.173887	-0.399703	-2.031440
C	-4.444606	-2.317484	-0.980687
H	-3.638910	-0.978365	0.712258
H	-2.765918	0.406739	0.117760
H	-4.413107	0.333870	0.107602
O	-4.578943	-2.570773	0.247950
O	-4.802730	-2.971475	-1.978639

3 R

N	2.429663	0.839888	-0.671929
C	3.648388	0.281051	-0.554870
C	4.799841	1.016951	-0.824819
C	4.687219	2.358596	-1.167203
C	3.423210	2.932385	-1.232782
C	2.310243	2.136726	-0.993805
C	3.818799	-1.116573	-0.029741
H	5.758071	0.518410	-0.738903
H	5.574260	2.949549	-1.369299
H	3.286272	3.979761	-1.474156

H	1.298518	2.517810	-1.074335
O	4.862158	-1.707595	-0.282764
C	2.838764	-1.647263	0.949680
C	3.391105	-2.435073	1.976833
C	2.610364	-2.875020	3.033023
C	1.257359	-2.541040	3.072730
C	0.680854	-1.808805	2.035681
C	1.464683	-1.365619	0.971252
H	4.452908	-2.658608	1.928646
H	3.054047	-3.465287	3.828558
H	0.638491	-2.862446	3.905763
H	-0.383284	-1.597122	2.049892
Au	0.652988	-0.393776	-0.621080
Cl	-0.226006	0.847212	-2.591575
S	-1.186605	-1.872104	-0.636877
C	-2.536652	-0.651911	-0.494391
C	-3.794809	-1.116785	-1.214720
N	-4.807720	-0.011496	-1.209928
C	-4.440943	-2.329801	-0.493992
O	-5.565032	-2.111489	0.032025
O	-3.754779	-3.373787	-0.489300
H	-4.421693	0.933655	-0.904467
H	-2.766443	-0.463111	0.561140
H	-2.189104	0.283818	-0.938127
H	-3.557113	-1.371762	-2.251258
H	-5.250779	0.093889	-2.118239
H	-5.521895	-0.377264	-0.545927
S	0.487157	2.724463	2.003077
C	-0.947101	2.179461	1.019617
C	-2.235008	2.950935	1.343355
H	-0.782441	2.278935	-0.060877
H	-1.144025	1.111118	1.202875
N	-2.371251	2.999122	2.827083
H	-2.173207	3.974246	0.968993
C	-3.444584	2.187399	0.785289
H	-2.951498	2.179847	3.068578
H	-1.399918	2.952558	3.215004
H	-2.841159	3.834870	3.164284
O	-4.068834	1.460192	1.593735
O	-3.620767	2.298783	-0.461030

3 I

N	0.467675	0.243808	2.002094
C	-0.518897	1.144626	2.117750
C	-1.604231	0.978564	2.984496
C	-1.675816	-0.180412	3.748761
C	-0.662203	-1.129498	3.620812
C	0.383855	-0.867797	2.738432
C	-0.393761	2.427751	1.355385
H	-2.358636	1.756407	3.041965
H	-2.506360	-0.344727	4.428540
H	-0.678448	-2.050532	4.193342
H	1.194070	-1.585376	2.615766
O	-1.039594	3.401191	1.734624

C	0.594763	2.573056	0.245759
C	1.256633	3.814469	0.213872
C	2.185221	4.119838	-0.768259
C	2.447697	3.188898	-1.770747
C	1.783779	1.963927	-1.775439
C	0.868989	1.640543	-0.769590
H	1.018881	4.531110	0.995131
H	2.695679	5.077975	-0.757714
H	3.166627	3.410293	-2.554794
H	1.993010	1.244397	-2.561768
Au	-0.094755	-0.147319	-0.989667
Cl	-1.201919	-2.348214	-1.454536
S	-2.105619	1.208460	-1.119245
C	-3.568591	0.117575	-1.196803
C	-4.112547	-0.338654	0.137135
N	-3.209810	-1.315211	0.824963
C	-5.501178	-1.049454	0.023211
O	-5.595297	-2.115291	0.696433
O	-6.346006	-0.477362	-0.687198
H	-2.564680	-1.777453	0.162501
H	-4.361755	0.699656	-1.672552
H	-3.362736	-0.740298	-1.845089
H	-4.223676	0.519870	0.808267
H	-2.658529	-0.912855	1.581481
H	-3.908246	-2.015593	1.178150
S	1.983655	-1.364499	-0.961078
C	3.108586	-0.390118	0.096327
C	4.014006	-1.302022	0.905796
H	3.708109	0.299337	-0.510117
H	2.478520	0.197394	0.773162
N	4.662807	-0.549523	2.040175
H	3.426127	-2.106006	1.359757
C	5.196779	-1.943649	0.122317
H	5.645560	-0.937149	1.979495
H	4.692434	0.455634	1.878322
H	4.237382	-0.709392	2.950275
O	6.312708	-1.874003	0.715834
O	4.914835	-2.461731	-0.971455

3 P

N	3.073481	-0.342269	-0.846130
C	3.983774	-0.730127	0.068068
C	5.292766	-1.071817	-0.273376
C	5.680447	-1.017194	-1.606111
C	4.750983	-0.602253	-2.552508
C	3.466212	-0.277344	-2.123028
C	3.621037	-0.698509	1.520741
C	2.217657	-0.427104	1.962299
C	2.029722	0.691882	2.788598
C	0.767927	1.029214	3.263353
C	-0.319139	0.209997	2.957245
C	-0.135951	-0.945772	2.205807
C	1.126710	-1.272708	1.690763
H	6.690055	-1.286028	-1.899433

H	0.634994	1.916578	3.873900
H	5.005627	-0.532226	-3.603992
H	5.977818	-1.364959	0.514039
H	2.714473	0.047270	-2.840925
H	-0.984217	-1.604992	2.039534
H	-1.313256	0.451301	3.324021
H	2.895444	1.299541	3.041591
O	4.507464	-0.786086	2.362449
Au	-2.691441	0.117252	0.132858
Cl	-3.609199	-1.898831	1.027486
S	1.427218	-2.833793	0.888607
C	-0.020663	-3.000918	-0.213805
C	0.096601	-2.248297	-1.515132
N	0.258969	-0.782133	-1.296748
C	-1.148143	-2.427949	-2.443840
O	-1.442413	-1.394352	-3.103334
O	-1.670426	-3.557018	-2.433782
H	-0.420389	-0.451689	-0.596652
H	-0.106438	-4.063828	-0.441409
H	-0.934855	-2.723525	0.324887
H	0.990972	-2.572372	-2.059940
H	1.223401	-0.503095	-1.014527
H	-0.071382	-0.365312	-2.185610
S	-1.818323	2.039086	-0.818074
C	-0.286413	2.323021	0.155338
C	0.789160	3.044046	-0.647492
H	-0.518109	2.888406	1.066504
H	0.113344	1.356826	0.486716
N	2.081988	3.010670	0.128651
H	0.952841	2.537114	-1.602988
C	0.503224	4.561861	-0.880151
H	2.214244	4.052026	0.297558
H	2.020060	2.494046	1.007541
H	2.863713	2.635366	-0.402978
O	1.305774	5.335120	-0.276823
O	-0.463764	4.837088	-1.606059

3 TS1

N	-2.829004	-0.722642	-0.359435
C	-3.802111	0.198481	-0.312690
C	-5.119339	-0.118680	-0.650242
C	-5.426495	-1.422446	-1.017172
C	-4.410535	-2.372751	-1.043243
C	-3.117143	-1.976228	-0.718809
C	-3.513434	1.588650	0.176115
H	-5.870025	0.661761	-0.604460
H	-6.444988	-1.694845	-1.274694
H	-4.607840	-3.403899	-1.314299
H	-2.271110	-2.659472	-0.742010
O	-4.358608	2.455205	-0.031339
C	-2.333096	1.884720	1.035538
C	-2.575216	2.854640	2.031634
C	-1.599789	3.211415	2.945550
C	-0.338970	2.618112	2.869672

C	-0.065636	1.687778	1.871614
C	-1.055651	1.308875	0.963206
H	-3.564607	3.301282	2.067566
H	-1.816370	3.946984	3.713997
H	0.438871	2.886988	3.579064
H	0.926172	1.248295	1.799813
Au	-0.569093	-0.019258	-0.478094
Cl	-0.047275	-1.721623	-2.236922
S	0.986265	1.579896	-1.373779
C	2.424335	0.545614	-0.946550
C	3.732415	0.996558	-1.581420
N	4.781655	0.007951	-1.163007
C	4.187894	2.385823	-1.065603
O	5.113752	2.357127	-0.207374
O	3.566317	3.363019	-1.529325
H	4.379352	-0.895654	-0.778213
H	2.555386	0.520040	0.142189
H	2.206307	-0.473364	-1.274362
H	3.653929	0.995046	-2.671688
H	5.431192	-0.199793	-1.916155
H	5.291789	0.543864	-0.429598
S	-0.288275	-1.982923	1.503604
C	1.014513	-3.185583	1.064437
C	2.360310	-2.934320	1.761998
H	0.654880	-4.187367	1.320174
H	1.199124	-3.167192	-0.013825
N	2.157226	-2.386358	3.135343
H	2.905578	-3.877678	1.854473
C	3.284939	-1.931764	1.035007
H	2.851599	-1.616215	3.201631
H	1.199677	-1.970736	3.146384
H	2.260348	-3.060497	3.888175
O	3.700203	-0.955686	1.708446
O	3.548818	-2.217931	-0.162755

3 TS2

N	-1.369438	2.173149	0.418117
C	-0.911594	2.028998	1.672467
C	-0.105252	2.978402	2.299299
C	0.285652	4.098808	1.576135
C	-0.181244	4.254124	0.273310
C	-1.019367	3.277588	-0.254038
C	-1.391776	0.825106	2.427453
H	0.215126	2.818675	3.323731
H	0.936874	4.843559	2.022578
H	0.092934	5.116606	-0.324189
H	-1.419734	3.365940	-1.262648
O	-1.887280	1.013092	3.537399
C	-1.277378	-0.538451	1.864418
C	-2.165523	-1.494273	2.393288
C	-2.078825	-2.838357	2.080461
C	-1.034190	-3.270990	1.256538
C	-0.120689	-2.366487	0.736884
C	-0.252535	-0.983987	0.983827

H	-2.932963	-1.126469	3.068743
H	-2.795374	-3.546152	2.483016
H	-0.927512	-4.323998	1.011373
H	0.683271	-2.727331	0.102615
Au	0.362258	0.122177	-0.759713
Cl	2.095326	1.330703	-2.394731
S	1.666864	0.065309	1.256720
C	2.892754	-1.208137	0.764546
C	4.288536	-0.629630	0.724922
N	4.442842	0.458621	-0.295094
C	5.340952	-1.734770	0.352983
O	6.095377	-1.421004	-0.608296
O	5.299514	-2.763434	1.046050
H	3.630024	0.604279	-0.924109
H	2.887111	-2.025926	1.488221
H	2.621825	-1.613752	-0.215544
H	4.551933	-0.209694	1.699072
H	4.672524	1.361126	0.112813
H	5.263973	0.091114	-0.841909
S	-1.275322	-0.247127	-2.468893
C	-2.582169	-1.201380	-1.608199
C	-3.895373	-0.464350	-1.438819
H	-2.803492	-2.108963	-2.173918
H	-2.206538	-1.523503	-0.630264
N	-3.738907	0.797192	-0.647039
H	-4.305884	-0.198776	-2.416106
C	-4.916717	-1.348692	-0.654363
H	-4.321702	0.569708	0.198272
H	-2.767499	1.058834	-0.380184
H	-4.143460	1.603570	-1.115337
O	-5.301607	-0.858374	0.446380
O	-5.209509	-2.432477	-1.189312

4 R

C	-0.994507	1.473546	2.969211
H	-1.955710	1.655479	3.443175
C	0.093713	2.298730	3.241185
C	1.314809	2.054452	2.624363
C	1.458890	0.990071	1.723044
C	0.342567	0.166883	1.446460
C	-0.869978	0.404564	2.079037
H	-0.005276	3.129271	3.932943
H	2.162238	2.698607	2.842045
C	2.715173	0.698423	1.043438
H	-1.735994	-0.222634	1.885819
N	2.688338	-0.386458	0.227066
C	3.767132	-0.749081	-0.478004
C	4.959413	-0.047014	-0.393317
C	5.020282	1.060165	0.449103
C	3.895424	1.433356	1.169390
H	5.814531	-0.367415	-0.976190
H	5.937645	1.632402	0.540827
H	3.926098	2.297353	1.823185
Au	0.740937	-1.271183	0.054532

S	-1.424497	-2.183272	0.067808
C	-2.317671	-0.785268	-0.698250
C	-3.832289	-0.913778	-0.558544
N	-4.402878	0.431603	-0.884156
C	-4.242684	-1.232884	0.906772
O	-4.346732	-0.212095	1.643172
O	-4.384489	-2.438982	1.188173
H	-3.818215	1.016824	-1.549394
H	-2.016572	0.140011	-0.195498
H	-2.035597	-0.695628	-1.751973
H	-4.239943	-1.652887	-1.251387
H	-5.361577	0.364466	-1.215693
H	-4.403987	0.903769	0.037781
Cl	1.491126	-2.939170	-1.616458
H	3.639385	-1.619396	-1.115249
S	1.089358	1.406388	-2.011778
C	0.065962	2.770558	-2.676112
C	-0.852550	3.372199	-1.615661
H	0.713476	3.553484	-3.088002
H	-0.589228	2.429080	-3.481546
N	-0.127425	3.508436	-0.317559
H	-1.185974	4.371698	-1.915720
C	-2.112407	2.532589	-1.300428
H	-0.840519	3.312914	0.407666
H	0.588397	2.737775	-0.334533
H	0.325411	4.406671	-0.173230
O	-2.449137	2.478102	-0.089389
O	-2.696320	2.008916	-2.286623

4 I

C	-1.852519	1.621323	-2.885752
H	-2.398572	1.510763	-3.818202
C	-1.618157	2.886152	-2.347649
C	-0.914202	3.003471	-1.154365
C	-0.428842	1.875341	-0.469190
C	-0.691595	0.600979	-1.012180
C	-1.387368	0.487502	-2.216335
H	-1.983477	3.775713	-2.851294
H	-0.751966	3.989971	-0.728330
C	0.332436	2.068730	0.784333
H	-1.579036	-0.495091	-2.639106
N	0.263982	1.086913	1.705309
C	0.936600	1.235282	2.848936
C	1.701662	2.358214	3.155909
C	1.777231	3.373980	2.206152
C	1.093454	3.227134	1.004850
H	2.222509	2.428716	4.104726
H	2.372874	4.262927	2.390258
H	1.167010	3.990923	0.237499
Au	-0.036748	-1.121662	-0.137397
S	-2.202463	-1.647057	0.761561
C	-3.035554	-0.047840	1.021516
C	-4.023672	0.299212	-0.079851
N	-4.174390	1.783362	-0.281898

C	-5.472033	-0.227480	0.155259
O	-6.376682	0.583838	-0.199608
O	-5.572445	-1.368103	0.637849
H	-3.979636	2.318998	0.562497
H	-3.561181	-0.121648	1.978535
H	-2.262626	0.719567	1.131823
H	-3.668380	-0.091195	-1.038026
H	-3.598464	2.158835	-1.038506
H	-5.206926	1.840378	-0.488220
Cl	0.748727	-3.274070	0.838783
H	0.854135	0.411758	3.556963
S	2.104676	-0.625840	-1.148911
C	3.327649	-1.184415	0.102623
C	4.421352	-0.151453	0.277740
H	2.807037	-1.390613	1.041251
H	3.803896	-2.108026	-0.231541
N	3.861718	1.198264	0.592728
H	5.065981	-0.440774	1.113658
C	5.321860	0.053904	-0.981856
H	4.497211	1.816465	0.026505
H	2.912559	1.259399	0.194783
H	3.841291	1.442888	1.579822
O	5.559374	1.266504	-1.253123
O	5.714270	-0.982625	-1.543396

4 P

N	-3.097496	-1.114632	0.816345
C	-3.411929	-1.166107	-0.494722
C	-4.723154	-1.367553	-0.930400
C	-5.732401	-1.537113	0.010989
C	-5.406696	-1.492541	1.361890
C	-4.077496	-1.276533	1.711128
C	-2.312716	-0.899435	-1.452184
C	-2.496548	0.116199	-2.405089
C	-1.456142	0.539157	-3.226050
C	-0.205489	-0.066634	-3.115181
C	-0.029358	-1.135696	-2.242739
C	-1.072433	-1.569993	-1.413995
H	-6.756781	-1.703314	-0.307609
H	-1.618687	1.342563	-3.937663
H	-6.160520	-1.618774	2.130867
H	-4.937865	-1.401745	-1.994065
H	-3.778874	-1.224658	2.756280
H	0.926261	-1.654976	-2.216366
H	0.624595	0.261426	-3.734313
H	-3.473728	0.591437	-2.475292
Au	2.389213	0.406151	-0.329648
Cl	3.662046	-1.309803	-1.402664
S	-0.895961	-3.094286	-0.502281
C	0.590948	-2.779702	0.513498
C	0.301044	-2.177415	1.865757
N	-0.381171	-0.855877	1.758378
C	1.578268	-1.961505	2.740102
O	1.508804	-0.962391	3.503241

O	2.484035	-2.805916	2.596641
H	0.155944	-0.226876	1.144520
H	1.078094	-3.742323	0.672306
H	1.291014	-2.162711	-0.063550
H	-0.393043	-2.822565	2.417462
H	-1.381520	-0.910490	1.437160
H	-0.268981	-0.439336	2.693394
S	1.233896	2.077469	0.769793
C	-0.316781	2.217432	-0.204624
C	-1.456970	2.788947	0.629855
H	-0.154159	2.843224	-1.090891
H	-0.600099	1.221924	-0.568159
N	-2.721605	2.722627	-0.185819
H	-1.599144	2.201797	1.540898
C	-1.285390	4.305173	0.965714
H	-2.851328	3.754131	-0.399613
H	-2.636823	2.158940	-1.033278
H	-3.519940	2.379943	0.344344
O	-2.028714	5.065838	0.276260
O	-0.448202	4.592124	1.834459

4 TS1

C	1.041903	1.851885	-2.673680
H	2.020623	2.059060	-3.098826
C	-0.023469	2.722911	-2.886225
C	-1.269230	2.432028	-2.342924
C	-1.459376	1.280940	-1.565822
C	-0.360267	0.421088	-1.329615
C	0.872297	0.697140	-1.905127
H	0.110436	3.621611	-3.479817
H	-2.101800	3.106176	-2.523514
C	-2.750113	0.923974	-0.990577
H	1.722582	0.034466	-1.774314
N	-2.766201	-0.246528	-0.303530
C	-3.887241	-0.695524	0.273548
C	-5.076779	0.012130	0.195749
C	-5.089070	1.217950	-0.500775
C	-3.922573	1.673933	-1.097284
H	-5.966915	-0.378681	0.673986
H	-6.001418	1.800183	-0.577945
H	-3.916577	2.611258	-1.641706
Au	-0.818876	-1.136273	-0.095783
S	1.312869	-2.135120	-0.148061
C	2.391891	-0.877332	0.622466
C	3.865500	-1.090105	0.270767
N	4.591969	0.167231	0.641275
C	4.082702	-1.286860	-1.257972
O	4.159646	-0.203678	-1.903845
O	4.130962	-2.463512	-1.663484
H	4.183743	0.721087	1.445888
H	2.096264	0.109975	0.254154
H	2.263823	-0.885780	1.709335
H	4.290450	-1.922288	0.835736
H	5.579711	-0.009402	0.805776

H	4.500762	0.744958	-0.213137
Cl	-1.661914	-2.972452	1.345123
H	-3.794718	-1.635786	0.809313
S	-1.339605	1.297813	2.270335
C	0.408954	1.543862	2.726676
C	1.128883	2.707873	1.986151
H	0.513910	1.732446	3.798622
H	0.964509	0.622326	2.529874
N	0.373809	3.124113	0.769211
H	1.204004	3.576008	2.643584
C	2.532938	2.276392	1.517790
H	0.998036	2.898927	-0.027936
H	-0.524026	2.569333	0.777460
H	0.153712	4.115323	0.746458
O	2.791626	2.416678	0.294676
O	3.262229	1.752451	2.403005

4 TS2

C	1.088993	-2.394062	3.277518
H	1.095898	-3.357710	3.778905
C	1.274079	-1.213889	4.003801
C	1.253272	-0.001989	3.334922
C	1.043200	0.093524	1.945065
C	0.856079	-1.113388	1.227362
C	0.897696	-2.345806	1.907775
H	1.421712	-1.241631	5.078145
H	1.359630	0.920894	3.899483
C	1.006733	1.437932	1.340224
H	0.783258	-3.266604	1.342897
N	0.158025	1.669855	0.316202
C	0.130285	2.894514	-0.224607
C	0.904172	3.960218	0.218322
C	1.778032	3.729533	1.279368
C	1.841313	2.458071	1.834218
H	0.828732	4.931759	-0.256652
H	2.414776	4.523531	1.658229
H	2.544225	2.243614	2.633349
Au	-0.551383	-1.063600	-0.423005
S	1.800576	-1.442959	-0.718275
C	2.436305	0.127126	-1.388417
C	3.676263	0.586514	-0.644114
N	3.985889	2.034706	-0.892482
C	4.983229	-0.175472	-1.035868
O	6.006871	0.563228	-1.076720
O	4.865560	-1.393956	-1.245566
H	3.660648	2.359851	-1.802122
H	2.670461	-0.080290	-2.437846
H	1.634517	0.867417	-1.367921
H	3.531050	0.490430	0.437616
H	3.623709	2.669328	-0.180286
H	5.040885	1.998410	-0.906527
Cl	-1.304658	-0.272047	-2.941343
H	-0.548981	3.028120	-1.065653
S	-2.711901	-1.199834	0.647979

C	-3.931934	-0.773746	-0.648271
C	-4.184926	0.714139	-0.773938
H	-3.606124	-1.200487	-1.599806
H	-4.886832	-1.221063	-0.362202
N	-2.921178	1.499621	-0.872677
H	-4.734592	0.906402	-1.703825
C	-4.997391	1.354416	0.392720
H	-3.201186	2.442646	-0.546908
H	-2.237831	1.124053	-0.197110
H	-2.480891	1.445534	-1.797928
O	-4.637955	2.529737	0.685020
O	-5.920100	0.662818	0.863187

3/ZF R

N	6.695736	-8.241452	-0.732498
C	7.410016	-7.583529	0.370907
C	6.478389	-6.789026	1.260574
O	6.452876	-7.032414	2.455905
C	8.489900	-6.699404	-0.265369
C	8.583088	-7.168862	-1.706779
C	7.216629	-7.757218	-2.005998
H	5.656819	-8.121244	-0.689130
H	6.818831	-9.278764	-0.667986
H	7.921930	-8.358737	0.988970
H	8.207342	-5.624248	-0.250495
H	9.466059	-6.814316	0.254348
H	9.356331	-7.963915	-1.791307
H	8.832139	-6.335201	-2.398043
H	6.553958	-6.958094	-2.406934
H	7.285141	-8.577399	-2.754822
N	5.677704	-5.822024	0.735101
C	4.743578	-5.011622	1.526544
C	3.338739	-5.283006	1.043291
O	2.690184	-4.403066	0.500244
C	5.134512	-3.524722	1.421148
C	6.508705	-3.282382	1.989669
C	6.712607	-3.310772	3.377797
C	7.605513	-3.060194	1.137696
C	7.997513	-3.133051	3.893396
C	8.843481	-2.897611	1.634387
C	9.079418	-2.926932	3.015608
O	10.367200	-2.742457	3.472214
H	5.715120	-5.640754	-0.293793
H	4.774737	-5.294861	2.601831
H	4.401877	-2.906658	1.977431
H	5.098155	-3.205013	0.358935
H	5.882522	-3.475298	4.053568
H	7.462509	-3.029571	0.065543
H	8.148186	-3.155392	4.965219
H	9.671427	-2.736884	0.956894
H	10.361404	-2.778010	4.463371
N	2.805264	-6.522602	1.205810
C	1.471552	-6.885426	0.713549
C	0.414355	-6.356648	1.659132

O	0.400357	-6.742864	2.820330
C	1.376111	-8.412293	0.524982
C	0.054212	-8.834202	-0.133075
C	0.093945	-10.300746	-0.582408
C	-1.159313	-10.703503	-1.376817
N	-2.358886	-10.715083	-0.541579
H	3.354174	-7.236108	1.739014
H	1.326514	-6.427266	-0.287829
H	1.484134	-8.927307	1.505137
H	2.223982	-8.731849	-0.120637
H	-0.153772	-8.185202	-1.012645
H	-0.756365	-8.708508	0.611341
H	0.977624	-10.456191	-1.239338
H	0.207513	-10.963565	0.303144
H	-0.999228	-11.722959	-1.791523
H	-1.306527	-10.010647	-2.234849
H	-3.174649	-11.108619	-1.061507
H	-2.639034	-9.757325	-0.238063
H	-2.237586	-11.296910	0.317578
N	-0.488711	-5.345715	1.212361
C	-1.758935	-5.291544	1.959632
C	-2.650698	-6.519932	1.696433
O	-2.613618	-7.045474	0.600849
C	-2.522201	-4.057597	1.461288
S	-4.268002	-4.042933	1.960693
H	-0.787092	-5.621866	0.279206
H	-1.581309	-5.144652	3.032320
H	-2.013300	-3.161656	1.845201
H	-2.441550	-4.025698	0.364735
N	-3.533772	-7.017389	2.760799
C	-4.409568	-8.122937	2.346276
C	-5.631929	-7.622516	1.615685
O	-5.973704	-8.182823	0.587118
C	-4.787265	-8.860425	3.626236
C	-3.642878	-8.579604	4.585896
C	-2.758300	-7.558765	3.880477
H	-3.854669	-8.847259	1.704114
H	-5.735229	-8.469371	4.059458
H	-4.899502	-9.951199	3.440218
H	-3.071219	-9.508619	4.802719
H	-4.033249	-8.162174	5.540166
H	-2.477664	-6.743375	4.581081
H	-1.834954	-8.070053	3.520998
N	-6.359167	-6.582643	2.109393
C	-7.666976	-6.183076	1.566112
C	-7.551200	-5.763484	0.112130
O	-8.174632	-6.400601	-0.727478
C	-8.287349	-5.061454	2.426981
C	-9.775908	-4.845171	2.112495
C	-10.405737	-3.896120	3.090862
O	-10.919207	-2.863544	2.700443
O	-10.431515	-4.167830	4.278179
H	-6.038240	-6.145834	3.003726
H	-8.338116	-7.069737	1.633906

H	-7.726805	-4.111924	2.281982
H	-8.193088	-5.348774	3.497759
H	-9.883106	-4.450546	1.078331
H	-10.318789	-5.813481	2.168806
N	-6.757045	-4.639839	-0.267368
C	-6.499523	-4.533811	-1.697273
C	-5.683498	-5.633328	-2.387309
O	-5.886261	-5.902983	-3.555852
C	-5.775073	-3.214116	-2.015749
S	-6.810798	-1.754883	-1.732168
H	-5.888528	-4.629578	0.273289
H	-7.445272	-4.489946	-2.253402
H	-4.831431	-3.135447	-1.458465
H	-5.534324	-3.200907	-3.084107
N	-4.656229	-6.328287	-1.606651
C	-3.763508	-7.163188	-2.430904
C	-2.876382	-6.346451	-3.339729
O	-2.937151	-6.529624	-4.544395
H	-4.091284	-5.610909	-1.099812
H	-3.114419	-7.767184	-1.765917
H	-4.363361	-7.892561	-3.019564
N	-2.010099	-5.433087	-2.822312
C	-1.053817	-4.657859	-3.625883
C	0.316558	-4.773657	-3.000477
O	0.491435	-4.350013	-1.868963
C	-1.492257	-3.182103	-3.699679
C	-2.715121	-3.001196	-4.618185
C	-3.337293	-1.598539	-4.519750
C	-2.477675	-0.476113	-5.128251
N	-2.513098	-0.494010	-6.588580
H	-1.994790	-5.286539	-1.788734
H	-1.005003	-5.054021	-4.664407
H	-1.723712	-2.810538	-2.676174
H	-0.650387	-2.580866	-4.104538
H	-3.498584	-3.730454	-4.323533
H	-2.436346	-3.229239	-5.668314
H	-4.329667	-1.608932	-5.020167
H	-3.527404	-1.369946	-3.453449
H	-2.875233	0.498735	-4.772955
H	-1.427788	-0.542424	-4.781452
H	-1.987081	0.316311	-6.985632
H	-3.487565	-0.431555	-6.960009
H	-2.078125	-1.355426	-6.986096
N	1.341123	-5.351122	-3.684985
C	2.667744	-5.596485	-3.103471
C	3.652301	-4.593499	-3.646073
O	3.766941	-4.460561	-4.854115
C	3.124635	-7.026517	-3.410355
O	2.213732	-7.940054	-2.864189
H	1.184081	-5.651403	-4.674404
H	2.628871	-5.504060	-1.998399
H	4.129824	-7.194481	-2.962685
H	3.192769	-7.178807	-4.511229
H	2.574527	-8.842727	-3.063328

N	4.404972	-3.847208	-2.793709
C	5.336918	-2.794770	-3.227087
C	6.760904	-3.225676	-2.965299
O	6.977010	-4.227991	-2.304030
C	5.042367	-1.480055	-2.476108
C	3.590767	-1.087301	-2.573527
C	2.681648	-1.500601	-1.585940
C	3.129506	-0.318592	-3.654865
C	1.329613	-1.168890	-1.689387
C	1.777923	0.031146	-3.742571
C	0.879844	-0.396486	-2.761252
H	4.324099	-4.022118	-1.766992
H	5.230739	-2.602737	-4.317709
H	5.331883	-1.577415	-1.406703
H	5.667297	-0.666915	-2.900601
H	3.018929	-2.083707	-0.740161
H	3.815185	0.008929	-4.426340
H	0.631989	-1.512591	-0.937991
H	1.425286	0.627545	-4.574048
H	-0.165014	-0.134444	-2.832089
N	7.795710	-2.487630	-3.448081
C	9.200854	-2.739696	-3.093505
C	9.673360	-1.831967	-1.972906
O	10.762128	-2.059819	-1.470699
C	10.090414	-2.584051	-4.334545
O	10.057987	-1.261998	-4.799804
H	7.578916	-1.647752	-4.032722
H	9.319120	-3.788546	-2.741670
H	11.136961	-2.868232	-4.081734
H	9.727092	-3.270937	-5.131594
H	10.656513	-1.233642	-5.590741
N	8.925602	-0.778144	-1.533931
C	9.373084	0.169553	-0.501728
C	8.331262	0.284755	0.589453
O	7.146229	0.230435	0.300256
C	9.614096	1.551349	-1.134625
C	10.779556	1.532004	-2.137082
C	11.037250	2.876629	-2.768351
O	10.352775	3.844770	-2.479806
N	12.035855	3.012852	-3.668263
H	8.016260	-0.558721	-1.995949
H	10.325601	-0.171122	-0.039038
H	9.848233	2.285243	-0.332646
H	8.692281	1.882485	-1.658128
H	10.558504	0.807622	-2.949207
H	11.704555	1.201695	-1.616769
H	12.226860	3.937045	-4.116626
H	12.632461	2.196887	-3.931865
N	8.711784	0.487989	1.881044
C	7.749905	0.782058	2.957940
C	7.149110	2.167601	2.818276
O	6.079334	2.398141	3.358081
C	8.385331	0.583745	4.351664
C	9.412721	1.665340	4.734414

C	10.073009	1.338676	6.078761
C	11.086651	2.419930	6.472920
N	11.712790	2.101924	7.753077
H	9.733835	0.546143	2.088477
H	6.919313	0.043304	2.881161
H	7.574980	0.574091	5.113897
H	8.874981	-0.411943	4.380716
H	10.199651	1.742116	3.955198
H	8.903236	2.650164	4.814510
H	9.291014	1.265295	6.866272
H	10.593919	0.358581	6.005708
H	11.873402	2.497857	5.690104
H	10.572550	3.403498	6.551273
H	12.404532	2.834646	8.027722
H	11.013200	2.039498	8.526634
H	12.228509	1.193739	7.722111
N	7.786231	3.149482	2.121753
C	7.157690	4.441780	1.811675
C	6.024720	4.265324	0.825975
O	4.988540	4.879898	1.004758
C	8.194282	5.438721	1.270918
O	8.733699	4.988482	0.057824
H	8.731906	2.956501	1.723550
H	6.753214	4.876204	2.754101
H	7.710388	6.429003	1.122052
H	9.008393	5.559906	2.020202
H	9.377861	5.685010	-0.232723
N	6.150084	3.426363	-0.238526
C	5.078940	3.197817	-1.223549
C	3.930232	2.390089	-0.649046
O	2.874620	2.371371	-1.260641
C	5.638629	2.492309	-2.476362
C	6.660391	3.306638	-3.226454
O	6.946634	4.439556	-2.879206
O	7.209376	2.824211	-4.200346
H	7.034223	2.881176	-0.334988
H	4.669695	4.181407	-1.538072
H	4.806238	2.260586	-3.176394
H	6.105574	1.532037	-2.170326
N	4.043652	1.704489	0.521879
C	2.888082	1.091424	1.192191
C	1.947504	2.166542	1.682279
O	0.770885	2.108745	1.368794
C	3.360797	0.193797	2.358523
C	2.215975	-0.479767	3.155937
C	1.389843	-1.441757	2.290097
C	2.788217	-1.224872	4.368181
H	4.962656	1.680269	1.013536
H	2.361649	0.449718	0.455645
H	3.961639	0.811509	3.060499
H	4.039518	-0.585225	1.950616
H	1.536721	0.306588	3.548816
H	0.828048	-0.882452	1.516594
H	2.045829	-2.185043	1.794284

H	0.643820	-1.981151	2.912194
H	3.351037	-0.522692	5.019750
H	1.965749	-1.665148	4.971594
H	3.471073	-2.038901	4.045586
N	2.403164	3.181374	2.465353
C	1.515979	4.198029	3.057213
C	1.125915	5.284359	2.069317
O	0.225135	6.046597	2.380073
C	2.121893	4.805376	4.353467
C	2.376541	3.718851	5.408802
C	3.403063	5.622799	4.105400
H	3.429980	3.238693	2.640400
H	0.570474	3.693898	3.357299
H	1.366621	5.501350	4.783561
H	2.705079	4.183860	6.362625
H	1.444593	3.148313	5.606860
H	3.163057	3.010308	5.072067
H	3.695180	6.151019	5.037811
H	4.250526	4.975236	3.801377
H	3.238689	6.390798	3.321512
N	1.738124	5.419152	0.859835
C	1.289560	6.388809	-0.153194
C	0.067693	5.886578	-0.889051
O	-0.649682	6.704317	-1.439107
C	2.422083	6.695218	-1.150991
C	3.536379	7.526094	-0.497091
C	4.797371	7.550282	-1.367471
C	5.894666	8.403973	-0.721849
N	7.134823	8.319896	-1.487931
H	2.472082	4.734528	0.577189
H	1.011018	7.341277	0.351990
H	2.829652	5.740553	-1.547686
H	2.020623	7.271116	-2.014407
H	3.170234	8.565024	-0.348396
H	3.790487	7.108364	0.500109
H	4.551452	7.963696	-2.370079
H	5.174504	6.513615	-1.496398
H	5.561323	9.464222	-0.674722
H	6.082210	8.052757	0.316347
H	7.885973	8.903072	-1.056220
H	7.500350	7.342089	-1.535664
H	7.012553	8.657485	-2.469090
N	-0.241353	4.560637	-0.939052
C	-1.488665	4.037935	-1.514563
C	-2.490674	3.666240	-0.428142
O	-3.583857	3.246330	-0.767814
C	-1.184079	2.833868	-2.421432
C	-0.400299	3.226394	-3.645698
N	0.933103	3.733756	-3.641757
C	-0.810154	3.102258	-4.904827
C	1.247574	3.892067	-4.941101
N	0.242996	3.531621	-5.762001
H	0.408794	3.864516	-0.512764
H	-1.973737	4.811049	-2.147515

H	-0.613460	2.069044	-1.854587
H	-2.141518	2.382389	-2.756280
H	1.547842	3.938743	-2.825738
H	-1.770807	2.725474	-5.239355
H	2.202302	4.264156	-5.287817
N	-2.226243	3.846249	0.897873
C	-3.286892	3.856742	1.919687
C	-4.036305	5.179141	1.930200
O	-5.074189	5.244685	2.568621
C	-2.692013	3.607182	3.321345
C	-2.131307	2.184306	3.472798
C	-1.441807	1.962048	4.796035
O	-1.418803	2.840434	5.642758
N	-0.839745	0.779739	5.050647
H	-1.290090	4.193574	1.193930
H	-4.015891	3.043490	1.703518
H	-3.483285	3.748225	4.091153
H	-1.891210	4.354486	3.517758
H	-1.403858	1.984861	2.661750
H	-2.962745	1.453302	3.378801
H	-0.839793	0.014683	4.341115
H	-0.351121	0.615129	5.959266
N	-3.561833	6.284531	1.285309
C	-4.143043	7.627693	1.436933
C	-4.674216	8.125574	0.112685
O	-5.839645	8.477226	0.039270
C	-3.083066	8.600292	1.987969
C	-2.696528	8.256981	3.434657
C	-1.671952	9.243195	4.008817
N	-0.332549	8.980061	3.472076
C	0.306257	9.696184	2.500032
N	-0.317573	10.686938	1.810747
N	1.593761	9.397402	2.199722
H	-2.671179	6.223876	0.745164
H	-4.991497	7.612437	2.158235
H	-3.493303	9.634317	1.964039
H	-2.177506	8.570341	1.343794
H	-3.609368	8.292321	4.068677
H	-2.285068	7.225180	3.488443
H	-1.634480	9.099748	5.110509
H	-1.993941	10.293292	3.861987
H	0.188483	8.197435	3.929586
H	0.194040	11.214937	1.068761
H	-1.320819	10.932295	1.939349
H	2.110498	9.925749	1.461900
H	2.101659	8.632021	2.696827
N	-3.867261	8.214726	-0.978021
C	-4.331703	8.742669	-2.271001
C	-5.146276	7.704669	-3.009617
O	-6.198065	8.038353	-3.531139
C	-3.149006	9.237328	-3.140750
O	-2.241659	8.195753	-3.400013
C	-2.401820	10.387841	-2.460871
H	-2.888413	7.861778	-0.905273

H	-4.984560	9.626816	-2.085249
H	-3.557236	9.637974	-4.099961
H	-2.537199	7.749778	-4.236245
H	-1.599292	10.760194	-3.132317
H	-3.096774	11.227660	-2.247153
H	-1.935335	10.052153	-1.510215
N	-4.712211	6.419221	-3.104674
C	-5.417115	5.388514	-3.879286
C	-6.697084	4.985638	-3.182588
O	-7.737695	4.986145	-3.820215
C	-4.494951	4.172688	-4.099145
C	-5.121750	3.110269	-4.959000
N	-5.373026	1.772987	-4.539168
C	-5.486028	3.232853	-6.229869
C	-5.889726	1.195489	-5.637981
N	-5.968866	2.055789	-6.671827
H	-3.847419	6.147445	-2.585928
H	-5.660874	5.808088	-4.882103
H	-4.230229	3.725789	-3.122716
H	-3.552690	4.509352	-4.582596
H	-5.166326	1.322779	-3.620960
H	-5.405319	4.126024	-6.835162
H	-6.192576	0.158340	-5.690693
N	-6.695249	4.628756	-1.868908
C	-7.887812	4.113934	-1.176119
C	-8.591465	5.223142	-0.429547
O	-9.789578	5.377926	-0.603289
C	-7.531612	2.943388	-0.229689
O	-6.611449	3.347505	0.751805
C	-6.936215	1.770156	-1.003172
H	-5.804880	4.714264	-1.328225
H	-8.605327	3.702356	-1.923223
H	-8.472569	2.574375	0.246725
H	-7.130815	3.573169	1.567196
H	-6.754255	0.930795	-0.303239
H	-7.635568	1.426829	-1.794863
H	-5.970005	2.057093	-1.466328
N	-7.911619	6.033010	0.427347
C	-8.556719	7.042341	1.270570
C	-8.157276	6.845989	2.666692
O	-7.829041	6.685242	3.805695
H	-8.241387	8.053770	0.944709
H	-9.664026	6.988528	1.211291
H	-6.874113	5.935429	0.504007
Au	-6.517349	-1.443912	0.577970
Cl	-9.000428	-1.443573	0.624073
N	-6.372367	-1.387140	2.772233
C	-5.234456	-1.660222	3.446813
C	-5.282202	-1.949033	4.819573
C	-6.485430	-1.956856	5.491400
C	-7.646463	-1.639675	4.787231
C	-7.546853	-1.366678	3.436935
C	-3.862496	-1.538222	2.854842
H	-4.332373	-2.156311	5.297496

H	-6.522942	-2.195847	6.551456
H	-8.621885	-1.613749	5.261683
H	-8.419754	-1.153267	2.827250
O	-2.908961	-1.630853	3.639089
C	-3.621948	-1.014019	1.478762
C	-2.301889	-0.571169	1.281542
C	-1.859576	-0.090153	0.061727
C	-2.754161	-0.038948	-0.995501
C	-4.075988	-0.433440	-0.813186
C	-4.526243	-0.938126	0.408106
H	-1.638452	-0.647825	2.135625
H	-0.831416	0.239913	-0.056634
H	-2.441481	0.288581	-1.980755
H	-4.765801	-0.368519	-1.645755

3/ZF I

N	5.357533	-8.858025	-1.418876
C	6.147057	-8.453938	-0.247008
C	5.331022	-7.632994	0.728715
O	5.237353	-8.008976	1.885647
C	7.360039	-7.676279	-0.777036
C	7.359616	-7.908393	-2.279197
C	5.919721	-8.247014	-2.617239
H	4.337948	-8.636899	-1.333042
H	5.369769	-9.900317	-1.513221
H	6.520765	-9.365596	0.276443
H	7.275826	-6.586155	-0.576561
H	8.305144	-8.043597	-0.320821
H	8.011673	-8.775782	-2.523249
H	7.711557	-7.013136	-2.835441
H	5.368453	-7.310783	-2.859909
H	5.860251	-8.935388	-3.489441
N	4.704524	-6.497149	0.318352
C	3.862852	-5.663008	1.183820
C	2.464707	-5.634882	0.614042
O	1.995570	-4.595129	0.179683
C	4.488352	-4.259150	1.309551
C	5.768031	-4.311446	2.101451
C	5.724346	-4.392631	3.502012
C	7.013929	-4.314980	1.450079
C	6.912659	-4.477803	4.229052
C	8.157832	-4.406102	2.150492
C	8.145075	-4.482028	3.550515
O	9.294111	-4.550637	4.199949
H	4.801669	-6.201626	-0.679146
H	3.782086	-6.094775	2.206189
H	3.784718	-3.571937	1.822482
H	4.676979	-3.840987	0.297670
H	4.775724	-4.393416	4.024614
H	7.059775	-4.254226	0.370302
H	6.873109	-4.535547	5.309542
H	9.103679	-4.421095	1.625342
H	9.193917	-4.596726	5.208340
N	1.732447	-6.780505	0.583643

C	0.351476	-6.827170	0.091971
C	-0.585935	-6.376823	1.187658
O	-0.710618	-7.065795	2.192227
C	0.013315	-8.242440	-0.415150
C	-1.368459	-8.298516	-1.085150
C	-1.604185	-9.656238	-1.759899
C	-2.943450	-9.710431	-2.512952
N	-4.083162	-9.759001	-1.598800
H	2.135432	-7.642981	1.017637
H	0.251930	-6.140727	-0.774834
H	0.049159	-8.969476	0.426093
H	0.789764	-8.542392	-1.152600
H	-1.449321	-7.490615	-1.845971
H	-2.142637	-8.136774	-0.308581
H	-0.789713	-9.839313	-2.494479
H	-1.565643	-10.468385	-1.001345
H	-2.953604	-10.623860	-3.147335
H	-3.039627	-8.832037	-3.188866
H	-4.979317	-9.876153	-2.122119
H	-4.181899	-8.885428	-1.037998
H	-4.016952	-10.557220	-0.928147
N	-1.238691	-5.113155	1.076537
C	-2.420496	-4.970467	1.937535
C	-3.599759	-5.858027	1.448315
O	-3.642168	-6.114372	0.260983
C	-2.850518	-3.501966	1.844121
S	-4.290598	-3.177004	2.922331
H	-1.620497	-5.069254	0.133998
H	-2.148404	-5.151116	2.984491
H	-1.996795	-2.873581	2.121031
H	-3.103352	-3.279022	0.800573
N	-4.679001	-6.297876	2.351431
C	-5.403484	-7.475312	1.836554
C	-6.533445	-7.079005	0.914341
O	-6.586280	-7.575220	-0.199355
C	-5.923386	-8.254567	3.050235
C	-5.197684	-7.666153	4.244261
C	-4.158434	-6.737074	3.649841
H	-4.701962	-8.153798	1.293180
H	-7.022715	-8.151792	3.190622
H	-5.684902	-9.336272	2.946009
H	-4.723222	-8.456541	4.865902
H	-5.908946	-7.082133	4.869526
H	-3.998714	-5.865983	4.320631
H	-3.202535	-7.299182	3.530923
N	-7.508090	-6.224646	1.330422
C	-8.752287	-6.017176	0.572635
C	-8.497411	-5.161867	-0.649916
O	-8.837752	-5.592501	-1.744185
C	-9.839408	-5.394842	1.471809
C	-11.222411	-5.403373	0.803081
C	-12.258855	-4.809867	1.710374
O	-12.834632	-3.784269	1.393887
O	-12.537385	-5.356768	2.762690

H	-7.402295	-5.765098	2.263979
H	-9.128592	-7.014547	0.246841
H	-9.566305	-4.351260	1.731190
H	-9.895506	-5.979469	2.416902
H	-11.183195	-4.820731	-0.143380
H	-11.517122	-6.445867	0.554060
N	-7.948870	-3.852285	-0.524576
C	-7.410057	-3.270600	-1.739080
C	-6.500127	-4.131441	-2.617018
O	-6.528048	-4.045929	-3.829979
C	-6.558624	-2.008630	-1.448079
S	-7.145075	-0.875717	-0.139208
H	-7.343287	-3.779201	0.285849
H	-8.237537	-2.963596	-2.396234
H	-5.533870	-2.304576	-1.187911
H	-6.473943	-1.454167	-2.388877
N	-5.609801	-5.072250	-1.928592
C	-4.871213	-5.973574	-2.828284
C	-3.851900	-5.249751	-3.671233
O	-3.929926	-5.329062	-4.886305
H	-4.944479	-4.530025	-1.334878
H	-4.339287	-6.730295	-2.217105
H	-5.588291	-6.530470	-3.471594
N	-2.860131	-4.522791	-3.087979
C	-1.844451	-3.774797	-3.842127
C	-0.479013	-4.097654	-3.279839
O	-0.226463	-3.806678	-2.121296
C	-2.140716	-2.262991	-3.770219
C	-3.370622	-1.897299	-4.624476
C	-3.841532	-0.444349	-4.451506
C	-2.785509	0.623583	-4.776956
N	-2.315698	0.536892	-6.157517
H	-2.817883	-4.472793	-2.044998
H	-1.859377	-4.072155	-4.914078
H	-2.309567	-1.961774	-2.712780
H	-1.258522	-1.708843	-4.156724
H	-4.222486	-2.546979	-4.334587
H	-3.157599	-2.098836	-5.696030
H	-4.725964	-0.281430	-5.104617
H	-4.184217	-0.300256	-3.406946
H	-3.240458	1.623153	-4.613262
H	-1.929679	0.538247	-4.080293
H	-1.695864	1.343518	-6.393841
H	-3.104007	0.548551	-6.842917
H	-1.754470	-0.325816	-6.332192
N	0.449230	-4.730602	-4.049320
C	1.709669	-5.273303	-3.522579
C	2.856642	-4.379261	-3.912886
O	2.988283	-4.052081	-5.081611
C	1.936974	-6.694361	-4.050459
O	0.862988	-7.513791	-3.680809
H	0.240770	-4.894124	-5.061058
H	1.665123	-5.344538	-2.416766
H	2.879484	-7.100490	-3.617840

H	2.029762	-6.679651	-5.160048
H	1.077128	-8.420518	-4.022331
N	3.736791	-3.947382	-2.969492
C	4.820804	-2.991822	-3.237838
C	6.166393	-3.620361	-2.978965
O	6.246401	-4.652709	-2.333604
C	4.636669	-1.746425	-2.355254
C	3.434016	-0.962260	-2.801592
C	2.192574	-1.136657	-2.168546
C	3.533089	-0.094911	-3.901193
C	1.067810	-0.445337	-2.626970
C	2.407157	0.592836	-4.357015
C	1.176263	0.413693	-3.723719
H	3.617378	-4.270714	-1.982974
H	4.809171	-2.676952	-4.305299
H	4.530849	-2.043462	-1.289283
H	5.534319	-1.098028	-2.414075
H	2.093682	-1.821024	-1.336185
H	4.479034	0.036667	-4.411666
H	0.110980	-0.585925	-2.141590
H	2.486470	1.258018	-5.207030
H	0.308978	0.938643	-4.090732
N	7.287787	-3.029800	-3.470558
C	8.639352	-3.531237	-3.193192
C	9.044764	-3.203138	-1.777180
O	9.538438	-4.081613	-1.089355
C	9.653507	-2.968131	-4.202142
O	9.697666	-1.568128	-4.144055
H	7.184628	-2.128709	-3.991804
H	8.646308	-4.637479	-3.323290
H	10.662277	-3.385593	-3.982210
H	9.360051	-3.288250	-5.226968
H	10.384183	-1.286239	-4.802691
N	8.865781	-1.949570	-1.275733
C	9.331366	-1.527788	0.054075
C	8.163641	-1.103036	0.916994
O	7.066288	-0.921074	0.412720
C	10.334996	-0.368047	-0.089972
C	11.584068	-0.785608	-0.885115
C	12.604433	0.317193	-1.007226
O	12.406824	1.410790	-0.503423
N	13.752564	0.094447	-1.683934
H	8.415603	-1.222815	-1.878234
H	9.845175	-2.364437	0.574413
H	10.659861	-0.036228	0.920131
H	9.840435	0.489421	-0.598571
H	11.287142	-1.095117	-1.909349
H	12.062573	-1.655082	-0.384450
H	14.470081	0.847967	-1.778918
H	13.941726	-0.833774	-2.124232
N	8.343164	-0.917382	2.253787
C	7.315126	-0.322553	3.123784
C	7.218325	1.181942	2.947820
O	6.246513	1.758069	3.408446

C	7.543122	-0.707181	4.601774
C	8.746126	-0.004105	5.257470
C	8.959128	-0.511478	6.688281
C	10.149259	0.192076	7.351958
N	10.347630	-0.297156	8.713300
H	9.293375	-1.097049	2.648117
H	6.330580	-0.756828	2.837435
H	6.625165	-0.459667	5.179756
H	7.682989	-1.806434	4.663535
H	9.669083	-0.189229	4.669570
H	8.565294	1.092380	5.287328
H	8.041423	-0.322803	7.287688
H	9.148049	-1.607520	6.666887
H	11.071742	0.006278	6.758356
H	9.966540	1.289185	7.380271
H	11.157033	0.178068	9.171394
H	9.512617	-0.124641	9.317204
H	10.545082	-1.322935	8.736101
N	8.183736	1.891432	2.298046
C	8.021486	3.305857	1.932748
C	6.974622	3.462893	0.854256
O	6.158883	4.362954	0.952433
C	9.357550	3.915091	1.479164
O	9.834886	3.271442	0.328821
H	9.041944	1.401877	1.964796
H	7.701339	3.872658	2.836061
H	9.214943	4.999707	1.270446
H	10.101775	3.812786	2.300493
H	10.682341	3.728386	0.089140
N	6.932749	2.613606	-0.208735
C	5.912688	2.707690	-1.265386
C	4.575336	2.142543	-0.818759
O	3.590467	2.379078	-1.499937
C	6.395906	1.995185	-2.544800
C	7.645234	2.590704	-3.139321
O	8.182940	3.563829	-2.640154
O	8.127653	2.089373	-4.138564
H	7.606208	1.814653	-0.241232
H	5.753486	3.778855	-1.513865
H	5.596113	2.040793	-3.315215
H	6.594882	0.928419	-2.313384
N	4.448454	1.407241	0.321560
C	3.144588	1.034185	0.890349
C	2.443955	2.259209	1.437452
O	1.270838	2.438710	1.157877
C	3.333260	-0.029048	1.996524
C	2.007188	-0.577554	2.579673
C	1.254891	-1.452232	1.567695
C	2.284878	-1.375598	3.859762
H	5.299616	1.174096	0.876573
H	2.528439	0.589723	0.080804
H	3.931271	0.423457	2.817868
H	3.931009	-0.874568	1.588260
H	1.347465	0.268223	2.860907

H	0.886030	-0.842314	0.719516
H	1.916762	-2.246644	1.174206
H	0.371206	-1.926760	2.044387
H	2.769798	-0.724922	4.618645
H	1.333624	-1.752521	4.292901
H	2.951786	-2.238207	3.650715
N	3.104831	3.148528	2.230504
C	2.463176	4.352440	2.791886
C	2.208144	5.422612	1.742445
O	1.472213	6.351600	2.035757
C	3.255272	4.934186	3.998964
C	3.335777	3.929365	5.156230
C	4.661911	5.432999	3.628481
H	4.120795	2.989825	2.401570
H	1.465388	4.051724	3.182164
H	2.688462	5.813511	4.380119
H	3.886839	4.374124	6.012226
H	2.316200	3.673692	5.508334
H	3.856069	2.998770	4.845199
H	5.080578	6.026557	4.468888
H	5.351106	4.586456	3.439076
H	4.631361	6.089611	2.734164
N	2.762200	5.365967	0.498175
C	2.389229	6.303046	-0.574373
C	0.966837	6.066802	-1.023537
O	0.280030	7.032710	-1.310179
C	3.337980	6.183522	-1.782653
C	4.744966	6.700710	-1.457926
C	5.660984	6.613402	-2.684581
C	7.105277	6.975713	-2.319272
N	7.962230	6.945338	-3.501090
H	3.408709	4.583200	0.258771
H	2.461557	7.341311	-0.183158
H	3.396149	5.123362	-2.110740
H	2.930382	6.781486	-2.628493
H	4.684607	7.760210	-1.125802
H	5.174810	6.102595	-0.629735
H	5.290751	7.311971	-3.466669
H	5.639799	5.582643	-3.100657
H	7.134697	7.994001	-1.872113
H	7.491501	6.251786	-1.567308
H	8.952388	7.168995	-3.255516
H	7.967429	6.006554	-3.959280
H	7.662161	7.642001	-4.219578
N	0.457559	4.806430	-1.112995
C	-0.920631	4.513486	-1.536778
C	-1.838810	4.245658	-0.353449
O	-2.995990	3.937522	-0.582676
C	-0.928453	3.338652	-2.533160
C	-0.266805	3.716199	-3.830259
N	-0.958848	3.933542	-5.057316
C	1.038971	3.854460	-4.039787
C	0.006935	4.203450	-5.955884
N	1.238993	4.172976	-5.413218

H	1.068636	3.996655	-0.865827
H	-1.349451	5.390834	-2.068000
H	-1.976015	3.046266	-2.758459
H	-0.417255	2.454417	-2.099009
H	-1.985811	3.892395	-5.235476
H	1.829350	3.713088	-3.312444
H	-0.184389	4.417442	-6.998766
N	-1.442777	4.440592	0.937861
C	-2.419076	4.689657	2.013889
C	-2.954145	6.112251	1.964139
O	-3.878887	6.407469	2.703088
C	-1.801010	4.408499	3.398904
C	-1.508296	2.916389	3.587214
C	-1.081692	2.607055	4.994645
O	-1.855532	2.806738	5.915660
N	0.137714	2.082742	5.248578
H	-0.451521	4.691417	1.138685
H	-3.284033	4.002321	1.885808
H	-2.517636	4.729176	4.187523
H	-0.866745	4.998243	3.527807
H	-0.718657	2.613172	2.870708
H	-2.424192	2.328176	3.367556
H	0.818984	1.902313	4.479197
H	0.425087	1.855702	6.226950
N	-2.435097	7.046265	1.117754
C	-2.924819	8.427823	1.013972
C	-3.664060	8.619144	-0.290889
O	-4.776754	9.118601	-0.273055
C	-1.744924	9.413901	1.096624
C	-1.035809	9.343735	2.457085
C	0.119633	10.346297	2.554649
N	1.261403	9.913904	1.740648
C	1.665867	10.470971	0.560880
N	0.893777	11.363321	-0.112159
N	2.860341	10.106487	0.033932
H	-1.608214	6.806510	0.531498
H	-3.622303	8.663851	1.849503
H	-2.129031	10.446983	0.944973
H	-1.016117	9.192553	0.286530
H	-1.770612	9.573704	3.259302
H	-0.645430	8.318032	2.635812
H	0.450079	10.392087	3.614919
H	-0.218759	11.372249	2.307215
H	1.857540	9.164830	2.161427
H	1.225633	11.772280	-1.014202
H	-0.066269	11.630734	0.188463
H	3.191690	10.499904	-0.875180
H	3.488837	9.437922	0.532886
N	-3.091716	8.270154	-1.474887
C	-3.714380	8.543153	-2.778814
C	-4.825301	7.557381	-3.061116
O	-5.909766	7.980926	-3.427822
C	-2.664644	8.538640	-3.919842
O	-1.982066	7.310525	-3.975699

C	-1.641993	9.663295	-3.736046
H	-2.170481	7.782835	-1.460402
H	-4.158531	9.565299	-2.754805
H	-3.189318	8.732837	-4.886516
H	-2.472503	6.729416	-4.613896
H	-0.933968	9.667666	-4.591747
H	-2.152944	10.648966	-3.700077
H	-1.060097	9.522437	-2.800322
N	-4.624944	6.218676	-2.924470
C	-5.643900	5.221946	-3.281809
C	-6.738286	5.187893	-2.241578
O	-7.900316	5.270114	-2.605444
C	-4.997660	3.831976	-3.442824
C	-5.969721	2.806088	-3.956223
N	-6.400738	1.661343	-3.227981
C	-6.547392	2.792918	-5.151545
C	-7.236761	1.047152	-4.083858
N	-7.341108	1.709717	-5.252282
H	-3.717907	5.884846	-2.527359
H	-6.081227	5.503108	-4.267353
H	-4.595848	3.484368	-2.470328
H	-4.149071	3.902547	-4.154517
H	-6.125223	1.350862	-2.271069
H	-6.410977	3.525689	-5.935842
H	-7.758624	0.125489	-3.864620
N	-6.438565	5.054060	-0.921407
C	-7.459745	4.823487	0.111724
C	-7.878831	6.129579	0.745171
O	-9.069824	6.367530	0.864949
C	-6.968098	3.809638	1.173127
O	-5.872539	4.317191	1.889308
C	-6.560466	2.486630	0.524507
H	-5.435597	5.081763	-0.628837
H	-8.363226	4.367654	-0.355836
H	-7.814354	3.587325	1.867543
H	-6.231556	4.755698	2.704427
H	-6.320223	1.742054	1.310040
H	-7.393107	2.092457	-0.095177
H	-5.660640	2.617590	-0.113104
N	-6.951210	7.028688	1.174411
C	-7.287841	8.279844	1.857106
C	-6.597159	8.337820	3.147889
O	-6.031700	8.384637	4.200973
H	-6.961335	9.137938	1.235431
H	-8.379059	8.377304	2.036271
H	-5.938656	6.833421	1.006995
Au	-5.682809	-1.721309	1.601527
Cl	-7.566805	-2.723406	2.807857
N	-5.225566	0.557544	3.570295
C	-4.291054	-0.196597	4.163577
C	-4.399646	-0.651830	5.480000
C	-5.547965	-0.348910	6.192615
C	-6.538241	0.413349	5.576604
C	-6.324275	0.845581	4.272639

C	-3.043847	-0.517149	3.391720
H	-3.592560	-1.249626	5.890549
H	-5.681036	-0.711833	7.208977
H	-7.463452	0.657703	6.090484
H	-7.076344	1.440695	3.756116
O	-2.012226	-0.771625	4.009944
C	-3.017010	-0.324929	1.908733
C	-1.820488	0.238851	1.444811
C	-1.648876	0.599024	0.119964
C	-2.690533	0.375823	-0.771077
C	-3.856275	-0.258078	-0.344448
C	-4.037307	-0.641272	0.985676
H	-1.033413	0.384339	2.177480
H	-0.716905	1.051236	-0.206030
H	-2.599599	0.689771	-1.806131
H	-4.653181	-0.429394	-1.056940

3/ZF P

N	4.218207	-9.119782	-0.251258
C	4.794285	-8.636211	1.011526
C	3.948808	-7.552214	1.644352
O	3.550752	-7.703552	2.787718
C	6.211271	-8.141524	0.690030
C	6.511072	-8.677962	-0.698827
C	5.147811	-8.850268	-1.341948
H	3.274375	-8.718965	-0.461902
H	4.032955	-10.147857	-0.187358
H	4.879829	-9.488702	1.726214
H	6.268741	-7.031094	0.668029
H	6.950113	-8.518845	1.430257
H	7.011098	-9.668148	-0.617246
H	7.156046	-7.983523	-1.279512
H	4.864339	-7.904064	-1.854090
H	5.152088	-9.677345	-2.086236
N	3.636170	-6.426694	0.946820
C	2.848733	-5.314179	1.492176
C	1.584750	-5.166425	0.675443
O	1.387844	-4.151855	0.025211
C	3.706062	-4.033031	1.488930
C	4.935815	-4.182213	2.347363
C	4.822397	-4.209080	3.745977
C	6.208064	-4.300932	1.758109
C	5.966999	-4.351412	4.532338
C	7.307537	-4.452284	2.516040
C	7.226163	-4.477779	3.914991
O	8.387595	-4.620815	4.644155
H	3.986482	-6.330238	-0.033175
H	2.537437	-5.515311	2.540811
H	3.108792	-3.177778	1.866348
H	3.999801	-3.799400	0.444967
H	3.854356	-4.121094	4.222650
H	6.312269	-4.283189	0.679790
H	5.872933	-4.366034	5.610746
H	8.272767	-4.552956	2.038780

H	8.161175	-4.604043	5.609630
N	0.667458	-6.171724	0.671476
C	-0.549926	-6.153130	-0.148752
C	-1.701511	-5.653765	0.690763
O	-2.642797	-6.386289	0.967335
C	-0.791330	-7.552489	-0.754859
C	-1.831844	-7.517887	-1.885894
C	-2.111186	-8.925957	-2.426864
C	-3.040811	-8.906380	-3.650839
N	-4.415303	-8.565552	-3.287536
H	0.823799	-6.995222	1.297393
H	-0.423956	-5.444569	-0.994913
H	-1.111031	-8.261656	0.040401
H	0.168585	-7.930213	-1.169075
H	-1.454092	-6.873429	-2.708727
H	-2.777082	-7.081344	-1.506208
H	-1.151311	-9.396520	-2.729782
H	-2.556777	-9.556547	-1.626726
H	-3.032362	-9.917025	-4.115028
H	-2.661947	-8.185529	-4.408935
H	-5.051262	-8.643347	-4.112254
H	-4.505074	-7.586846	-2.937452
H	-4.796853	-9.199752	-2.550239
N	-1.710649	-4.288799	1.076934
C	-2.304407	-4.040865	2.394292
C	-3.828975	-3.757599	2.246656
O	-4.220779	-3.282406	1.204056
C	-1.565047	-2.833887	3.040944
S	-2.456210	-1.269853	3.251432
H	-2.270196	-3.782319	0.395687
H	-2.126723	-4.889954	3.065246
H	-1.267085	-3.068083	4.066765
H	-0.641821	-2.689424	2.473273
N	-4.816850	-4.126210	3.260497
C	-5.040442	-5.572096	3.347458
C	-6.035267	-5.998396	2.297339
O	-5.724022	-6.867295	1.498951
C	-5.540069	-5.850561	4.770949
C	-5.336137	-4.548466	5.534614
C	-4.501787	-3.678724	4.613753
H	-4.087044	-6.130442	3.206285
H	-6.612726	-6.145160	4.801100
H	-4.940641	-6.664860	5.234577
H	-4.822678	-4.718564	6.505941
H	-6.319377	-4.060589	5.716789
H	-4.764056	-2.606525	4.738855
H	-3.429965	-3.835852	4.858783
N	-7.250752	-5.393237	2.217169
C	-8.173574	-5.606016	1.095178
C	-7.664331	-4.870380	-0.120788
O	-7.560863	-5.467911	-1.183133
C	-9.581064	-5.113541	1.476134
C	-10.635323	-5.470696	0.418239
C	-11.991252	-4.980842	0.831456

O	-12.560344	-4.127523	0.174547
O	-12.533303	-5.440280	1.820967
H	-7.466301	-4.626361	2.895416
H	-8.237407	-6.696840	0.878101
H	-9.557859	-4.012471	1.616937
H	-9.869610	-5.578078	2.445146
H	-10.359244	-5.010511	-0.555832
H	-10.675592	-6.572917	0.280382
N	-7.286910	-3.505372	-0.007758
C	-7.363704	-2.722926	-1.241329
C	-6.222330	-2.937672	-2.225226
O	-6.378099	-2.930576	-3.430835
C	-7.391296	-1.218460	-0.877340
S	-9.049685	-0.530662	-0.547971
H	-6.369555	-3.443657	0.424486
H	-8.293816	-2.940389	-1.777346
H	-6.697467	-1.044079	-0.042547
H	-7.020777	-0.633177	-1.726371
N	-4.887506	-3.118047	-1.664485
C	-4.363605	-4.462140	-1.960264
C	-3.469799	-4.478634	-3.170516
O	-3.810663	-5.117749	-4.152135
H	-4.263200	-2.363932	-2.030838
H	-3.777744	-4.811250	-1.089874
H	-5.184286	-5.201931	-2.086073
N	-2.281392	-3.817490	-3.150552
C	-1.286162	-3.888988	-4.229702
C	0.035766	-4.338962	-3.650690
O	0.555491	-3.674143	-2.769180
C	-1.131852	-2.514837	-4.906980
C	-2.419710	-2.042044	-5.620146
C	-3.139366	-0.907177	-4.867428
C	-2.460775	0.465545	-5.037678
N	-2.745018	1.057853	-6.343162
H	-2.034108	-3.265021	-2.297510
H	-1.601869	-4.616878	-5.009312
H	-0.812673	-1.774834	-4.148573
H	-0.315778	-2.581736	-5.660453
H	-3.129365	-2.888769	-5.741122
H	-2.169920	-1.711741	-6.649933
H	-4.191018	-0.843466	-5.221548
H	-3.182550	-1.154199	-3.785876
H	-2.844956	1.144842	-4.248714
H	-1.362440	0.385817	-4.899523
H	-2.366967	2.029555	-6.403521
H	-3.769666	1.121354	-6.534257
H	-2.312567	0.521831	-7.127020
N	0.636071	-5.478164	-4.090192
C	1.879633	-5.998897	-3.500706
C	3.039572	-5.093875	-3.840021
O	3.272694	-4.845020	-5.012283
C	2.168908	-7.418289	-4.010261
O	1.128075	-8.278974	-3.642954
H	0.194545	-6.006283	-4.877527

H	1.755334	-6.061125	-2.396310
H	3.121946	-7.783005	-3.566850
H	2.273200	-7.410862	-5.119064
H	1.376745	-9.175895	-3.986993
N	3.823624	-4.576464	-2.853875
C	5.006609	-3.738552	-3.111568
C	6.268950	-4.461236	-2.706068
O	6.200667	-5.489498	-2.051778
C	4.907758	-2.407992	-2.343750
C	3.593288	-1.709686	-2.567156
C	2.716708	-1.493662	-1.492123
C	3.240956	-1.248099	-3.846536
C	1.508154	-0.825680	-1.692929
C	2.023580	-0.592599	-4.045473
C	1.162672	-0.376934	-2.967402
H	3.593946	-4.794196	-1.858191
H	5.092191	-3.508121	-4.196055
H	5.072818	-2.589314	-1.259525
H	5.717500	-1.733190	-2.683727
H	2.970969	-1.829104	-0.496176
H	3.907593	-1.394926	-4.686776
H	0.840973	-0.656860	-0.859985
H	1.749681	-0.245031	-5.033196
H	0.222849	0.130432	-3.121983
N	7.479218	-3.953561	-3.061180
C	8.752340	-4.542480	-2.627826
C	9.107972	-4.076505	-1.235527
O	9.436536	-4.908662	-0.405955
C	9.874541	-4.216583	-3.626920
O	10.051844	-2.831799	-3.753406
H	7.502965	-3.055532	-3.596443
H	8.657335	-5.652717	-2.619661
H	10.823096	-4.687322	-3.281991
H	9.611931	-4.648570	-4.618518
H	10.801916	-2.706681	-4.390828
N	9.075728	-2.753762	-0.910879
C	9.576707	-2.213962	0.364008
C	8.460097	-1.555127	1.143495
O	7.383285	-1.346611	0.606830
C	10.702649	-1.197713	0.090448
C	11.913248	-1.852873	-0.595873
C	13.042443	-0.887349	-0.851255
O	12.948271	0.285213	-0.526820
N	14.173398	-1.323337	-1.448404
H	8.743477	-2.065750	-1.624678
H	9.990809	-3.027080	0.999807
H	11.045537	-0.757744	1.051948
H	10.312075	-0.375190	-0.549572
H	11.601396	-2.284150	-1.570002
H	12.292958	-2.678864	0.043768
H	14.965338	-0.667428	-1.633510
H	14.274822	-2.322558	-1.735633
N	8.662455	-1.194310	2.441632
C	7.689546	-0.409022	3.219106

C	7.787701	1.079393	2.929441
O	6.909014	1.810977	3.355014
C	7.806979	-0.709510	4.729184
C	9.067032	-0.124766	5.393234
C	9.123591	-0.502691	6.877958
C	10.370729	0.084301	7.550281
N	10.417107	-0.278396	8.963904
H	9.596266	-1.393143	2.864294
H	6.668376	-0.736558	2.922979
H	6.908769	-0.299298	5.242266
H	7.791936	-1.810603	4.874671
H	9.979693	-0.507067	4.890815
H	9.056235	0.983223	5.304498
H	8.215585	-0.117626	7.392136
H	9.142104	-1.610526	6.976436
H	11.283987	-0.299477	7.043835
H	10.358773	1.192921	7.457187
H	11.264497	0.118128	9.428143
H	9.587623	0.080804	9.487983
H	10.451506	-1.313526	9.101900
N	8.813455	1.606200	2.202998
C	8.772803	2.968634	1.649581
C	7.770294	3.052441	0.519796
O	7.052291	4.034495	0.434108
C	10.164843	3.406615	1.168772
O	10.615335	2.582215	0.127847
H	9.593362	0.986121	1.895361
H	8.472988	3.675803	2.455564
H	10.117423	4.462116	0.817154
H	10.877281	3.354689	2.022462
H	11.510701	2.925180	-0.127431
N	7.659803	2.042640	-0.385495
C	6.638488	2.022347	-1.444082
C	5.251545	1.764994	-0.884339
O	4.286179	2.066151	-1.567753
C	6.983405	0.952668	-2.499532
C	8.267503	1.219961	-3.239878
O	8.921982	2.226643	-3.030037
O	8.659219	0.414232	-4.064501
H	8.269826	1.201441	-0.273158
H	6.624481	3.015237	-1.945240
H	6.161302	0.885688	-3.245802
H	7.072506	-0.031222	-1.992616
N	5.065477	1.212711	0.347944
C	3.740376	1.082700	0.971323
C	3.173618	2.444329	1.285158
O	2.061287	2.717639	0.873190
C	3.815856	0.197013	2.236792
C	2.500932	0.115792	3.051925
C	1.319569	-0.408195	2.223213
C	2.704199	-0.766524	4.288867
H	5.896046	0.911219	0.900653
H	3.069720	0.575423	0.249367
H	4.605164	0.601133	2.905134

H	4.129361	-0.826437	1.933541
H	2.244078	1.132637	3.418591
H	1.054415	0.303243	1.416410
H	1.553311	-1.394634	1.773209
H	0.424023	-0.507941	2.869492
H	3.540752	-0.376442	4.907313
H	1.785980	-0.767784	4.914618
H	2.935176	-1.810101	3.990494
N	3.878351	3.353021	2.013293
C	3.305103	4.644655	2.440841
C	2.942188	5.542485	1.269288
O	2.161741	6.459951	1.464467
C	4.220562	5.397057	3.448516
C	4.369006	4.621721	4.765715
C	5.603804	5.741562	2.872681
H	4.843239	3.093783	2.311404
H	2.352029	4.422076	2.969728
H	3.722652	6.359640	3.702390
H	4.983898	5.205155	5.483852
H	3.374195	4.456779	5.227882
H	4.856702	3.637804	4.603168
H	6.120706	6.458638	3.545290
H	6.238736	4.837418	2.788994
H	5.514291	6.217009	1.874009
N	3.447124	5.335806	0.021205
C	3.017766	6.114768	-1.151999
C	1.547118	5.889157	-1.419619
O	0.815836	6.853315	-1.574293
C	3.828365	5.722523	-2.404911
C	5.315720	6.082912	-2.274442
C	6.095385	5.644271	-3.519798
C	7.602069	5.859334	-3.332071
N	8.337392	5.438616	-4.521154
H	4.125636	4.557057	-0.127156
H	3.189962	7.196561	-0.951787
H	3.725768	4.629970	-2.587312
H	3.411693	6.254830	-3.289086
H	5.420408	7.182107	-2.141661
H	5.748805	5.583297	-1.383243
H	5.744118	6.229231	-4.397906
H	5.906020	4.566710	-3.719375
H	7.804438	6.935644	-3.136707
H	7.957759	5.271651	-2.456450
H	9.365696	5.571891	-4.396319
H	8.186405	4.427831	-4.738357
H	8.059973	5.985768	-5.366888
N	1.046897	4.625822	-1.484434
C	-0.346070	4.316370	-1.839927
C	-1.251802	4.213374	-0.627690
O	-2.446631	4.068086	-0.823422
C	-0.397256	3.014209	-2.651750
C	0.326448	3.173354	-3.957888
N	-0.222229	3.805908	-5.110756
C	1.579237	2.812078	-4.213685

C	0.767408	3.774777	-6.023547
N	1.885572	3.189745	-5.552233
H	1.698385	3.826498	-1.320220
H	-0.750895	5.127057	-2.481429
H	-1.448579	2.737079	-2.874619
H	0.053163	2.192839	-2.054418
H	-1.175461	4.214307	-5.228810
H	2.269704	2.338426	-3.524548
H	0.677167	4.179072	-7.022753
N	-0.790944	4.323200	0.650696
C	-1.710996	4.523269	1.782646
C	-2.194858	5.958990	1.848803
O	-3.168480	6.209511	2.539949
C	-1.056019	4.123812	3.119438
C	-0.637898	2.645284	3.141913
C	-0.394830	2.161896	4.543523
O	-1.113667	1.298728	5.016989
N	0.620048	2.665260	5.279612
H	0.223408	4.490255	0.823669
H	-2.604383	3.875459	1.641342
H	-1.789552	4.302036	3.937689
H	-0.168851	4.766935	3.310649
H	0.286513	2.509850	2.553439
H	-1.421066	2.019475	2.669511
H	1.250703	3.397499	4.887995
H	0.795562	2.322138	6.250549
N	-1.572213	6.954631	1.155454
C	-2.001815	8.360260	1.166764
C	-2.624593	8.772952	-0.156808
O	-3.124279	9.884581	-0.225396
C	-0.792897	9.262949	1.482323
C	-0.258724	9.027643	2.903401
C	0.957032	9.909648	3.215912
N	2.165248	9.380357	2.574234
C	2.740757	9.834672	1.421389
N	2.179809	10.825261	0.679856
N	3.895139	9.267192	0.993308
H	-0.709353	6.741772	0.608927
H	-2.770554	8.525907	1.953145
H	-1.092102	10.330643	1.392520
H	0.009795	9.065304	0.738969
H	-1.064380	9.266039	3.631791
H	0.018573	7.959530	3.041573
H	1.118980	9.896197	4.315588
H	0.766206	10.971126	2.962112
H	2.626211	8.586239	3.074039
H	2.639902	11.148719	-0.200338
H	1.270348	11.274110	0.913609
H	4.354574	9.579935	0.108887
H	4.351512	8.498131	1.532758
N	-2.634193	7.954097	-1.250792
C	-3.179240	8.357227	-2.560454
C	-4.259966	7.395678	-3.014243
O	-5.351687	7.846773	-3.322361

C	-2.054601	8.493777	-3.615801
O	-1.347890	7.289204	-3.764198
C	-1.065849	9.596633	-3.229559
H	-2.181636	7.016974	-1.196774
H	-3.657980	9.359815	-2.484529
H	-2.516191	8.796812	-4.586997
H	-1.732674	6.814954	-4.546914
H	-0.303531	9.712025	-4.028976
H	-1.594408	10.565926	-3.106840
H	-0.544628	9.344528	-2.281420
N	-4.032480	6.054139	-3.089162
C	-5.040994	5.093441	-3.564320
C	-6.221981	5.066797	-2.620873
O	-7.334533	5.309006	-3.060255
C	-4.414687	3.691022	-3.715428
C	-5.390006	2.673481	-4.238743
N	-5.770413	1.488316	-3.547614
C	-5.993367	2.689476	-5.421244
C	-6.602343	0.880136	-4.411610
N	-6.753620	1.585303	-5.549628
H	-3.100663	5.685449	-2.801349
H	-5.383306	5.420187	-4.572636
H	-4.028106	3.340808	-2.736741
H	-3.553916	3.746478	-4.415825
H	-5.463594	1.149423	-2.609625
H	-5.890831	3.452425	-6.181468
H	-7.085066	-0.068987	-4.221455
N	-6.046280	4.801657	-1.298610
C	-7.134862	4.890972	-0.313570
C	-7.239107	6.300852	0.218951
O	-8.333182	6.841554	0.233307
C	-6.949361	3.866942	0.831679
O	-5.673412	3.979688	1.409589
C	-7.143356	2.438791	0.319147
H	-5.073720	4.645563	-0.950782
H	-8.103257	4.644077	-0.807390
H	-7.738586	4.041428	1.602594
H	-5.751963	4.605507	2.175996
H	-6.982793	1.714318	1.144446
H	-8.178530	2.310682	-0.060814
H	-6.433747	2.210980	-0.502581
N	-6.137465	6.965408	0.664845
C	-6.173725	8.330579	1.192257
C	-5.688837	8.338959	2.574283
O	-5.292139	8.345767	3.702782
H	-5.520128	8.977879	0.574261
H	-7.197282	8.759708	1.174254
H	-5.201797	6.503576	0.606161
Au	-9.876028	-1.437556	1.406683
Cl	-10.897266	-2.245739	3.422966
N	-5.761386	-0.793378	2.386896
C	-5.398768	0.260000	3.139607
C	-6.067954	0.649830	4.300353
C	-7.167248	-0.095567	4.707400

C	-7.572893	-1.171601	3.926484
C	-6.842483	-1.471442	2.772066
C	-4.257001	1.068571	2.644605
H	-5.715664	1.523645	4.840376
H	-7.718595	0.171817	5.605142
H	-8.461948	-1.750972	4.166343
H	-7.170215	-2.278981	2.115001
O	-3.996865	2.167691	3.117334
C	-3.468434	0.514590	1.486465
C	-3.555275	1.156244	0.253740
C	-2.892563	0.659261	-0.862040
C	-2.083817	-0.462849	-0.732998
C	-1.920519	-1.074515	0.506847
C	-2.623929	-0.601679	1.616713
H	-4.175370	2.042451	0.173021
H	-3.013552	1.142426	-1.824817
H	-1.570757	-0.867720	-1.600768
H	-1.252934	-1.926902	0.599134

3/ZF TS1

N	4.963305	-9.229071	-0.199252
C	5.974705	-8.492085	0.560963
C	5.375613	-7.279496	1.245788
O	5.369895	-7.232391	2.464998
C	7.065639	-8.105929	-0.442030
C	6.456984	-8.301903	-1.829353
C	5.066482	-8.891707	-1.614300
H	3.991279	-9.075713	0.158150
H	5.110729	-10.257669	-0.073261
H	6.414570	-9.156741	1.341321
H	7.422828	-7.060826	-0.305815
H	7.938249	-8.785396	-0.322456
H	7.082858	-8.998549	-2.429041
H	6.387122	-7.331294	-2.367319
H	4.290324	-8.141077	-1.884981
H	4.913151	-9.792447	-2.250000
N	4.841519	-6.264376	0.515406
C	4.134328	-5.115839	1.091344
C	2.733078	-5.103241	0.531474
O	2.356301	-4.190243	-0.185659
C	4.934301	-3.833197	0.791498
C	6.165641	-3.771461	1.654677
C	6.057359	-3.411854	3.006631
C	7.422551	-4.125675	1.134371
C	7.193209	-3.413487	3.816890
C	8.518051	-4.120548	1.913518
C	8.440491	-3.763109	3.267519
O	9.543263	-3.765476	3.995918
H	4.902222	-6.300949	-0.526306
H	4.031388	-5.216260	2.194930
H	4.323647	-2.927276	0.973042
H	5.218497	-3.816486	-0.280159
H	5.097590	-3.143060	3.430011
H	7.513698	-4.421571	0.097205

H	7.100608	-3.144459	4.861355
H	9.473841	-4.400678	1.488600
H	9.397853	-3.495201	4.962728
N	1.903121	-6.139925	0.822956
C	0.618515	-6.376785	0.156850
C	-0.508647	-6.134245	1.130264
O	-0.495368	-6.687505	2.222896
C	0.598336	-7.812927	-0.402071
C	-0.662061	-8.109363	-1.226240
C	-0.589452	-9.503364	-1.864439
C	-1.833720	-9.820673	-2.710126
N	-2.969392	-10.213745	-1.877449
H	2.233258	-6.864622	1.501955
H	0.496836	-5.681550	-0.699258
H	0.674489	-8.543429	0.433758
H	1.492237	-7.945927	-1.051743
H	-0.771938	-7.341815	-2.024197
H	-1.544423	-8.062637	-0.557886
H	0.302361	-9.549958	-2.525198
H	-0.461544	-10.277382	-1.076280
H	-1.589593	-10.659083	-3.398971
H	-2.109971	-8.944093	-3.337176
H	-3.804336	-10.437378	-2.463579
H	-3.263312	-9.463249	-1.215172
H	-2.761238	-11.065299	-1.309190
N	-1.581048	-5.278715	0.744074
C	-2.625952	-5.089331	1.753706
C	-3.516177	-6.326859	1.922759
O	-3.513363	-7.190880	1.064731
C	-3.491102	-3.907729	1.267899
S	-4.816385	-3.386446	2.404423
H	-2.038393	-5.740292	-0.036420
H	-2.190690	-4.774289	2.711461
H	-2.789825	-3.089999	1.068297
H	-3.913730	-4.187350	0.292681
N	-4.413533	-6.404263	3.083494
C	-5.311678	-7.566032	3.068700
C	-6.505727	-7.316862	2.181255
O	-6.784041	-8.135828	1.320299
C	-5.714943	-7.802267	4.519255
C	-4.556647	-7.248283	5.333321
C	-3.659551	-6.523953	4.334700
H	-4.768801	-8.480168	2.726877
H	-6.645559	-7.250990	4.780911
H	-5.869256	-8.885092	4.720646
H	-3.998248	-8.070379	5.832549
H	-4.930481	-6.541664	6.106739
H	-3.397333	-5.513418	4.715733
H	-2.723778	-7.113148	4.189123
N	-7.259992	-6.194560	2.331983
C	-8.428730	-5.903304	1.491821
C	-7.978238	-5.492340	0.107553
O	-8.308246	-6.186524	-0.845299
C	-9.295275	-4.814675	2.150945

C	-10.594935	-4.545839	1.377531
C	-11.416659	-3.495215	2.063392
O	-11.637110	-2.432595	1.510342
O	-11.876018	-3.703700	3.172229
H	-6.963567	-5.485511	3.041374
H	-9.051050	-6.824888	1.419362
H	-8.708294	-3.877612	2.233386
H	-9.550888	-5.145343	3.182122
H	-10.354239	-4.207947	0.345691
H	-11.191805	-5.480646	1.304138
N	-7.208571	-4.306904	-0.078897
C	-6.859780	-3.983506	-1.449023
C	-6.061884	-5.018075	-2.245706
O	-6.341653	-5.280194	-3.400044
C	-6.037157	-2.684680	-1.505250
S	-6.888683	-1.223647	-0.838961
H	-6.426988	-4.274121	0.573463
H	-7.774353	-3.812786	-2.033724
H	-5.078788	-2.814806	-0.984522
H	-5.801004	-2.474416	-2.554476
N	-4.943658	-5.672663	-1.558224
C	-4.168754	-6.572003	-2.421802
C	-3.372649	-5.805342	-3.439951
O	-3.473452	-6.104674	-4.618276
H	-5.350749	-6.254946	-0.790811
H	-3.450206	-7.131748	-1.791020
H	-4.832218	-7.321951	-2.909487
N	-2.541294	-4.803554	-3.046786
C	-1.648019	-4.075787	-3.957674
C	-0.222501	-4.292689	-3.507355
O	0.145907	-3.822504	-2.443048
C	-2.013469	-2.578852	-3.970827
C	-3.340211	-2.331159	-4.711844
C	-3.906788	-0.917124	-4.496576
C	-2.971767	0.228631	-4.917322
N	-2.651146	0.185818	-6.341769
H	-2.509821	-4.547580	-2.033841
H	-1.751104	-4.456340	-4.997920
H	-2.086183	-2.202726	-2.926233
H	-1.207698	-2.018247	-4.491770
H	-4.104750	-3.051198	-4.348618
H	-3.204871	-2.524629	-5.797346
H	-4.860159	-0.828743	-5.060592
H	-4.154129	-0.791644	-3.423333
H	-3.476670	1.191992	-4.690143
H	-2.039717	0.202780	-4.319271
H	-2.116636	1.035037	-6.631358
H	-3.508816	0.149205	-6.937121
H	-2.056027	-0.635220	-6.589665
N	0.628989	-5.043611	-4.258525
C	1.970722	-5.437290	-3.803030
C	2.969531	-4.353772	-4.118629
O	2.962703	-3.837698	-5.224937
C	2.401077	-6.750632	-4.466905

O	1.496973	-7.766883	-4.134925
H	0.314729	-5.368904	-5.201529
H	1.949297	-5.621145	-2.705809
H	3.418261	-7.028630	-4.107943
H	2.435343	-6.625953	-5.573064
H	1.835029	-8.590560	-4.573077
N	3.876495	-3.969216	-3.180464
C	4.849183	-2.883930	-3.375033
C	6.254153	-3.349590	-3.062580
O	6.441955	-4.455826	-2.580919
C	4.449308	-1.681795	-2.495990
C	3.265768	-0.965511	-3.088823
C	1.995377	-1.068620	-2.499307
C	3.419043	-0.220946	-4.271015
C	0.890373	-0.468437	-3.108935
C	2.311902	0.377583	-4.874803
C	1.046915	0.240820	-4.302359
H	3.864750	-4.443715	-2.250458
H	4.866093	-2.567601	-4.441295
H	4.218249	-2.026235	-1.465998
H	5.284196	-0.961842	-2.405845
H	1.855533	-1.633125	-1.586052
H	4.393264	-0.121334	-4.733626
H	-0.089686	-0.567302	-2.665993
H	2.432444	0.938744	-5.792347
H	0.191714	0.690223	-4.785296
N	7.309162	-2.544002	-3.359933
C	8.709507	-2.940760	-3.160310
C	9.159070	-2.643834	-1.750179
O	9.785093	-3.497842	-1.144301
C	9.622763	-2.249959	-4.186061
O	9.553614	-0.855196	-4.062357
H	7.113793	-1.593247	-3.749483
H	8.809947	-4.035364	-3.342484
H	10.672399	-2.589254	-4.032879
H	9.306382	-2.547973	-5.210752
H	10.179750	-0.486746	-4.738181
N	8.899636	-1.438334	-1.172376
C	9.444103	-1.022596	0.130534
C	8.328834	-0.671864	1.091783
O	7.176061	-0.610993	0.694164
C	10.374542	0.190892	-0.065861
C	11.597160	-0.156275	-0.932122
C	12.537525	1.006798	-1.120812
O	12.300842	2.091220	-0.613761
N	13.656207	0.849244	-1.862070
H	8.352689	-0.728940	-1.710559
H	10.032829	-1.844476	0.593871
H	10.739038	0.545267	0.922666
H	9.805377	1.018899	-0.543683
H	11.260293	-0.492720	-1.934946
H	12.157383	-0.989983	-0.456076
H	14.318149	1.645092	-2.003874
H	13.877513	-0.069809	-2.306587

N	8.617085	-0.417582	2.398212
C	7.632643	0.132848	3.345044
C	7.283229	1.571817	3.024061
O	6.224681	2.020439	3.432860
C	8.116791	-0.012038	4.805113
C	9.269529	0.937961	5.181334
C	9.771018	0.652736	6.601512
C	10.908459	1.606546	6.986862
N	11.382906	1.328304	8.339433
H	9.607358	-0.529010	2.711506
H	6.700336	-0.469119	3.255057
H	7.258075	0.178931	5.486148
H	8.441270	-1.061130	4.969544
H	10.113435	0.819828	4.469570
H	8.915083	1.990446	5.130814
H	8.931577	0.774118	7.321003
H	10.137776	-0.395714	6.659919
H	11.752687	1.489689	6.271673
H	10.548569	2.658005	6.934321
H	12.157877	1.974727	8.608462
H	10.627625	1.446614	9.051658
H	11.752699	0.355523	8.432559
N	8.131239	2.365468	2.312123
C	7.761361	3.710824	1.852002
C	6.661561	3.638003	0.819638
O	5.716348	4.400346	0.913859
C	8.981200	4.458222	1.289797
O	9.509538	3.787530	0.177898
H	9.062086	1.988383	2.027399
H	7.400012	4.297874	2.726703
H	8.678300	5.489210	0.997073
H	9.757949	4.534955	2.083362
H	10.278486	4.332098	-0.132936
N	6.712651	2.731016	-0.193707
C	5.677357	2.634596	-1.235487
C	4.414153	1.956505	-0.731929
O	3.405471	2.035466	-1.415221
C	6.227890	1.893784	-2.471116
C	7.381270	2.594999	-3.138989
O	7.802094	3.660045	-2.722318
O	7.904894	2.086423	-4.113514
H	7.497957	2.041506	-0.209747
H	5.395633	3.662056	-1.552390
H	5.417786	1.774016	-3.222602
H	6.569064	0.884524	-2.163619
N	4.372665	1.298643	0.461443
C	3.113517	0.862510	1.085210
C	2.299227	2.072734	1.490730
O	1.129136	2.136746	1.154110
C	3.404569	-0.027361	2.317873
C	2.133699	-0.678063	2.925719
C	1.722328	-1.949228	2.172104
C	2.352541	-1.002635	4.408607
H	5.245743	1.205316	1.023315

H	2.542797	0.259622	0.344694
H	3.908785	0.598721	3.086519
H	4.131708	-0.819989	2.039528
H	1.285838	0.033017	2.867823
H	1.606015	-1.747777	1.087429
H	2.475205	-2.748817	2.321301
H	0.749662	-2.322836	2.556930
H	2.570221	-0.072685	4.976345
H	1.437147	-1.460385	4.841291
H	3.202980	-1.706751	4.533892
N	2.861210	3.074836	2.223016
C	2.102988	4.245217	2.701220
C	1.821914	5.253352	1.600166
O	1.002887	6.130655	1.822715
C	2.788109	4.929792	3.919406
C	2.897777	3.971230	5.113204
C	4.164556	5.533993	3.591143
H	3.880682	3.013076	2.432949
H	1.112381	3.883994	3.056234
H	2.133519	5.769934	4.244463
H	3.356105	4.491747	5.981031
H	1.888435	3.627958	5.419218
H	3.520299	3.086292	4.862053
H	4.501803	6.173252	4.434613
H	4.927897	4.744465	3.440688
H	4.114799	6.172440	2.684522
N	2.440901	5.199417	0.386732
C	2.066112	6.090646	-0.724031
C	0.683301	5.767061	-1.237702
O	0.015304	6.673040	-1.705827
C	3.082782	6.012150	-1.879854
C	4.418973	6.666285	-1.506418
C	5.411718	6.606269	-2.673339
C	6.794572	7.111353	-2.244079
N	7.714763	7.132123	-3.377565
H	3.134081	4.443911	0.195265
H	2.051290	7.137838	-0.350450
H	3.249022	4.949991	-2.161807
H	2.671880	6.540714	-2.769148
H	4.247547	7.730195	-1.232643
H	4.848340	6.149968	-0.626145
H	5.030335	7.233994	-3.508331
H	5.505360	5.560640	-3.038394
H	6.706030	8.138923	-1.826857
H	7.203431	6.445429	-1.451404
H	8.665031	7.453214	-3.086624
H	7.831766	6.187283	-3.807157
H	7.392288	7.780081	-4.131068
N	0.188375	4.498722	-1.189748
C	-1.156790	4.130496	-1.655522
C	-2.091589	3.805812	-0.499450
O	-3.230195	3.458260	-0.761693
C	-1.063233	2.960841	-2.650225
C	-0.360649	3.370464	-3.915108

N	-1.003538	3.545091	-5.175239
C	0.945433	3.571324	-4.063812
C	-0.010465	3.857251	-6.029408
N	1.194444	3.891645	-5.428820
H	0.794598	3.729315	-0.828857
H	-1.622884	4.981127	-2.197491
H	-2.086417	2.627792	-2.925347
H	-0.530163	2.101715	-2.190157
H	-2.017144	3.445410	-5.401552
H	1.706594	3.468046	-3.299559
H	-0.161939	4.053297	-7.082304
N	-1.731418	3.983504	0.804476
C	-2.737420	4.177168	1.863950
C	-3.285790	5.596151	1.857493
O	-4.233281	5.851955	2.582347
C	-2.145747	3.858301	3.250992
C	-1.792960	2.373966	3.394859
C	-1.354541	2.062442	4.798209
O	-2.165412	2.116533	5.707115
N	-0.074954	1.720508	5.064290
H	-0.753113	4.258292	1.034982
H	-3.590068	3.482759	1.693147
H	-2.895384	4.117286	4.031882
H	-1.242075	4.481592	3.429697
H	-0.991923	2.112096	2.673716
H	-2.682096	1.753234	3.156266
H	0.646035	1.709187	4.311129
H	0.224361	1.497276	6.039927
N	-2.746361	6.573364	1.074382
C	-3.224400	7.963515	1.049415
C	-3.890712	8.263449	-0.274319
O	-4.993582	8.784481	-0.275125
C	-2.047378	8.927731	1.288823
C	-1.508200	8.808761	2.722824
C	-0.357651	9.785393	2.992938
N	0.892027	9.302219	2.396217
C	1.502593	9.806070	1.283594
N	0.886764	10.706032	0.473536
N	2.749104	9.379414	0.964899
H	-1.902975	6.365212	0.497721
H	-3.972963	8.138907	1.855031
H	-2.394081	9.972014	1.123462
H	-1.233186	8.713512	0.562099
H	-2.331821	9.034731	3.435057
H	-1.165073	7.769559	2.920619
H	-0.209527	9.849582	4.092781
H	-0.617358	10.814023	2.672509
H	1.390534	8.559185	2.937447
H	1.375541	11.073293	-0.373382
H	-0.096930	11.018806	0.608834
H	3.240218	9.737651	0.115523
H	3.247628	8.676566	1.555696
N	-3.270293	7.981283	-1.451232
C	-3.850057	8.311341	-2.762219

C	-4.955677	7.343718	-3.121740
O	-6.019854	7.787117	-3.522661
C	-2.765774	8.345620	-3.869654
O	-2.092914	7.114123	-3.958086
C	-1.740105	9.451709	-3.607660
H	-2.354865	7.484884	-1.425979
H	-4.289767	9.334315	-2.710386
H	-3.258826	8.585725	-4.842617
H	-2.574135	6.562401	-4.628584
H	-1.004793	9.483785	-4.439425
H	-2.243833	10.439945	-3.547954
H	-1.190080	9.267814	-2.660370
N	-4.769596	5.999760	-3.020787
C	-5.763591	5.020757	-3.481744
C	-6.898129	4.915046	-2.489717
O	-8.042586	5.068635	-2.885017
C	-5.094281	3.651138	-3.707667
C	-6.041407	2.638722	-4.287099
N	-6.476804	1.463219	-3.613950
C	-6.576920	2.657843	-5.501558
C	-7.269193	0.863125	-4.519227
N	-7.345286	1.564027	-5.667605
H	-3.888307	5.646999	-2.584877
H	-6.164981	5.359125	-4.464733
H	-4.704698	3.257788	-2.748870
H	-4.232585	3.769079	-4.398185
H	-6.241416	1.135547	-2.652621
H	-6.424239	3.418401	-6.255849
H	-7.780004	-0.075114	-4.348340
N	-6.655107	4.640470	-1.179438
C	-7.730753	4.419048	-0.200624
C	-8.194290	5.733173	0.382746
O	-9.391220	5.967631	0.424638
C	-7.296671	3.436024	0.913414
O	-6.223007	3.953477	1.654824
C	-6.885551	2.083602	0.334600
H	-5.664301	4.610217	-0.848455
H	-8.598929	3.944343	-0.714281
H	-8.173613	3.250444	1.580369
H	-6.606873	4.407678	2.449827
H	-6.651898	1.385941	1.164461
H	-7.714033	1.654664	-0.267739
H	-5.981459	2.182155	-0.302755
N	-7.297654	6.642435	0.854160
C	-7.680890	7.894551	1.509739
C	-7.009682	7.995482	2.808420
O	-6.459484	8.076862	3.867470
H	-7.372455	8.751130	0.876884
H	-8.776906	7.960480	1.673759
H	-6.275764	6.451387	0.745627
Au	-5.983332	-1.055830	1.365146
Cl	-8.253990	-1.143166	2.284012
N	-5.318627	-0.257131	3.490752
C	-4.165957	-0.710232	4.001937

C	-3.896860	-0.608597	5.369240
C	-4.830906	-0.011120	6.200774
C	-6.011341	0.479437	5.650216
C	-6.223643	0.320609	4.285879
C	-3.063645	-1.206786	3.111785
H	-2.947705	-0.996021	5.722185
H	-4.639064	0.078855	7.267453
H	-6.769063	0.960236	6.260576
H	-7.149635	0.619287	3.801869
O	-2.064326	-1.690082	3.647199
C	-3.006659	-0.760632	1.683060
C	-1.704483	-0.456632	1.253131
C	-1.443944	-0.017496	-0.033376
C	-2.505366	0.119020	-0.920333
C	-3.803739	-0.176402	-0.516689
C	-4.075102	-0.626224	0.776552
H	-0.908767	-0.588849	1.977552
H	-0.424773	0.210642	-0.334354
H	-2.337263	0.448751	-1.940205
H	-4.629501	-0.065550	-1.211653

3/ZF TS2

N	6.245540	-8.278041	-1.285452
C	7.034492	-7.885365	-0.109445
C	6.184248	-7.183855	0.928166
O	6.117878	-7.648806	2.054216
C	8.174215	-6.991325	-0.619459
C	8.138728	-7.118493	-2.133835
C	6.719542	-7.548330	-2.454917
H	5.214498	-8.147519	-1.159596
H	6.339335	-9.307666	-1.448331
H	7.484950	-8.795795	0.351959
H	8.025947	-5.925294	-0.341328
H	9.156624	-7.323482	-0.218437
H	8.848874	-7.910259	-2.459208
H	8.398320	-6.160954	-2.632933
H	6.088480	-6.646246	-2.620851
H	6.686430	-8.182099	-3.368955
N	5.495706	-6.056269	0.604304
C	4.583794	-5.355371	1.516768
C	3.221667	-5.285092	0.867869
O	2.674338	-4.208153	0.693934
C	5.158786	-3.962948	1.845348
C	6.491887	-4.084503	2.536791
C	6.555213	-4.422863	3.897116
C	7.685528	-3.895189	1.818531
C	7.797534	-4.548709	4.522779
C	8.881128	-4.013864	2.420302
C	8.976857	-4.335585	3.782064
O	10.227331	-4.432602	4.354391
H	5.583394	-5.677480	-0.364316
H	4.461179	-5.916619	2.469350
H	4.458729	-3.403111	2.503724
H	5.267302	-3.374524	0.906964

H	5.648802	-4.583559	4.467467
H	7.648348	-3.656329	0.763186
H	7.840006	-4.801858	5.574486
H	9.783867	-3.858010	1.842862
H	10.120962	-4.653914	5.315331
N	2.616814	-6.433216	0.461966
C	1.349468	-6.449420	-0.277447
C	0.199243	-6.244111	0.675989
O	0.126188	-6.932630	1.686661
C	1.211061	-7.772607	-1.059412
C	-0.036300	-7.797556	-1.956684
C	-0.040879	-9.018775	-2.885190
C	-1.135834	-8.927123	-3.960785
N	-2.477291	-8.986290	-3.383458
H	3.067483	-7.346425	0.702304
H	1.360081	-5.619321	-1.017501
H	1.173944	-8.630468	-0.352062
H	2.117045	-7.892856	-1.694135
H	-0.074210	-6.873049	-2.566334
H	-0.939431	-7.832483	-1.315461
H	0.939963	-9.092202	-3.402788
H	-0.177080	-9.945379	-2.285994
H	-1.008070	-9.776776	-4.666831
H	-1.020342	-7.985674	-4.542728
H	-3.206125	-9.030035	-4.130177
H	-2.697297	-8.145163	-2.806561
H	-2.610278	-9.828199	-2.779418
N	-0.791625	-5.265209	0.368359
C	-1.862382	-5.146823	1.353863
C	-2.820661	-6.360204	1.362203
O	-2.732257	-7.185384	0.474421
C	-2.718178	-3.905687	1.005783
S	-2.994377	-2.830043	2.441378
H	-1.229127	-5.569529	-0.495761
H	-1.424161	-4.977226	2.346008
H	-2.194002	-3.337456	0.227865
H	-3.694077	-4.206402	0.614337
N	-3.884624	-6.444393	2.377703
C	-4.610580	-7.725618	2.346530
C	-5.702097	-7.727601	1.301730
O	-5.732286	-8.628808	0.479771
C	-5.177249	-7.937417	3.749217
C	-4.295083	-7.100682	4.658047
C	-3.326213	-6.378113	3.733257
H	-3.904863	-8.569773	2.152352
H	-6.229971	-7.585840	3.831920
H	-5.139118	-9.012074	4.033501
H	-3.744538	-7.742021	5.380748
H	-4.911116	-6.365709	5.221638
H	-3.214420	-5.319993	4.050645
H	-2.332668	-6.882904	3.786762
N	-6.654212	-6.756904	1.295712
C	-7.910382	-6.847082	0.538919
C	-7.946400	-5.786543	-0.538607

O	-8.130456	-6.134711	-1.697412
C	-9.099562	-6.701110	1.508267
C	-10.449270	-6.960720	0.822253
C	-11.578618	-6.842480	1.802093
O	-12.418105	-5.970469	1.666464
O	-11.667805	-7.625013	2.731476
H	-6.551844	-5.967346	1.968648
H	-7.994764	-7.847204	0.056078
H	-9.099590	-5.683596	1.959041
H	-8.969367	-7.435292	2.334302
H	-10.597040	-6.227984	-0.001139
H	-10.460502	-7.981242	0.381750
N	-7.884778	-4.404352	-0.201696
C	-7.510414	-3.497123	-1.268388
C	-6.372600	-3.866607	-2.212516
O	-6.352034	-3.490146	-3.368308
C	-7.122639	-2.069079	-0.692267
S	-7.421773	-1.752599	1.070538
H	-7.371770	-4.236844	0.656788
H	-8.371855	-3.339722	-1.934757
H	-6.072091	-1.840897	-0.911645
H	-7.693922	-1.326807	-1.254992
N	-5.245686	-4.607585	-1.649569
C	-4.782996	-5.693792	-2.528549
C	-3.858309	-5.199064	-3.618264
O	-4.173386	-5.367232	-4.784696
H	-4.474352	-3.929463	-1.500787
H	-4.228146	-6.428434	-1.908768
H	-5.650534	-6.236182	-2.967019
N	-2.688514	-4.578315	-3.299910
C	-1.744405	-4.035146	-4.288825
C	-0.333193	-4.401342	-3.892505
O	0.065260	-4.110913	-2.776011
C	-1.876931	-2.500754	-4.366218
C	-3.106742	-2.073630	-5.181877
C	-3.312421	-0.549362	-5.217777
C	-2.158127	0.258296	-5.837546
N	-1.897930	-0.125657	-7.223022
H	-2.453407	-4.447403	-2.290141
H	-1.950179	-4.460471	-5.296143
H	-1.944499	-2.077635	-3.340620
H	-0.966835	-2.089611	-4.854132
H	-4.014538	-2.521781	-4.724636
H	-3.032104	-2.467908	-6.217638
H	-4.236978	-0.340093	-5.795884
H	-3.482885	-0.182639	-4.185859
H	-2.437711	1.334337	-5.808598
H	-1.232312	0.149926	-5.235729
H	-1.212066	0.523372	-7.669300
H	-2.763052	-0.101642	-7.808233
H	-1.489418	-1.083461	-7.298274
N	0.495872	-5.024233	-4.773710
C	1.891844	-5.351892	-4.449302
C	2.718570	-4.090255	-4.382103

O	2.764172	-3.355447	-5.355888
C	2.481474	-6.315115	-5.488046
O	1.744601	-7.506039	-5.502689
H	0.153250	-5.204671	-5.745184
H	1.922044	-5.874096	-3.468759
H	3.539777	-6.539068	-5.222384
H	2.458023	-5.847739	-6.498662
H	2.174381	-8.088168	-6.181692
N	3.405875	-3.780996	-3.248948
C	4.265143	-2.596719	-3.108861
C	5.697993	-3.036752	-2.953569
O	5.979832	-3.857069	-2.095557
C	3.830019	-1.769756	-1.886812
C	2.480984	-1.136446	-2.107093
C	1.356855	-1.591900	-1.402111
C	2.335467	-0.081288	-3.023237
C	0.110400	-0.994897	-1.601046
C	1.085492	0.508258	-3.226471
C	-0.025477	0.055584	-2.512117
H	3.345831	-4.429764	-2.430363
H	4.189641	-1.947696	-4.008424
H	3.815437	-2.418440	-0.984401
H	4.569058	-0.965615	-1.687937
H	1.439771	-2.418536	-0.712092
H	3.188106	0.279949	-3.582559
H	-0.749548	-1.358203	-1.055708
H	0.978123	1.313040	-3.941008
H	-0.991548	0.513114	-2.674798
N	6.670251	-2.518592	-3.752426
C	8.079272	-2.930098	-3.659842
C	8.637862	-2.644031	-2.285322
O	9.257626	-3.521833	-1.706920
C	8.928936	-2.248780	-4.745032
O	8.872737	-0.853593	-4.621864
H	6.402918	-1.804935	-4.468598
H	8.134265	-4.025355	-3.849928
H	9.984629	-2.592498	-4.657566
H	8.547554	-2.549366	-5.746596
H	9.456009	-0.489746	-5.337369
N	8.438331	-1.434209	-1.694717
C	8.972804	-1.074165	-0.373379
C	7.844795	-0.770294	0.587587
O	6.706238	-0.632291	0.168130
C	9.905523	0.144077	-0.505112
C	11.107082	-0.150055	-1.419827
C	12.067726	1.006753	-1.525952
O	11.862611	2.047495	-0.922979
N	13.170105	0.892246	-2.298857
H	7.890198	-0.705416	-2.206955
H	9.559008	-1.915409	0.057126
H	10.290573	0.421289	0.500254
H	9.332860	1.006569	-0.912809
H	10.745756	-0.393077	-2.441887
H	11.658696	-1.031562	-1.026975

H	13.845993	1.684441	-2.384034
H	13.364849	0.010784	-2.824523
N	8.102362	-0.652560	1.918793
C	7.101700	-0.167773	2.883322
C	6.929693	1.336932	2.812324
O	5.961136	1.836813	3.360239
C	7.428325	-0.640760	4.315119
C	8.643886	0.057513	4.950954
C	8.971092	-0.561063	6.315051
C	10.178027	0.130506	6.960278
N	10.487065	-0.468175	8.255680
H	9.085638	-0.794900	2.240143
H	6.120856	-0.626851	2.622222
H	6.538315	-0.468519	4.960187
H	7.606514	-1.734793	4.288642
H	9.532812	-0.035975	4.293477
H	8.421873	1.138439	5.086235
H	8.089911	-0.462527	6.986504
H	9.196808	-1.642763	6.185011
H	11.063502	0.036246	6.293261
H	9.958408	1.212221	7.099713
H	11.308430	-0.001675	8.701262
H	9.689888	-0.384946	8.925973
H	10.722407	-1.482780	8.172190
N	7.818015	2.127708	2.146646
C	7.542579	3.534858	1.827727
C	6.407095	3.644837	0.835709
O	5.568365	4.511995	0.998539
C	8.799860	4.234519	1.287816
O	9.205827	3.664052	0.073468
H	8.683491	1.703979	1.748175
H	7.260408	4.062391	2.766099
H	8.583884	5.316207	1.140857
H	9.618759	4.142639	2.035875
H	9.990872	4.190033	-0.229026
N	6.312721	2.790558	-0.220067
C	5.228055	2.858350	-1.213901
C	3.906459	2.363586	-0.653755
O	2.879042	2.687893	-1.225280
C	5.599812	2.056821	-2.478733
C	6.830093	2.566768	-3.183181
O	7.407619	3.570757	-2.803012
O	7.251465	1.968082	-4.156096
H	7.023366	2.030293	-0.313689
H	5.087834	3.919596	-1.514978
H	4.755105	2.096569	-3.199680
H	5.767416	0.994379	-2.202192
N	3.842468	1.579819	0.458180
C	2.571546	1.227850	1.113644
C	1.962581	2.450814	1.768607
O	0.781836	2.694151	1.580386
C	2.804908	0.098984	2.145864
C	1.548059	-0.318569	2.951684
C	0.453487	-0.920248	2.059554

C	1.939906	-1.327542	4.038649
H	4.729962	1.273593	0.913968
H	1.875756	0.841704	0.336874
H	3.585675	0.436453	2.862947
H	3.211710	-0.790338	1.613822
H	1.127348	0.571969	3.467387
H	0.071481	-0.169126	1.338659
H	0.839863	-1.802519	1.509453
H	-0.406374	-1.245946	2.680799
H	2.700827	-0.886048	4.717268
H	1.052546	-1.598635	4.649959
H	2.357769	-2.251447	3.583507
N	2.712354	3.260996	2.566169
C	2.159258	4.431843	3.273522
C	1.919870	5.613789	2.348007
O	1.211608	6.525502	2.744521
C	3.038289	4.845593	4.489793
C	3.195743	3.692589	5.492116
C	4.420712	5.389675	4.089289
H	3.732117	3.059060	2.643869
H	1.164867	4.144591	3.681311
H	2.506574	5.665275	5.023515
H	3.750685	4.040634	6.389335
H	2.202215	3.333846	5.826080
H	3.750828	2.841798	5.043798
H	4.940709	5.789415	4.985764
H	5.055330	4.593432	3.654604
H	4.327747	6.219579	3.358464
N	2.480561	5.681081	1.109060
C	2.184390	6.758098	0.150025
C	0.869120	6.505854	-0.551303
O	0.225668	7.463454	-0.948791
C	3.318976	6.881859	-0.884469
C	4.603618	7.432918	-0.245198
C	5.811204	7.244518	-1.170030
C	7.094673	7.764204	-0.511900
N	8.259198	7.468789	-1.341644
H	3.060587	4.884016	0.769030
H	2.108501	7.726405	0.694955
H	3.510004	5.884642	-1.338464
H	3.011462	7.568123	-1.704279
H	4.468964	8.514319	-0.026244
H	4.800382	6.918692	0.718602
H	5.638067	7.789087	-2.123957
H	5.935418	6.164550	-1.398617
H	7.016936	8.863803	-0.362712
H	7.226676	7.286365	0.483715
H	9.136294	7.828791	-0.903662
H	8.390140	6.441343	-1.480988
H	8.188067	7.907737	-2.287082
N	0.402207	5.239757	-0.736513
C	-0.908105	4.934017	-1.324829
C	-1.970286	4.736089	-0.254119
O	-3.106333	4.482520	-0.614298

C	-0.796409	3.699986	-2.242139
C	-0.071705	4.015916	-3.522839
N	-0.664154	3.941067	-4.817587
C	1.205330	4.366751	-3.652481
C	0.325114	4.264852	-5.671347
N	1.482888	4.534977	-5.039234
H	0.966825	4.432089	-0.394152
H	-1.248113	5.783578	-1.956320
H	-1.811173	3.343559	-2.517901
H	-0.277995	2.871299	-1.713943
H	-1.647557	3.693246	-5.062911
H	1.927897	4.500246	-2.857004
H	0.204358	4.303583	-6.745584
N	-1.717865	4.918926	1.074651
C	-2.807229	5.180598	2.032862
C	-3.470143	6.511434	1.744073
O	-4.638027	6.665724	2.060891
C	-2.297376	5.163150	3.490069
C	-2.058273	3.731861	3.982106
C	-1.622764	3.694819	5.420840
O	-2.286660	4.265702	6.269701
N	-0.525870	2.996639	5.789567
H	-0.744742	5.115384	1.392352
H	-3.577107	4.385158	1.919740
H	-3.062292	5.632318	4.148924
H	-1.363833	5.760529	3.580732
H	-1.291433	3.249682	3.340300
H	-3.005688	3.157528	3.896950
H	0.028087	2.453520	5.090402
H	-0.233088	2.958267	6.791677
N	-2.783868	7.526418	1.152791
C	-3.410593	8.797176	0.766713
C	-4.274636	8.612619	-0.459166
O	-5.403884	9.074167	-0.458851
C	-2.339878	9.864870	0.472067
C	-1.519604	10.226061	1.719386
C	-0.454721	11.285580	1.414313
N	0.610848	10.728197	0.573605
C	0.784852	10.946535	-0.764106
N	-0.132646	11.617561	-1.508481
N	1.891869	10.456757	-1.372978
H	-1.782774	7.378654	0.895073
H	-4.041821	9.168051	1.606727
H	-2.841586	10.785220	0.098804
H	-1.662779	9.493042	-0.326553
H	-2.203370	10.625539	2.499741
H	-1.021747	9.321847	2.131986
H	-0.002609	11.610281	2.376367
H	-0.910387	12.197498	0.980670
H	1.315853	10.138651	1.072481
H	0.023674	11.762856	-2.531000
H	-1.039238	11.961608	-1.130789
H	2.049088	10.590576	-2.396874
H	2.620236	9.941728	-0.831443

N	-3.789701	7.984706	-1.565660
C	-4.426993	8.092626	-2.887899
C	-5.324442	6.905181	-3.158017
O	-6.488458	7.110918	-3.463129
C	-3.371944	8.283263	-4.007659
O	-2.460112	7.213315	-4.034607
C	-2.591562	9.586475	-3.814367
H	-2.859626	7.513976	-1.503182
H	-5.069343	9.003642	-2.912023
H	-3.905492	8.365358	-4.985679
H	-2.769209	6.578294	-4.732382
H	-1.887407	9.729901	-4.661145
H	-3.284612	10.454027	-3.784444
H	-2.004440	9.558319	-2.872110
N	-4.855784	5.629123	-3.082122
C	-5.670745	4.461796	-3.455053
C	-6.823333	4.286489	-2.492477
O	-7.963214	4.270322	-2.928484
C	-4.807177	3.183600	-3.502948
C	-5.606596	1.992871	-3.957471
N	-5.955549	0.884816	-3.133916
C	-6.134124	1.811233	-5.162319
C	-6.690104	0.111113	-3.952881
N	-6.812319	0.648099	-5.182409
H	-3.864219	5.476911	-2.794778
H	-6.071877	4.634308	-4.480082
H	-4.382002	2.971308	-2.498997
H	-3.958361	3.331398	-4.203891
H	-5.727458	0.714470	-2.130346
H	-6.049479	2.484060	-6.005388
H	-7.136151	-0.828724	-3.659583
N	-6.594452	4.127431	-1.162502
C	-7.669786	3.888013	-0.190204
C	-8.362797	5.184690	0.159044
O	-9.581138	5.199614	0.226913
C	-7.137514	3.176239	1.077564
O	-6.173698	3.962773	1.729717
C	-6.517517	1.819958	0.732183
H	-5.619114	4.238564	-0.808692
H	-8.422880	3.204946	-0.647343
H	-7.997765	2.977304	1.761159
H	-6.656700	4.545624	2.371890
H	-6.227050	1.292905	1.664361
H	-7.247678	1.188996	0.182914
H	-5.609402	1.948614	0.107275
N	-7.645658	6.320315	0.383308
C	-8.249038	7.608480	0.728916
C	-7.678618	8.097904	1.986601
O	-7.210922	8.497214	3.012638
H	-8.031275	8.340898	-0.075495
H	-9.350797	7.536479	0.844143
H	-6.606347	6.295565	0.276338
Au	-5.336548	-2.368993	2.142854
Cl	-6.450845	-3.928250	3.806640

N	-5.520345	-0.164001	3.942571
C	-4.256790	-0.241834	4.388645
C	-3.925394	-0.622696	5.690062
C	-4.951163	-0.976510	6.555422
C	-6.263240	-0.914520	6.097818
C	-6.494795	-0.496625	4.789709
C	-3.170424	0.174778	3.450268
H	-2.879516	-0.638700	5.979496
H	-4.732722	-1.306261	7.568041
H	-7.095538	-1.207633	6.729959
H	-7.505802	-0.454385	4.387436
O	-2.126329	0.623146	3.919869
C	-3.351043	0.058920	1.983449
C	-2.810651	1.090989	1.215152
C	-2.782226	1.049471	-0.170208
C	-3.244928	-0.096798	-0.805496
C	-3.714802	-1.169139	-0.066522
C	-3.820324	-1.125829	1.337598
H	-2.402345	1.944384	1.744177
H	-2.394677	1.881452	-0.741848
H	-3.244216	-0.170870	-1.888068
H	-4.051008	-2.046417	-0.596104
